# Electronic‐State Programming of Ultrasmall CeO_2_ Switches H_2_O_2_ Activation From Radical to Bio‐Inspired Nonradical ^1^O_2_ Generation for Combating Antibiotic Resistance

**DOI:** 10.1002/advs.78122

**Published:** 2026-09-29

**Authors:** Bo Yuan, Xinyu Wu, Yin‐Song Liao, Chao Zhao, Zhanping Xiao, Wanqing Dai, Jyh‐Pin Chou, Wenchao Peng, Jian Lin Chen, Pi‐Tai Chou, Yung‐Kang Peng

**Affiliations:** ^1^ Department of Chemistry City University of Hong Kong Hong Kong Hong Kong SAR China; ^2^ Department of Applied Science School of Science and Technology Hong Kong Metropolitan University Hong Kong Hong Kong SAR China; ^3^ Graduate School of Advanced Technology National Taiwan University Taipei Taiwan; ^4^ Department of Chemistry National Taiwan University Taipei Taiwan; ^5^ School of Chemical Engineering and Technology Tianjin University Tianjin China

**Keywords:** antibiotic resistance, bio‐inspired ^1^O_2_ production, electronic state optimization, scalable production, ultrasmall CeO_2_ catalysts

## Abstract

Antibiotic resistance, driven by antibiotic‐resistant bacteria (ARB) and genes (ARGs) in aquatic systems, is an escalating threat. Conventional advanced oxidation processes (AOPs) based on OH radicals are limited by rapid radical scavenging. ^1^O_2_ is a promising alternative, but current H_2_O_2_‐to‐^1^O_2_ routes rely on HO_2_ radical chain‐termination reactions and suffer from short radical lifetimes and equilibrium with ^3^O_2_, leading to poor conversion efficiency. Inspired by bromoperoxidases (BPOs), we report a facile, scalable synthesis of carbon framework‐supported ultrasmall CeO_2_ nanoparticles (NPs). This material features electron‐lean Ce sites that activate H_2_O_2_ exclusively via a nonradical pathway, enabling selective HOBr production and outperforming BPOs in subsequent conversion to ^1^O_2_. A counterpart with electron‐rich Ce sites that activate H_2_O_2_ only via the conventional radical pathway was further prepared for comparison. Degradation of various contaminants (dyes, antibiotics, and ARB/ARG) reveals that the nonradical pathway far outperforms the radical pathway in both H_2_O_2_ utilization and removal efficiency. The optimized sample exhibits high ARB/ARG removal efficiency in a continuous‐flow reactor for 10 h. This work demonstrates a bio‐inspired route for nonradical H_2_O_2_‐to‐^1^O_2_ conversion and a scalable method for preparing catalysts with optimized electronic states and enhanced performance, guiding their design to combat antibiotic resistance.

## Graphical abstract

A scalable synthesis of carbon‐supported ultrasmall CeO_2_ nanoparticles with precisely tunable Ce electron density is reported. Electron‐lean Ce sites activate H_2_O_2_ exclusively via a nonradical pathway, enabling efficient HOBr and ^1^O_2_ production analogous to bromoperoxidase for contaminant and antibiotic resistance removal. This bio‐inspired approach outperforms conventional radical‐based systems in both H_2_O_2_ utilization and overall performance.

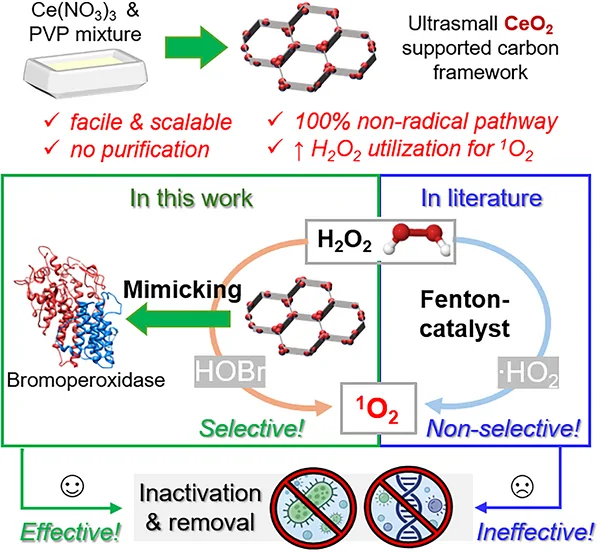

## 1 Introduction

Antibiotic resistance is acknowledged as a significant threat to public health and environmental sustainability today [1]. Improper management of human and livestock waste, along with pharmaceutical wastewater, leads to the accumulation of antibiotics in aquatic environments (Scheme 1a‐i) [2]. The selective pressure exerted by these antibiotics accelerates the evolution of resistant strains, resulting in the proliferation of antibiotic‐resistant bacteria (ARB). Effective remediation requires not only the inactivation of ARB but also the removal of their antibiotic resistance genes (ARGs). If left unaddressed, these genes can undergo horizontal gene transfer among microbial populations through conjugation and transformation, converting harmless environmental bacteria into resistant strains (Scheme 1a‐ii) [3]. As wastewater treatment plants have become breeding grounds and point sources for ARBs and ARGs, developing effective treatment techniques is imperative to prevent their environmental release [4, 5].

**SCHEME 1:**
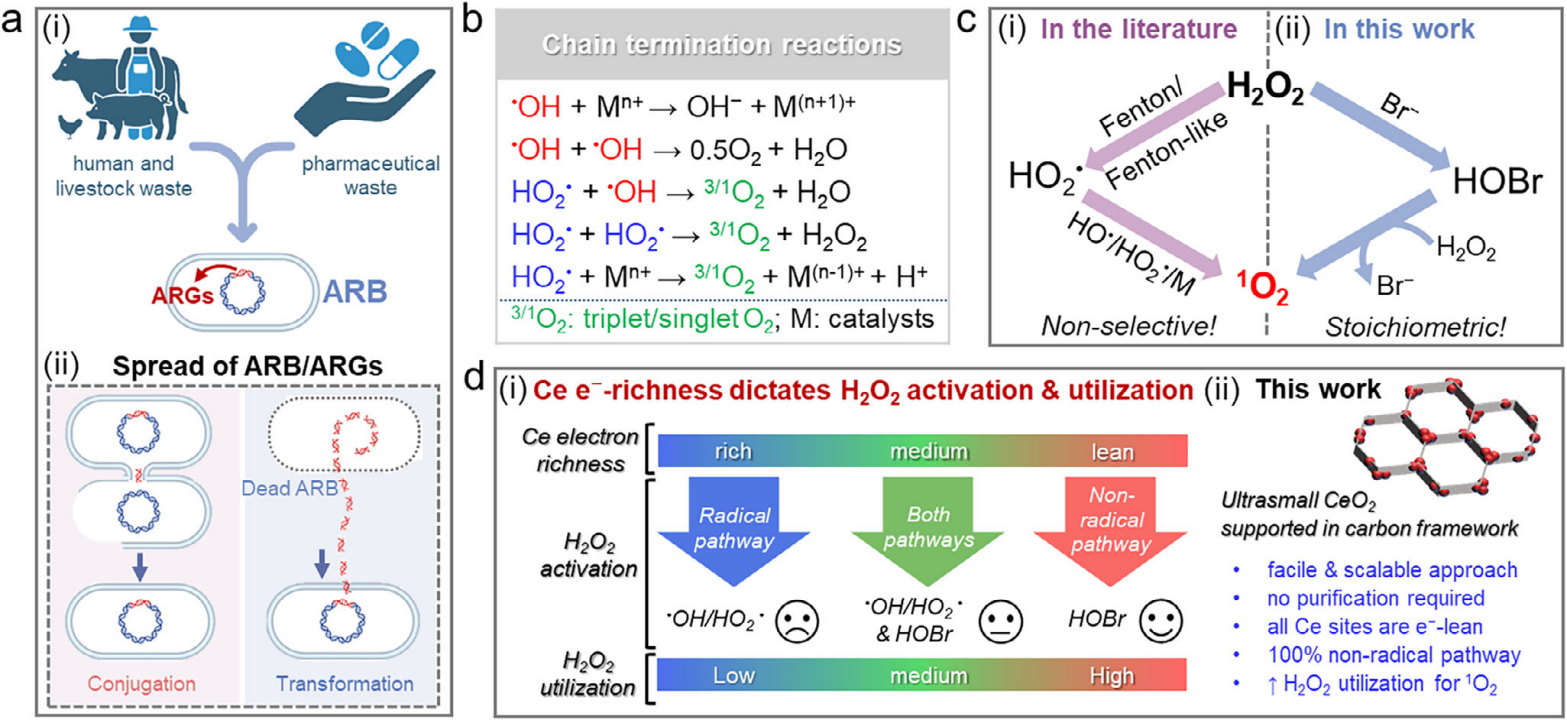
(a) Schematic illustration of (i) the accumulation and (ii) spread of ARBs and ARGs in aquatic environments. (b) Chain‐termination reactions of Fenton/Fenton‐like reactions. (c) Generation of ^1^O_2_ from (i) radical and (ii) nonradical pathways. (d) (i) Literature correlation of Ce electron richness with H_2_O_2_ activation pathway and utilization efficiency; (ii) advantages of our catalysts.

Among various water treatment methods, advanced oxidation processes (AOPs) have garnered significant attention over the past decade [6, 7, 8]. Considerable efforts have focused on developing Fenton/Fenton‐like catalysts to convert H_2_O_2_ into reactive oxygen species (ROS), particularly OH radicals, which exhibit redox potentials of up to 2.7 V_NHE_ [9, 10]. While many of these catalysts have proven effective in AOPs, OH radicals have a short lifetime (< 1 µs) and are rapidly scavenged not only by H_2_O_2_ itself but also by environmental background matrices (e.g., Cl^−^, Br^−^, and NO_3_^−^, etc.) and by chain‐termination reactions (Scheme 1b) [10], leading to poor H_2_O_2_ utilization for contaminant removal. Given this, nonradical ROS like singlet oxygen (^1^O_2_) have recently attracted increasing interest due to their longer lifetime (>80 µs), superior selectivity, and broader pH applicability, despite having a lower redox potential of 2.2 V_NHE_ [11]. However, the current strategy for ^1^O_2_ production from H_2_O_2_ in the literature relies on chain‐termination reactions of HO_2_ radicals (Scheme 1b)—specifically, their interactions with other OH radicals, HO_2_ radicals, or catalysts (denoted as M) (Scheme 1c‐i) [12, 13, 14, 15, 16, 17]. A recent study further reveals that the amount of ^1^O_2_ is intrinsically restricted by its equilibrium with ^3^O_2_, as determined by Marcus energy curves [18]. These limitations, together with the short radical lifetime, result in poor H_2_O_2_‐to‐^1^O_2_ conversion using this approach. Despite recent success in achieving high peroxymonosulfate (PMS)‐to‐^1^O_2_ conversion with single‐atom catalysts via nonradical pathways [19, 20, 21, 22, 23, 24, 25], H_2_O_2_ is a more environmentally friendly and affordable option (0.11$/g) compared to PMS (0.76$/g from Sigma–Aldrich), with existing use in water treatment plants. Moreover, the preparation of single‐atom catalysts requires multiple synthetic and purification steps, as well as extensive characterization, significantly limiting their practical application. Therefore, developing simple, scalable catalysts that do not require labor‐intensive purification and can facilitate nonradical H_2_O_2_‐to‐^1^O_2_ conversion is highly desirable for combating ARB and ARGs.

In nature, marine algae utilize bromoperoxidases (BPOs) to combat biofouling by converting H_2_O_2_ and Br^−^ in seawater into HOBr **(reaction (i))**, which is reported to decompose cell membranes [26, 27] and interfere with bacterial communication [28]. Among the materials known to catalyze this reaction [29], ceria (CeO_2_) stands out for its versatility [30, 31] and superior activity in producing HOBr for anti‐bacterial/fouling applications [32, 33, 34, 35, 36, 37, 38, 39, 40, 41, 42]. However, less emphasized in these reports is the fact that the produced HOBr can further react with additional H_2_O_2_ to exclusively generate ^1^O_2_ even in the absence of catalysts **(reaction (ii))** [43, 44, 45], with Br^−^ serving as a co‐catalyst in the overall catalytic cycle **(reaction (iii))**. Unlike HO_2_ radical‐mediated ^1^O_2_ production reported in the literature (Scheme 1c‐i), this process bypasses equilibrium limitations with ^3^O_2_ and proceeds via a nonradical, HOBr‐mediated pathway (Scheme 1c‐ii), in which the stoichiometric H_2_O_2_‐to‐^1^O_2_ conversion (2:1) theoretically achieves a maximum efficiency of 50%. However, to ensure that H_2_O_2_ is predominantly activated via this pathway in a catalytic system, the electronic state of the catalyst's active sites must be optimized [46, 47, 48]. As illustrated in Scheme 1d‐i, recent studies [49, 50] have concluded that electron‐lean Ce sites activate H_2_O_2_ via the non‐radical pathway, forming peroxo species for HOBr production, while electron‐rich Ce sites tend to convert H_2_O_2_ to OH/HO_2_ radicals through the radical pathway. Since OH/HO_2_ radicals do not yield HOBr when reacting with Br ^−^ [51] and instead decompose H_2_O_2_, an ideal CeO_2_ catalyst thus requires electron‐lean Ce sites to favor nonradical H_2_O_2_ activation over the radical pathway. However, typical CeO_2_ catalysts without surface control often have Ce sites of medium electron density that activate H_2_O_2_ via both pathways [49, 50, 52], leading to poor H_2_O_2_ utilization and reduced performance.

1. HOBr production: H_2_O_2_ + Br^−^ + H^+^ → HOBr + H_2_O
2. ^1^O_2_ production: HOBr + H_2_O_2_ → H^+^ + H_2_O + Br^−^ + ^1^O_2_
3. Overall reaction: 2H_2_O_2_ → 2H_2_O + ^1^O_2_

Herein, we present a straightforward, scalable method to prepare ultrasmall CeO_2_ NPs supported on a carbon framework by air‐calcining a mixture of Ce(NO_3_)_3_ and PVP (Scheme 1d‐ii). The resulting product can be readily used without the need for purification. Additionally, all Ce sites were tuned to have sufficiently low electron density, ensuring H_2_O_2_ activation along a nonradical pathway that closely mimics BPOs for HOBr generation. The optimized sample even outperforms this natural enzyme in converting HOBr with H_2_O_2_ to yield ^1^O_2_. Together with its greater cost‐effectiveness and thermal/pH stability, this renders our approach more practical for combating antibiotic resistance (cf. BPOs). For comparison, we also prepared an electron‐rich counterpart with N‐bonded Ce sites that activates H_2_O_2_ exclusively via the radical pathway. The results demonstrate that bio‐inspired nonradical H_2_O_2_ activation is superior to the typical Fenton/Fenton‐like radical pathway not only in dye removal across pH levels and common environmental ions, but also in the degradation of antibiotics, ARB, and their associated ARGs.

## 2 Results and Discussion

### 2.1 Preparation and Characterization

The synthetic process is straightforward: calcining a pre‐mixed Ce(NO_3_)_3_ and PVP at elevated temperatures in air (Figure 1a). The resulting sample, referred to as Air‐X (where X represents the calcination temperature), can yield more than 50 g per run using our laboratory oven (Figure S1). Scanning electron microscopy (SEM) images show that porous frameworks begin to form in samples obtained at temperatures above 350°C (Figure 1b and Figure S2). However, the framework structures were found to collapse into broken fragments at temperatures exceeding 750°C. The transmission electron microscopy (TEM) images reveal no formation of nanoparticles (NPs) in Air‐150 and Air‐250. In contrast, samples prepared at higher temperatures exhibit a high density of NPs dispersed within the framework, with sizes gradually increasing from 3.05 nm for Air‐350 to 8.23 nm for Air‐650 (see Figure S3 for size statistics). The significant increase in particle size for Air‐750, reaching 22.53 nm, is likely due to the decomposition of the framework. The sharpening of the fluorite diffraction peaks (PDF #43‐1002) in their X‐ray diffraction (XRD) patterns (Figure 1c) confirms that these are CeO_2_ NPs, with particle sizes matching the trend derived from their (111) peak using the Scherrer equation (Figure 1d). The crystalline nature of these CeO_2_ NPs is further supported by High‐resolution TEM (HRTEM), which shows a subsurface lattice spacing of 0.32 nm, corresponding to the (111) surface of CeO_2_ (Figure S4).

**FIGURE 1:**
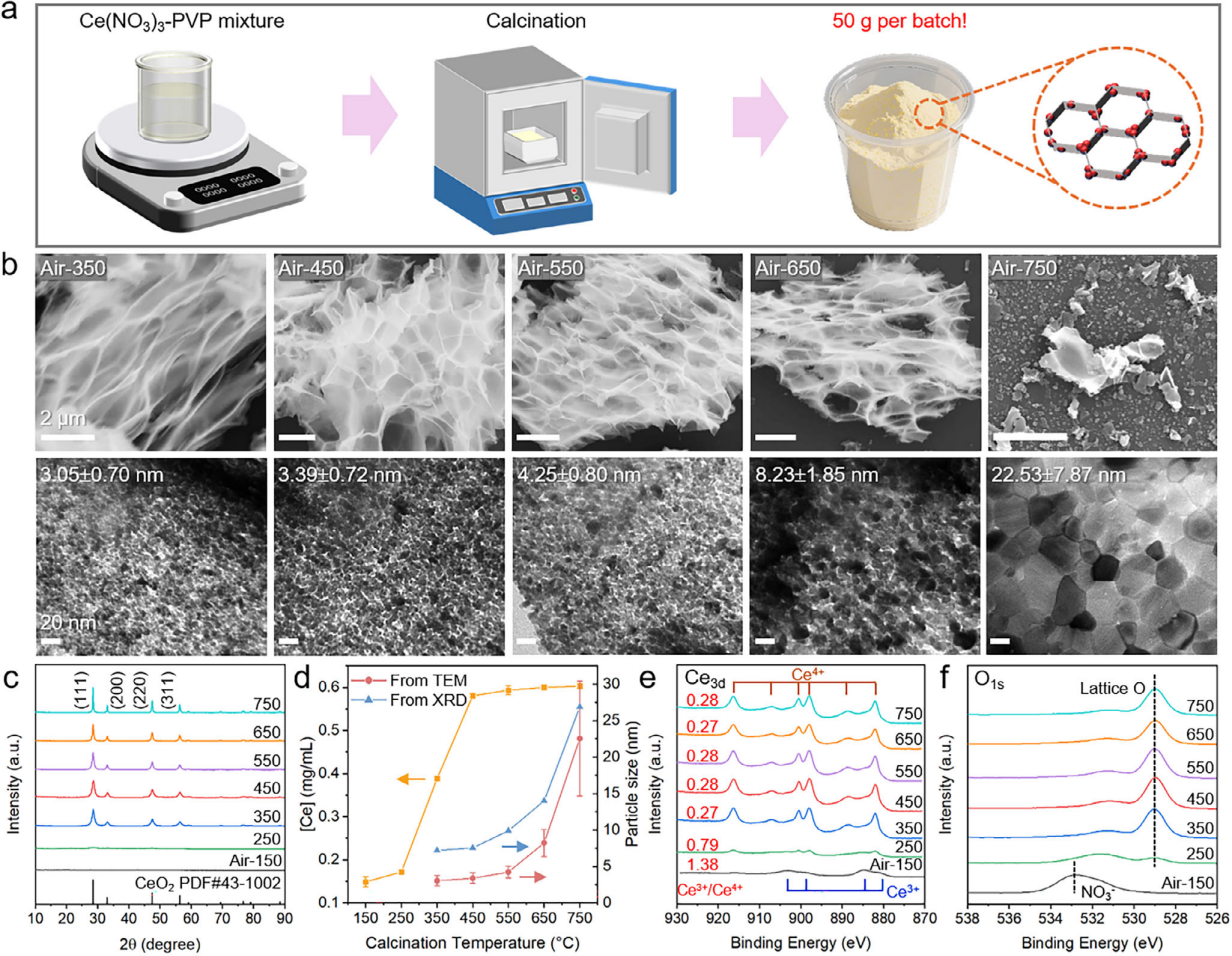
(a) Schematic illustration of the synthesis of carbon‐based frameworks populated with CeO_2_ NPs. (b) SEM (top) and TEM (bottom) images of samples (all scale bars represent 2 µm for SEM and 20 nm for TEM images) and their corresponding (c) XRD patterns. (d) Correlation of Ce concentration and particle size of samples with their synthesis temperature. XPS analysis of samples in the regions of (e) Ce_3d_ and (f) O_1s_.

Ce_3d_ X‐ray photoelectron spectroscopy (XPS) reveals a transition from ionic Ce^3+^ in the precursor of Air‐150 to lattice Ce^4+^ in the CeO_2_ NPs of samples obtained at temperatures above 350°C (Figure 1e). This also results in a decrease in the Ce^3+^/Ce^4+^ratios, dropping from 1.38 for Air‐150 to 0.79 for Air‐250 and stabilizing around 0.28 for the remaining samples (see Figure S5 for peak deconvolution). Such a transition is also evident in the corresponding XPS O_1s_ spectra (Figure 1f), which demonstrates a shift of oxygen from NO_3_^−^ in Air‐150 to lattice oxygen in the CeO_2_ for samples synthesized above 350°C. In contrast, both XPS N_1s_ and C_1s_ signals exhibit no shifts but show reduced peak intensity at elevated temperatures. Specifically, the signals for NO_3_^−^ and pyrrolic N from PVP are barely detectable in samples obtained above 350°C (Figure S6a), which may correlate with the significant mass loss observed around 300°C in thermogravimetric analysis (TGA) (Figure S7). Meanwhile, the XPS C_1s_ signal gradually decreases with rising temperatures and stabilizes for samples above 450°C (Figure S6b), where no further mass reduction is observed in the TGA. The decrease of these two elements in the samples obtained at elevated temperatures can also be visualized in the SEM elemental mapping (Figure S8), attributed to thermal decomposition of the Ce(NO_3_)_3_ and PVP mixture into carbon/nitrogen‐based gaseous products. The concentration of Ce (denoted as [Ce]) thus increases sharply from Air‐150 to Air‐350 and is maximized in samples obtained above 450°C (Figure 1d), as determined by inductively coupled plasma optical emission spectrometry (ICP‐OES; see Table S1 for details). Overall, our approach allows for the large‐scale production of CeO_2_ NPs as small as 3 nm, densely dispersed within a carbon‐based framework, in an exceptionally simple manner.

### 2.2 HOBr and ^1^O_2_ Production

The catalytic HOBr production from H_2_O_2_ and Br^−^ over these samples (w/o purification) was assessed through the bromination of phenol red (PR) to Br_4_PR, which has a characteristic absorbance at 590 nm (see Figure S9 for raw spectra). No activity was observed for Air‐150 and Air‐250 during the testing period (Figure 2a‐i), suggesting that neither ionic Ce^3+^ nor C/N species in the framework are active in the reaction. This, along with the observed HOBr production from Air‐350/450/550/650, confirms that CeO_2_ is the key material catalyzing this reaction. Although CeO_2_ NPs were also formed in Air‐750, their large particle size significantly hindered their catalytic activity (Figure S9). Air‐350, which contains the smallest CeO_2_ NPs, exhibits lower HOBr activity than Air‐450 and Air‐550, as also indicated by their corresponding rate constants (Figure 2a‐ii; see kinetic fittings in Figure S10). We attribute this to the low [Ce] in Air‐350, which is ca. two‐thirds that of other CeO_2_‐containing samples (Figure 1d). This is further supported by the comparable or even slightly higher activities observed for Air‐350 in HOBr production (and other reactions, cf. Air‐450) when tested with equivalent Ce loadings (Figure S11). As Air‐450/550/650 have similar [Ce] levels, the reduced HOBr production at higher temperatures can be primarily attributed to their increasing CeO_2_ particle size. Since HOBr further reacts with H_2_O_2_ to yield ^1^O_2_, we also monitored ^1^O_2_ production across the samples at equal catalyst weight using electron paramagnetic resonance (EPR) with 2,2,6,6‐tetramethylpiperidine (TEMP) as the probe. The presence of ^1^O_2_ can specifically oxidize TEMP to the nitroxide radical 2,2,6,6‐tetramethylpiperidine‐N‐oxide (TEMPO), resulting in a 1:1:1 triplet EPR signal. As shown in Figure 2b, this signal is observed exclusively in CeO_2_‐containing samples, exhibiting intensities in the order: Air‐450> Air‐550> Air‐350> Air‐650, which aligns well with their trends in HOBr production. Surprisingly, no OH radicals were detected in these samples (Figure S12a), as evidenced by the absence of the characteristic 1:2:2:1 EPR signal resulting from the oxidation of 5,5‐dimethyl‐2‐pyrrolidone‐N‐oxyl (DMPO) to DMPO‐OH. This was further confirmed by time‐dependent EPR investigations on Air‐450 throughout the testing period (Figure S12b). Additionally, negligible HO_2_ radicals were detected for Air‐450, both in the presence and absence of Br^−^ (Figure S13), suggesting that the observed ^1^O_2_ production does not arise from typical chain‐termination reactions of HO_2_ radicals (Scheme 1b). To verify that the observed ^1^O_2_ originates from HOBr, we used singlet oxygen sensor green (SOSG), a selective ^1^O_2_ probe, in the absence/presence of PR (as a HOBr quencher). As shown in Figure S14, Air‐450 exhibits strong SOSG fluorescence at 530 nm, indicating ^1^O_2_ production. The significant reduction of this signal upon PR addition confirms that the observed ^1^O_2_ indeed arises from HOBr via **reaction (ii)** [HOBr + H_2_O_2_ → H^+^ + H_2_O + Br^−^ + ^1^O_2_]. These findings confirm that all Ce sites in the samples prepared by our approach are sufficiently electron‐lean to suppress H_2_O_2_ activation via the radical pathway while promoting the nonradical pathway, thereby enabling Br^−^‐mediated ^1^O_2_ production analogous to that of BPOs.

**FIGURE 2:**
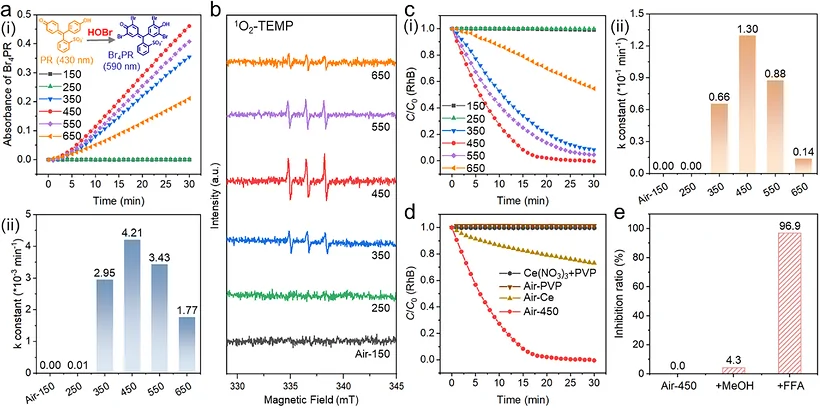
(a) (i) Time‐dependent production of Br_4_PR monitored at 590 nm over samples and (ii) their *k* constant. (b) EPR spectra for the detection of ^1^O_2_. (c) (i) Time‐dependent RhB degradation over samples and (ii) their *k* constant. (d) Comparison of RhB degradation over samples and (e) the inhibition experiments in the presence of ROS scavengers.

The oxidative power of the system (catalysts + H_2_O_2_ + Br^−^) was further assessed through the degradation of rhodamine B (RhB), a model industrial printing contaminant. Figure 2c shows their time‐dependent results along with the corresponding rate constants (see Figure S15 for raw UV–vis spectra and Figure S16 for kinetic fitting). The degradation efficiency of RhB for these samples was found to follow the order: Air‐450> Air‐550> Air‐350> Air‐650, which positively correlates with their yield of HOBr (Figure 2a) and ^1^O_2_ (Figure 2b). In contrast to the negligible activity of Air‐150/250, Air‐450 achieves over 90% removal of RhB within 15 min. We attribute the high activity of this sample to its optimal [Ce] and the ultrasmall CeO_2_ NPs densely dispersed within the porous framework, resulting from the air calcination of pre‐mixed Ce(NO_3_)_3_ and PVP at 450°C. This is evident from the negligible and low activities of the control samples obtained by air‐calcining PVP (Air‐PVP) and Ce(NO_3_)_3_ (Air‐Ce) alone at 450°C (Figure 2d). Neither of them exhibited a porous framework (Figure S17), with CeO_2_ NPs primarily present in aggregated form in Air‐Ce. While catalytically inert itself, the carbon framework is believed to act not only as a support to disperse ultrasmall CeO_2_ NPs but also to provide a porous structure for efficient mass transfer. Notably, the RhB removal efficiency of Air‐450 is consistent across batches, including those subjected to additional purification (Figure S18), which is unsurprising because unreacted ionic Ce^3+^, PVP, and its derivatives are inactive in the reaction. This finding demonstrates Air‐450's reproducibility and indicates that labor‐intensive purification is unnecessary, facilitating its practical applications.

To identify the key ROS responsible for contaminant degradation in Air‐450, quenching experiments were conducted using methanol and furfuryl alcohol (FFA) to scavenge OH radicals and ^1^O_2_, respectively (Figure 2e). As expected, since no OH radicals were produced by the sample (Figure S12a), methanol had a negligible inhibitory effect on RhB removal. In contrast, the presence of FFA inhibited RhB degradation by nearly 97%, significantly reducing the rate constant from 0.13 to 0.004 min^−1^ (Figure S19). These results suggest that while both HOBr and ^1^O_2_ are produced in the system, ^1^O_2_ is the key species responsible for degrading RhB, likely due to its higher redox potential of 2.2 V_NHE_ compared to HOBr's 1.3 V_NHE_ [53]. To further support this finding, we analyzed the RhB degradation products for Air‐450 by HPLC‐MS (Figure S20). The results show that negligible oxidative bromination of RhB or its degradation intermediates occurred during the reaction. Notably, under our reaction conditions, the formation of BrO_3_^−^, a Group 2B probable human carcinogen, was below the detection limit of ion chromatography (0.002 mg/L, Figure S21a). This negligible formation is attributed to the mild nonradical pathway employed herein, which avoids the excessive oxidation inherent to typical O_3_/OH radical‐based systems (Figure S21b).

The pH had a minimal effect on RhB removal, with comparable performance observed across the pH range of 4 to 9 (Figure S22a). This is distinct from the uncatalyzed HOBr‐to‐^1^O_2_ conversion **(reaction (ii))**, in which the ^1^O_2_ yield is theoretically expected to peak at pH ≈10 [54], with lower yields at more acidic pH values (see discussion in Figure S22b). The origin of this discrepancy in pH dependence is still under investigation. However, it is believed to be closely associated with the CeO_2_ (111) surface, which promotes the dissociative adsorption of HOBr. Among common ions, bicarbonate, carbonate, and phosphate (1 mM each) were found to significantly reduce RhB removal over Air‐450, likely by forming surface complexes on metal oxides that block active sites (Figure S23a). Since our catalytic system is designed for tertiary treatment, standard pre‐treatment (e.g., coagulation with trace Al^3+^/Fe^3+^ salts) can remove these anions before advanced oxidation, thereby minimizing interference and preserving CeO_2_ activity, with negligible inhibition at 5 µM (Figure S23b). Since most natural organic matter (NOM) is removed during primary and secondary biological treatments, with residual typically at ∼4 mg/L dissolved organic carbon (DOC) [55], we tested 0.1 mM humic acid (∼6 mg/L DOC) and found negligible interference with RhB removal over Air‐450 (Figure S23b). Collectively, as a catalyst designed for tertiary‐treatment systems, its performance is largely unaffected by pH and common ions.

### 2.3 Influence of Calcination Atmosphere on H_2_O_2_ Activation Pathways

It has been reported that the surface electronic state of CeO_2_ NPs could be altered by the calcination atmosphere during preparation, which may influence their catalytic activity [56, 57]. Since Air‐450 outperformed other CeO_2_‐containing samples in both HOBr/^1^O_2_ yield and RhB degradation, we evaluate this effect by calcining the pre‐mixed Ce(NO_3_)_3_ and PVP at 450°C in a nitrogen atmosphere (denoted as N_2_‐450) for comparison. In general, N_2_‐450 is structurally similar to Air‐450. As shown in Figure 3a, this sample also contains ultrasmall NPs (about 3.0 nm in size; see statistics in Figure S24) densely supported in a porous carbon framework. The CeO_2_ composition and crystalline nature of these NPs were verified by the fluorite XRD pattern and high‐resolution TEM, respectively (Figure S25). Similar results were also obtained for N_2_‐450 in the XPS O_1s_ and Ce_3d_ spectra (Figure S26), as well as the Ce^3+^/Ce^4+^ ratios (cf. Air‐450). The key difference found between Air‐450 and N_2_‐450 is that the latter retains some pyrrolic N from PVP and exhibits a new Ce‐N signal at a lower energy of 398 eV (Figure 3b) [58]. Since these Ce‐N species were also observed in the sample prepared in argon (Figure S27), the source of nitrogen is thus not from the N_2_ used, but from the preserved pyrrolic PVP nitrogen under an inert atmosphere. Given that nitrogen has a lower electronegativity than oxygen, N‐bonded Ce sites in N_2_‐450 are expected to have higher electron density (or lower Lewis acidity) compared to Ce sites coordinated solely by oxygen in Air‐450. To distinguish their Ce sites, we employed probe‐assisted ^31^P NMR [59, 60, 61, 62] using trimethylphosphine oxide (TMPO) as a surface probe. This Lewis base molecule can coordinate with surface Ce sites, forming TMPO‐Ce adducts, where stronger Ce Lewis acidity results in a shift of the ^31^P resonance (δ^31^P) to higher ppm values. As expected, Air‐450 displays a δ^31^P signal at 53.4 ppm, while N_2_‐450 shows a downshift of 5.5 to 47.9 ppm (Figure 3c). This confirms that Ce sites in N_2_‐450 indeed exhibit higher electron density than those in Air‐450 due to the formation of N‐bonded Ce sites. One may wonder why XPS shows the same spectra and Ce^3+^/Ce^4+^ ratio while TMPO‐^31^P NMR reveals that surface Ce sites bear distinct electronic states (or Lewis acidity). This is because conventional XPS with Al Kα X‐rays (1486.6 eV) probes Ce photoelectrons from depths of up to 7 nm [63], meaning it is not surface‐sensitive and thus dilutes signals from the topmost surface. Moreover, the discrete oxidation states (3+ or 4+) cannot reflect the continuous variation in Ce sites with distinct coordination/electronic structures [61, 62].

**FIGURE 3:**
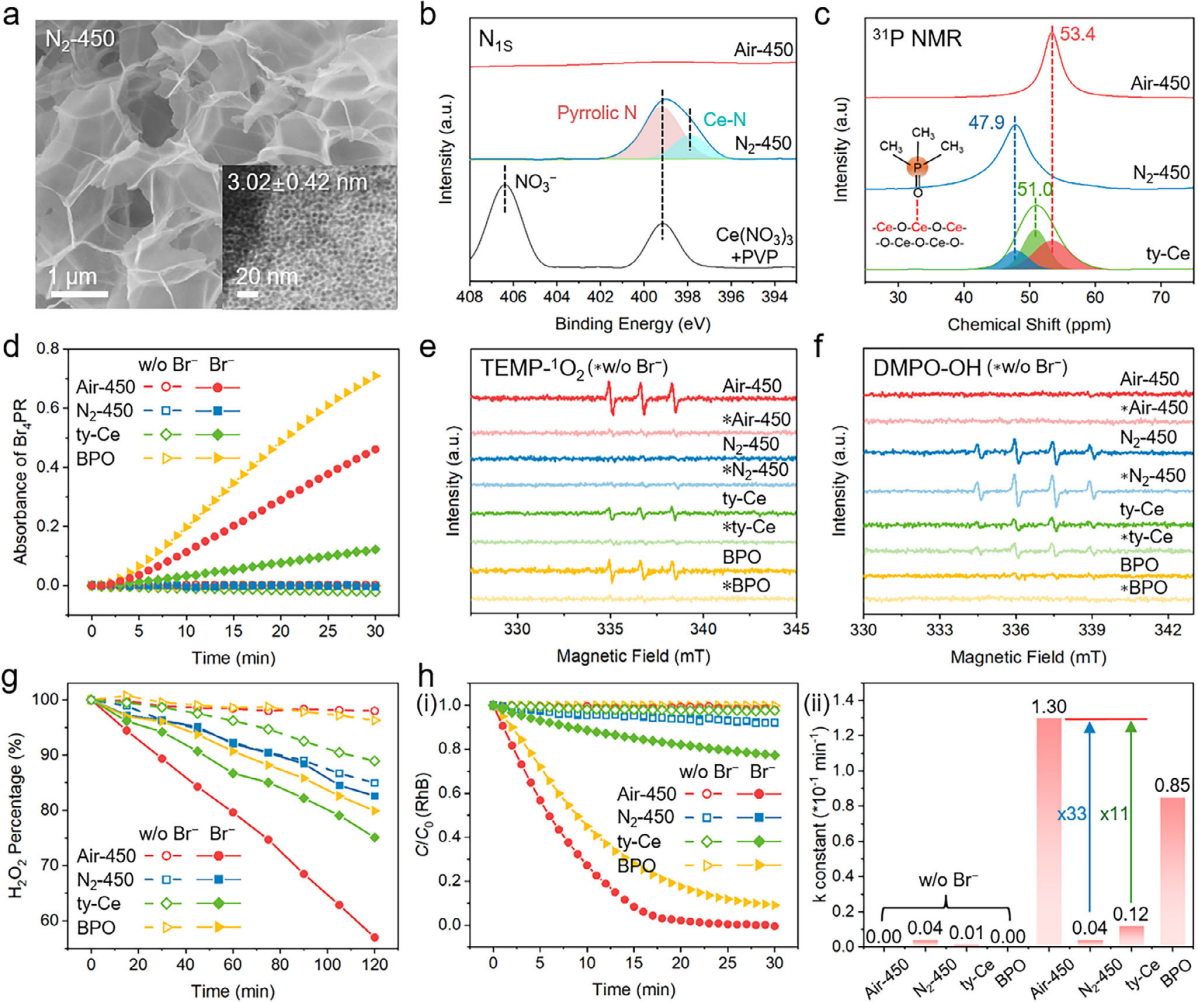
(a) SEM and TEM images of N_2_‐450. (b) XPS N_1s_ spectra and (c) TMPO‐assisted ^31^P NMR spectra of the samples. (d) Time‐dependent production of Br_4_PR monitored at 590 nm and EPR monitoring of the generation of (e) ^1^O_2_ and (f) OH radicals for the samples. Time‐dependent (g) H_2_O_2_ decomposition and (h) (i) RhB degradation and (ii) their corresponding rate constants for the samples in the absence/presence of Br^−^.

Distinct from Air‐450, which produces HOBr (Figure 3d) and ^1^O_2_ (Figure 3e) upon Br^−^ addition via the nonradical pathway, N_2_‐450 showed none of these activities, regardless of Br^−^ presence. In contrast, it exclusively activates H_2_O_2_ via the radical pathway, producing OH radicals (probed by DMPO, Figure 3f), as evidenced by the time‐dependent evolution of the characteristic 1:2:2:1 EPR signal throughout the testing period (Figure S12b). This divergent H_2_O_2_ activation pathway was further validated by fluorescence assays (see Figure S28 for details). The exclusive production of OH radicals also accounts for the observed HO_2_ radicals in N_2_‐450, but not in Air‐450, via the reaction with H_2_O_2_ [•HO + H_2_O_2_ → H_2_O + •HO_2_] (Figure S13). However, these HO_2_ radicals did not result in a detectable ^1^O_2_ signal for N_2_‐450 (Figure 3e), likely due to interference from other chain‐termination reactions and the intrinsic equilibrium with ^3^O_2_, as discussed in the introduction. The opposing H_2_O_2_ activation behavior of N_2_‐450 (cf. Air‐450) is believed to arise from its N‐bonded Ce sites, which have sufficiently high electron density to activate H_2_O_2_ via the radical pathway, thereby suppressing the nonradical pathway for HOBr/^1^O_2_ production. This is supported by the sample synthesized in argon, which also contains Ce‐N species (Figure S27) and exclusively catalyzes the conversion of H_2_O_2_ to OH radicals rather than ^1^O_2_ (Figure S29). The ∼100% control of H_2_O_2_ activation observed over Air‐450 and N_2_‐450, via either nonradical or radical pathways, can thus be attributed to their separated δ^31^P signals, which show minimal overlap (Figure 3c). To further confirm this, a typical CeO_2_ NPs (denoted as ty‐Ce) with comparable sizes and similar structural properties was prepared (Figure S30). The TMPO‐^31^P NMR result for ty‐Ce exhibits a δ^31^P signal between those of Air‐450 and N_2_‐450 (Figure 3c), with three deconvoluted peaks at 53.4, 51.0, and 47.9 ppm, suggesting the presence of surface Ce sites with at least three distinct electron densities. As expected, these variations in ty‐Ce enable H_2_O_2_ activation via both the nonradical pathway for HOBr (Figure 3d)/^1^O_2_ (Figure 3e) production, as well as the radical pathway for OH (Figure 3f) and HO_2_ (Figure S13) radicals observed in the sample. However, because the two pathways compete for H_2_O_2_, ty‐Ce performs much worse than Air‐450 and N_2_‐450 in their respective reactions.

Since OH radicals produced through the radical pathway decompose free H_2_O_2_, while the peroxo species formed via the nonradical pathway only consume H_2_O_2_ in the presence of Br^−^, one should observe a distinct change in H_2_O_2_ concentration ([H_2_O_2_]) for these samples over time (Figure 3g). Indeed, N_2_‐450, which activates H_2_O_2_ through the radical pathway, displays a gradual decline in [H_2_O_2_] regardless of Br^−^ presence. In contrast, no decomposition of H_2_O_2_ is observed for Air‐450 without Br^−^, confirming that the surface peroxo species do not react with free H_2_O_2_. However, in the presence of Br^−^, HOBr/^1^O_2_ formation consumes H_2_O_2_, leading to a sharp decrease in [H_2_O_2_] for this sample. The much slower [H_2_O_2_] decrease observed for N_2_‐450 is likely due to competitive removal of OH radicals by other chain‐termination reactions (Scheme 1b). As for ty‐Ce, which activates H_2_O_2_ via both pathways, it decomposes H_2_O_2_ without Br^−^ like N_2_‐450, while the rate of [H_2_O_2_] decomposition is further accelerated upon adding Br^−^, similar to Air‐450. The above understanding of H_2_O_2_ activation with/without Br^−^ accounts for the RhB removal performance of the samples (Figure 3h‐i), with Air‐450 showing a rate constant (1.3 × 10^−1^ min^−1^) that is ∼11‐ and ∼33‐fold higher than those of ty‐Ce (0.12) and N_2_‐450 (0.04), respectively (Figure 3h‐ii). Since these samples do not adsorb RhB (Figure S31), the contribution of adsorbed RhB to their observed activities can be excluded. See Figure S32 for the total organic carbon (TOC) analysis of their supernatant after the reaction. The limited activity of N_2_‐450, regardless of Br^−^ presence, is attributed to the short‐lived nature of OH radicals, which are rapidly consumed by chain‐termination pathways (Scheme 1b) and unproductive H_2_O_2_ decomposition (Figure 3g). In contrast, Air‐450 enables nonradical Br^−^‐mediated H_2_O_2_ conversion to longer‐lived, more selective ^1^O_2_, thereby bypassing these disadvantages. As for ty‐Ce, which facilitates H_2_O_2_ activation via both pathways, it displays intermediate activity between the two as expected. The H_2_O_2_ utilization efficiency of Air‐450 and N_2_‐450 was further assessed (see Figure S33 for details). Air‐450, which exclusively follows the nonradical pathway, yielded an H_2_O_2_‐to‐^1^O_2_ conversion efficiency of ∼10%. The deviation from the theoretical 50% (2:1 stoichiometry, **reaction (iii)**) is attributed to incomplete HOBr‐to‐^1^O_2_ conversion, incomplete ^1^O_2_ trapping, and adsorption of H_2_O_2_ onto the catalyst surface without being consumed. In contrast, N_2_‐450, which exclusively follows the radical pathway, showed a much lower efficiency of ∼3.2%, due to inherent scavenging by the various species discussed above.

Altogether, these findings highlight the necessity for all Ce sites in a given CeO_2_ catalyst to have sufficiently low electron density, enabling exclusive H_2_O_2_ activation via the nonradical pathway for peroxo species, thereby providing a higher H_2_O_2_ utilization in BPO‐inspired ^1^O_2_ production for contaminant degradation.

### 2.4 Comparison With BPOs and Kinetic Studies

In general, the catalytic behavior of Air‐450 closely resembles that of the natural enzyme BPOs. Both samples activate H_2_O_2_ via a nonradical pathway, yielding HOBr (Figure 3d) and ^1^O_2_ (Figure 3e) while suppressing OH (Figure 3f) and HO_2_ (Figure S13) radical production. H_2_O_2_ is also consumed only in the presence of Br^−^ (Figure 3g) for RhB degradation (Figure 3h). Surprisingly, Air‐450 further catalyzes the reaction between HOBr and H_2_O_2_ to produce ^1^O_2_, as evidenced by its lower HOBr level but higher ^1^O_2_ yield compared to BPOs. This explains the greater H_2_O_2_ consumption and improved RhB removal observed for Air‐450, supporting again the conclusion that ^1^O_2_ (rather than HOBr) is the key species responsible for degrading RhB. Additionally, Air‐450 outperformed BPO in both cost‐effectiveness and pH/thermal stability. By considering raw‐material quantities, conversion efficiencies, and electricity expenses, the production cost per kilogram was estimated (Table S2). As shown in Figure 4a, production costs for CeO_2_‐containing samples range from $160 to $300 per kilogram. This is over a million times lower than BPO (∼$5 billion per kilogram), which requires a specific natural seaweed and complex extraction/purification processes (Figure S34). Cost‐effectiveness was further assessed by normalizing RhB‐degradation rate constants to production costs (Figure 4a). Air‐450 demonstrated the highest cost‐effectiveness among the samples and, crucially, relative to BPO, indicating greater economic viability for large‐scale production. For pH and thermal stability tests, Air‐450 and BPO were pretreated in aqueous solutions at various pH values and temperatures before being tested for RhB degradation (see the experimental section for details). As shown in Figure 4b‐i, BPO retains RhB‐degradation activity only within a pH range of 7–8. It tolerates temperatures up to 45°C (Figure 4b‐ii), with activity dropping sharply to < 30% at 65°C. These pH and thermal instabilities of BPO can be attributed to protein denaturation. In contrast, Air‐450 remained stable across the tested conditions owing to its inorganic nature.

**FIGURE 4:**
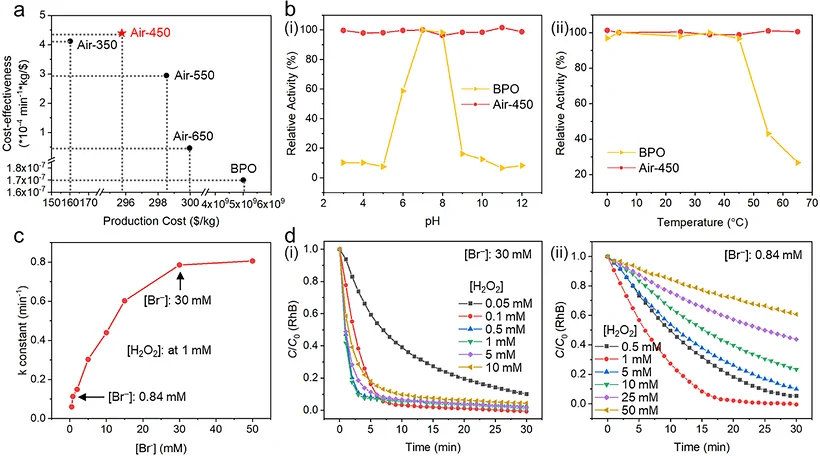
(a) Estimated production costs and cost‐effectiveness of the samples. (b) The (i) pH and (ii) temperature stability of Air‐450 and BPO. Activities at pH 7 and 4°C were set to 100%, respectively. (c) Dependence of RhB removal rate on [Br^−^] with [H_2_O_2_] fixed at 1 mM. (d) Degradation of RhB at different [H_2_O_2_] with [Br^−^] fixed at (i) 30 mM and (ii) 0.84 mM.

To explore the effects of H_2_O_2_ and Br^−^ concentrations on RhB removal by Air‐450, we first fixed [H_2_O_2_] at 1 mM and evaluated degradation activity at varying Br^−^ concentrations (denoted as [Br^−^]). Figure 4c shows that the RhB removal rate increases with [Br^−^], reaching a plateau at around 30 mM (see Figure S35 for time‐dependent results). We then fixed [Br^−^] at 30 mM while varying [H_2_O_2_]. The result indicates that RhB degradation can reach ∼90% within 30 min at a very low H_2_O_2_ concentration of 0.05 mM (Figure 4d‐i). However, unlike Br^−^, the removal rate maximizes at [H_2_O_2_] = 1 mM and then gradually decreases in efficiency (Figure S36a). A similar trend is observed when [Br^−^] is fixed at 0.84 mM (Figure 4d‐ii), the concentration of Br^−^ in seawater used for in‐house flushing in some regions (e.g., Hong Kong). The RhB removal rate also increases with [H_2_O_2_] to a maximum and then falls sharply (Figure S36b). These findings suggest that co‐adsorption of H_2_O_2_ and Br^−^ is critical for HOBr/^1^O_2_ production, while H_2_O_2_ interacts more strongly with Ce sites than Br^−^.

### 2.5 Atomic Insights From Simulations

Density functional theory (DFT) calculations were performed to elucidate how nitrogen alters electronic properties of Ce sites and to explain the distinct H_2_O_2_ activation pathways observed for N_2_‐450 and Air‐450. We first considered the formation of N‐bonded Ce sites on the (111) surface by substituting lattice oxygen with nitrogen (Figure S37a); however, these configurations require formation energies (E_f_) above 5 eV, indicating they are energetically unfavorable. In contrast, introducing nitrogen as a surface adatom significantly reduces the formation energy to below 3 eV (Figure S37b). The most likely configuration, featuring nitrogen coordinated to one O atom and two Ce atoms (E_f_ = 2.37 eV), was denoted as N‐(111) for further Bader charge analysis. As shown in Figure 5a, Ce atoms coordinated with oxygen on the pristine (111) surface have a Bader charge of 9.64 |e|, which increases to 9.89 |e| for the Ce atom coordinated to nitrogen on the N‐(111) surface. Notably, the Ce atom not directly bonded to nitrogen also shows a slight charge increase to 9.67 |e|. These results support our hypothesis that N‐bonded Ce sites are more electron‐rich owing to nitrogen's lower electronegativity (cf. oxygen). Consequently, the electron‐rich Ce atoms on N‐(111) interact more weakly with TMPO, resulting in a reduced adsorption energy (E_ads_) relative to the pristine (111) surface (Figure S38). This explains the observed downshift in δ^31^P from 53.4 ppm for Air‐450 to 47.9 ppm for N_2_‐450 in TMPO‐^31^P NMR (Figure 3c). Similar to TMPO, H_2_O_2_ adsorption is weakened on Ce sites with higher electron density. The electron‐rich Ce sites on the N‐(111) surface exhibit E_ads_ of −0.67 eV (Figure 5b‐i), which is weaker than the ‐0.76 eV for their electron‐lean counterparts on the pristine (111) surface (Figure 5b‐i’). Despite this, subsequent production of OH radicals is thermodynamically favorable on N‐(111) (E_ads_ = ‐1.11, Figure 5b‐ii) but unfavorable on the pristine (111) (E_ads_ = +0.21 eV, Figure 5b‐ii’). These findings suggest that H_2_O_2_ is more likely to be reduced at Ce sites on N‐(111) than on the pristine surface, leading to the production of OH radicals via a radical pathway, as observed experimentally for N_2_‐450 (Figure 3f).

**FIGURE 5:**
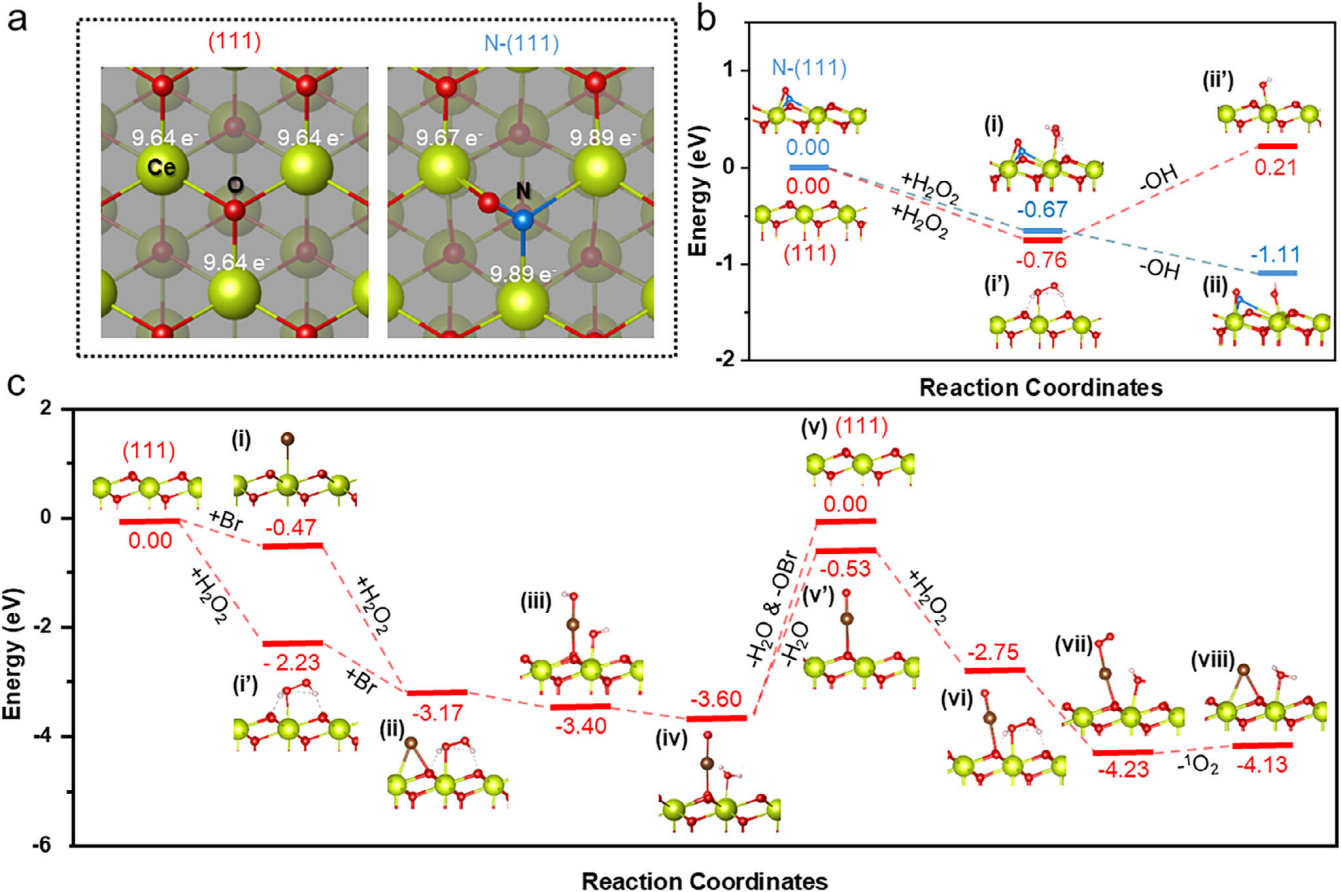
(a) Bader charge analysis of Ce sites on the pristine (111) and N‐(111) surfaces. (b) Corresponding energy profiles for the formation of peroxo species and subsequent OH release. (c) Energy profile for the conversion of two H_2_O_2_ molecules into ^1^O_2_ on the pristine (111) surface using Br as a co‐catalyst.

To investigate the nonradical H_2_O_2_ activation pathway observed exclusively for Air‐450, we further examined the reaction between the peroxo species and Br on the pristine (111) surface (Figure 5c). Because Figure 5b,c describe different reaction scenarios, their energy references were defined differently. In Figure 5b, which focuses on direct H_2_O_2_ activation, energies are referenced to an intact H_2_O_2_ molecule, while Figure 5c describes a multi‐step catalytic cycle involving bond breaking/formation and sequential oxidation of the Br co‐catalyst. To obtain a continuous relative energy profile for the pathway, we used generalized chemical potentials: E(H) = 1/2 E(H_2_) and E(O) = 1/2 E(O_2_). Additionally, to close the cycle involving unstable BrO_2_, the chemical potential of the Br species was referenced as E(Br) = E(BrO_2_) − E(O_2_). See the experimental section for details. As shown in Figure 5c, the adsorption of Br on the pristine (111) surface **(step i)** is much weaker than that of H_2_O_2_ **(step i’)**, consistent with the kinetic results observed for Air‐450 (Figure 4d). Since their co‐adsorption is critical for HOBr/^1^O_2_ production, H_2_O_2_ and Br were further introduced separately into the corresponding model. Surprisingly, both configurations converged to the same model containing a bridged Ce‐Br‐O_lattice_ species **(step ii)**, with an E_ads_ of −3.17 eV. A nearby peroxo species further oxidizes this species, yielding surface BrOH and an OH group **(step iii)**. After proton exchange, the first redox reaction ends with the generation of surface BrO, with the second oxygen atom of the peroxo species being reduced to water **(step iv)**. Protonation of the desorbed OBr^−^ then yields HOBr [HOBr ⇄ OBr^−^ + H^+^ (pK_a_: 8.63)] **(step v)**, as confirmed by PR bromination for Air‐450 (Figure 3d). While the isolated vanadium site in BPOs cannot catalyze further ^1^O_2_ production with a second H_2_O_2_, experimental results show that this process is selectively promoted on Air‐450 (Figure 3e), which contains multiple nearby surface Ce sites. As also shown in Figure 5c, surface BrO may remain adsorbed on the pristine (111) surface **(step v’)** and be further oxidized by a neighboring peroxo species **(step vi)** to form surface BrO_2_ and H_2_O **(step vii)**. Subsequent release of ^1^O_2_ from BrO_2_ then regenerates bridged Ce‐Br‐O_lattice_ species **(step viii)**, which acts as a co‐catalyst in the whole process. These findings provide atomic insights into the sequential conversion of surface peroxo species to BrO and then to ^1^O_2_ on the pristine (111) surface, explaining the lower HOBr levels but higher ^1^O_2_ yield and H_2_O_2_ consumption observed for Air‐450 vs. BPOs.

### 2.6 Removal of Antibiotic, ARB, and Their ARGs

The effectiveness of N_2_‐450 and Air‐450 in removing antibiotics, ARB, and their ARGs—through the conversion of H_2_O_2_ into either OH radicals or ^1^O_2_—was examined. Ampicillin (AMP), a widely used antibiotic, was first assessed (Figure S39). Both catalysts adsorbed AMP to a comparable degree, yet only Air‐450 achieved complete degradation of free AMP within 10 min upon H_2_O_2_/Br^−^ addition, whereas N_2_‐450 showed negligible degradation, consistent with the RhB removal results. For ARB testing, since gram‐negative bacteria are more adept at developing novel resistance mechanisms due to their complex cell wall structures (cf. gram‐positive bacteria) [64], *E. coli* DH5α with the RP4 plasmid carrying the ampicillin resistance gene (amp^R^) was used as the model organism. Figure 6a,b show the bacterial viability under different conditions and the corresponding images of colonies, respectively. Compared to the *E. coli* DH5α alone (condition 1), a negligible inactivation was observed in the presence of H_2_O_2_ and Br^−^ (condition 2). Upon incubation with either N_2_‐450 (condition 3) or Air‐450 (condition 4), the viability decreased to ca. 75%. Notably, no further increase was observed for either sample with H_2_O_2_ (conditions 5 and 6). This is understandable for Air‐450, as no reactive ROS were produced. However, for N_2_‐450, which converts H_2_O_2_ into radicals, the negligible effect can be attributed to the rapid scavenging of OH radicals (lifetime < 1 µs) by background matrices and chain‐termination reactions. In the presence of both H_2_O_2_ and Br^−^, the viability of *E. coli* DH5α remains above 60% for N_2_‐450 (condition 7), while Air‐450 achieves a remarkable reduction in bacterial viability to less than 2% (condition 8). The morphology of *E. coli* DH5α under these different conditions was analyzed using scanning electron microscopy (SEM) (Figure 6c). Generally, *E. coli* DH5α maintained its morphology under conditions 1–7; however, the bacteria displayed rough and ruptured morphologies in condition 8. These findings suggest that our nonradical H_2_O_2_ activation pathway, despite producing less reactive ^1^O_2_ than OH radicals, is far more effective in antibacterial applications, due to the higher selectivity and longer lifetime. HOBr may also contribute to bacterial inactivation by brominating membrane proteins and peptidoglycan [27, 65].

**FIGURE 6:**
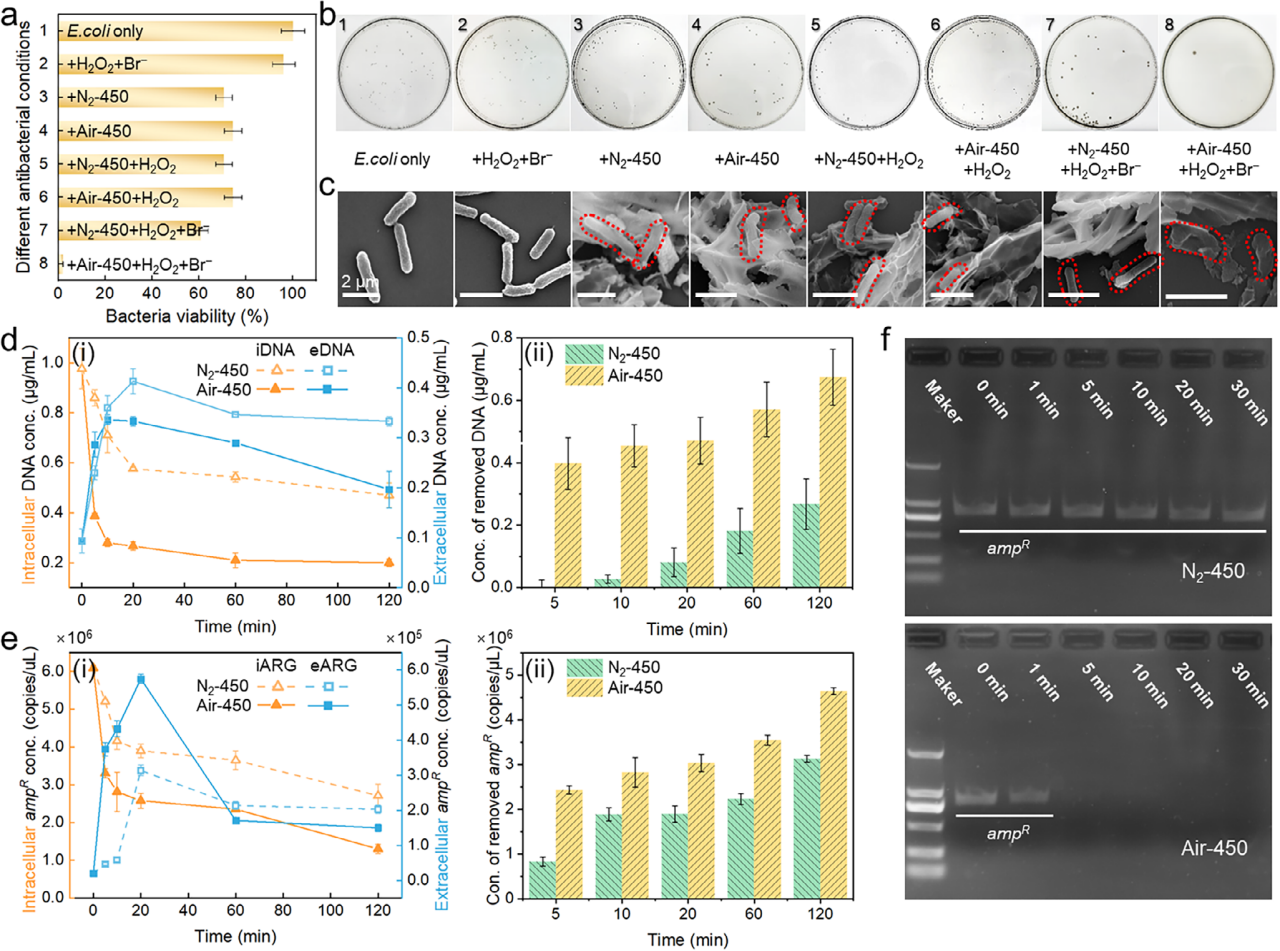
(a) Bacterial viability, (b) digital photographs, and (c) SEM images of *E. coli* DH5α under different conditions. The bacteria are highlighted by red dashed circles, and all SEM scale bars are 2 µm. The time‐dependent degradation of (d) DNA and (e) ARGs, including (i) their intracellular and extracellular concentrations and (ii) the corresponding total removal. (f) Agarose gel electrophoresis of the amp^R^ gene.

Since effective remediation requires not only removing ARB but also their ARGs, we further evaluated the time‐dependent degradation of intracellular DNA (iDNA)/ARG (iARG) and extracellular DNA (eDNA)/ARG (eARG) for N_2_‐450 and Air‐450. Notably, OH radicals and ^1^O_2_ produced by these two samples are known to induce DNA damage through distinct mechanisms: the former primarily causes strand breakage via sugar‐phosphate backbone cleavage [66], whereas the latter predominantly mediates oxidative base damage [67, 68]. As shown in Figure 6d‐i, iDNA decreases more rapidly with Air‐450, dropping below 0.3 µg/mL within 10 min, whereas N_2_‐450 remains at about 0.7 µg/mL over the same time period. The decline in iDNA for both samples is accompanied by an initial increase in eDNA (released from inactivated ARB) during the first 20 min, followed by a gradual decrease throughout the treatment period. Similar concentration variations were also observed for both intracellular and extracellular amp^R^ genes (Figure 6e‐i). iARG degradation is most pronounced within the first 10 min for both samples, accompanied by the emergence of eARG, which also peaks at 20 min; thereafter, both iARG and eARG levels decrease gradually. Overall, Air‐450 demonstrates greater total DNA (Figure 6d‐ii) and ARG (Figure 6e‐ii) removal than N_2_‐450 at all time points. Since the interference from the cell matrix may hinder ARG removal, the effectiveness of ARG degradation in the absence of these interferences was further assessed for N_2_‐450 and Air‐450 by agarose gel electrophoresis (Figure 6f). The amp^R^ band intensity remained unchanged after 30 min of treatment with N_2_‐450, similar to control experiments (Figure S40). In contrast, the amp^R^ band disappeared within 5 min under the same conditions with Air‐450. These results further confirm that this bio‐inspired nonradical H_2_O_2_ activation is superior to the typical radical pathway for combating ARB and their ARGs, as the latter is significantly limited by interference from various scavenging reactions.

### 2.7 Assessment in a Continuous Flow Reactor

Since Air‐450 can be easily produced in large quantities (> 50 g per batch, depending on the size of the oven) without purification, it holds greater potential for practical applications than most Fenton catalysts reported in the literature, which usually require multiple labor‐intensive synthesis/purification steps and yield limited amounts (typically < 100 mg per batch) [69, 70]. Accordingly, we further assessed the removal of RhB and ARB/ARG using a continuous flow reactor with Air‐450 loaded on a polyvinylidene difluoride (PVDF) membrane (Figure 7a). Approximately 6 mL of effluent was collected at specified time points for analysis. Figure 7b shows the introduction of a RhB solution mixed with H_2_O_2_ and KBr into the reactor at a flow rate of 2 mL/min through a peristaltic pump. The effluent collected from the Air‐450/PVDF membrane is nearly colorless and maintains ca. 90% removal efficiency up to 10 h (Figure 7c). In contrast, using the pristine PVDF membrane results in more than 90% RhB retention in the effluent samples at all time points. A similar trend was observed for the solution containing *E. coli* DH5α (1 × 10^6^ CFU/mL) under the same setup. Compared with the pristine PVDF membrane, the Air‐450/PVDF membrane demonstrated superior bacterial removal efficiency (Figure 7d), as evidenced by its significantly lower lg (C/C_0_) values in the effluent samples at all time points (see Figure S41 for images of the corresponding bacterial colonies). Further analysis of amp^R^ in the effluent samples showed a pronounced, time‐dependent decrease with the Air‐450/PVDF membrane (Figure 7e), whereas amp^R^ levels were minimally changed with the pristine PVDF membrane. The structural stability of Air‐450 was assessed after 10 h of reaction (Figure S42). The unchanged XRD pattern confirms the preservation of the fluorite structure during prolonged operation. SEM and TEM images show negligible changes in the texture of the carbon framework and the supported CeO_2_ NPs, confirming excellent stability for continuous reactions. Effluent solutions were further collected at specific time points (15 min, 1 h, 2 h, up to 10 h) to assess Ce leaching. As shown in Figure S43, Ce leaching reached 0.041 mg/L at 15 min, attributed to residual Ce precursors from the unwashed sample that did not contribute to activity (Figure S18), and subsequently remained negligible at ca. 0.02 mg/L, close to the detection limit, throughout the testing period.

**FIGURE 7:**
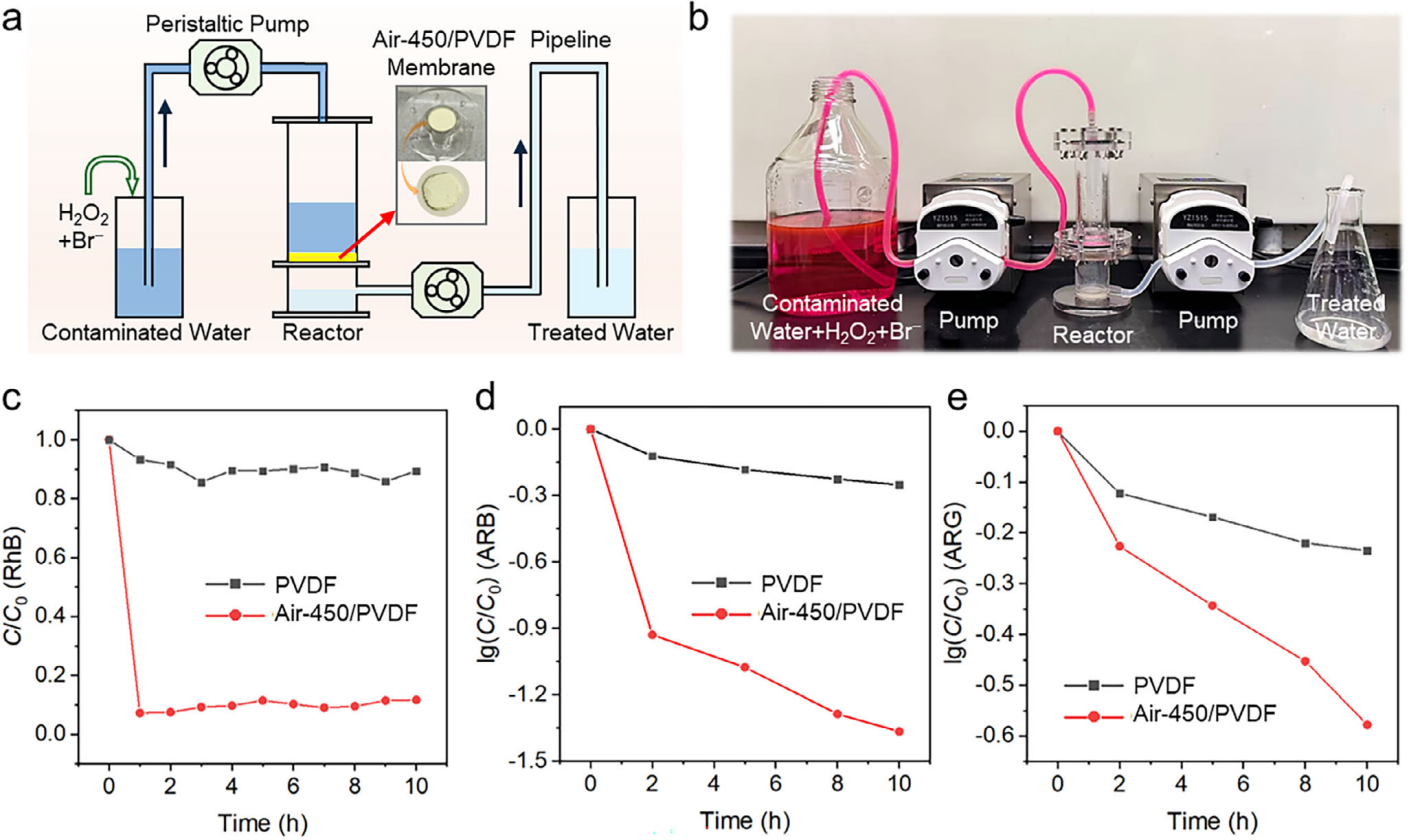
(a) Schematic diagram of the continuous‐flow reactor. The inset shows a photograph of the prepared Air‐450/PVDF membrane. (b) Photograph of the experimental setup using RhB as a model contaminant. Time‐dependent removal of (c) RhB, (d) *E. coli* DH5α, and (e) amp^R^ gene in the effluent samples collected at different time points. *C*_0_ and *C* denote their concentrations in the initial influent and in the effluent at each time point, respectively.

## 3 Conclusion

In summary, we showed that ultrasmall CeO_2_ NPs supported on a carbon framework can be readily obtained by air‐calcining a pre‐mixed Ce(NO_3_)_3_ and PVP. This approach is scalable (>50 g per batch in our laboratory oven) and requires no labor‐intensive purification, making it practical for applications. The optimized sample, Air‐450, features electron‐lean Ce sites that suppress H_2_O_2_ activation via the radical pathway while promoting the nonradical pathway, thereby facilitating Br^−^‐mediated H_2_O_2_‐to‐^1^O_2_ conversion. By contrast, the N‐coordinated Ce sites in N_2_‐450 are sufficiently electron‐rich and hence follow the radical pathway, converting H_2_O_2_ into OH radicals. Such precise control of the H_2_O_2_ reaction pathway is shown to be closely associated with the well‐separated δ^31^P signals of their Ce sites. Notably, Air‐450 not only closely mimics BPOs in producing HOBr but also catalyzes its subsequent conversion to ^1^O_2_ with adsorbed H_2_O_2_ (or peroxo species) on nearby Ce sites. Using RhB as a model contaminant, we show that this bio‐inspired nonradical H_2_O_2_ activation is superior to the typical Fenton/Fenton‐like radical pathway in removal efficiency and retains activity across a wide pH range and in the presence of common environmental ions. Similar results were observed for the removal of the antibiotic ARB and its associated ARGs, suggesting that although OH radicals are widely used, their effectiveness is inherently limited by various scavenging reactions. This work thus provides a scalable method for preparing catalysts with finely tuned electron density for exclusive nonradical H_2_O_2_ activation and improved performance, guiding the future design of practical catalysts to combat antibiotic resistance.

## 4 Method

### 4.1 Samples Preparation

First, polyvinylpyrrolidone (PVP) with a molecular weight of 1.3 million and Ce(NO_3_)_3_·6H_2_O (weight ratio of 1:1.5) were added to deionized water, followed by stirring at 70°C for 0.5 h until completely dissolved. Calcination of the mixture was performed in a muffle furnace for samples prepared in an air atmosphere, or in a tube furnace for samples synthesized in N_2_ (N_2_ flow rate: 100 mL/min). The heating protocol involved ramping at 5°C/min to the target temperature, holding for 1 h, then natural cooling to room temperature. Additionally, as described previously [71], typical CeO_2_ nanoparticles (ty‐Ce) were synthesized by stirring 0.3 g NaOH with 1.3 g Ce(NO_3_)_3_ in 96 mL of deionized water at room temperature for 22 h.

### 4.2 Detection of HOBr

The phenol red (PR) bromination assay was utilized to assess the generation of HOBr. The 1.5 mL reaction solution contained 0.2 g/L catalyst (or 0.05 U bromoperoxidases), 1 mM H_2_O_2_, 0.84 mM KBr, and 0.04 mM PR. UV–vis spectroscopy was recorded for 30 min at 1 min intervals. The PR and Br_4_PR were monitored at 430 and 590 nm, respectively.

#### 4.2.1 Degradation of Rhodamine B (RhB)

The degradation of RhB was monitored using a UV–vis spectrophotometer. The 1.5 mL cuvette contained 0.2 g/L samples (or 0.05 U bromoperoxidases), 1 mM H_2_O_2_, 0.84 mM KBr, and 20 ppm RhB. UV–vis spectroscopy was recorded for 30 min at 1 min intervals. The existence of RhB can be monitored at 554 nm. To investigate the effects of KBr and H_2_O_2_ concentration on the degradation of RhB, 0.5–50 mM KBr and 1 mM H_2_O_2_ were used for detection. Similarly, reaction solutions containing H_2_O_2_ at various concentrations (0.5–50 mM) with 0.84 mM KBr, and H_2_O_2_ at 0.05–10 mM with 30 mM KBr, were prepared for evaluation, respectively.

### 4.3 Electron Paramagnetic Resonance (EPR) Measurement

EPR measurements were carried out at ambient temperature on an ADANI SpinscanX spectrometer, operating at 100 kHz field modulation with a modulation amplitude of 150 µT. 5,5‐dimethyl‐1‐pyrroline N‐oxide (DMPO) and 2,2,6,6‐tetramethylpiperidine (TEMP) were selected as the trappers for the detection of OH/HO_2_ radicals and singlet oxygen (^1^O_2_), respectively. Typically, 5 µL DMPO or 100 µL TEMP (250 mM) was added to 1 mL of solution with 0.2 g/L samples (or 0.05 U bromoperoxidases), 100 mM H_2_O_2_ in the presence and absence of 20 mM KBr. Note that the analysis of the HO_2_ radical was conducted in methanol because DMPO‐OOH has relatively slow kinetics of formation (cf. DMPO‐OH). The mixture was allowed to react for 30 min. Subsequently, the supernatant obtained through centrifugation was used for the EPR measurement. Note that the concentrations of H_2_O_2_ and KBr used in the EPR experiments are higher than those employed for RhB degradation due to the inherent sensitivity limitations of the spin‐trapping EPR technique.

### 4.4 Nuclear Magnetic Resonance (NMR) Measurement

A total of 150 mg of samples were first placed in a home‐made glass tube, pretreated at 75°C at 10^−1^ Pa for 2 h to clean the surface. The treated sample was then mixed with 0.1 M TMPO in CH_2_Cl_2_ solvent. The resulting mixture was sonicated for 5 min, then dried under vacuum. ^31^P NMR spectroscopy was collected using a Bruker AVANCE NEO 400 M spectrometer.

### 4.5 Disproportionation of H_2_O_2_

The study of H_2_O_2_ disproportionation was performed using 15 mL reaction solution by mixing 0.2 g/L samples (or 0.05 U bromoperoxidases) and 20 mM H_2_O_2_ with or without 20 mM KBr. To avoid interference from the samples, 1.5 mL of solution was collected every 15 min, and the corresponding supernatant was collected by centrifugation for analysis; the total reaction time was 120 min. Time‐dependent decomposition of H_2_O_2_ was monitored by measuring the absorbance at its characteristic peak of 240 nm via UV–vis spectroscopy.

### 4.6 HPLC‐MS

The reaction mixture was prepared by mixing 300 µL of an Air‐450 suspension (1 mg/mL), 150 µL of H_2_O_2_ (10 mM), 126 µL of KBr (10 mM), 150 µL of RhB (200 mg/L), and 774 µL of water, giving a total volume of 1.5 mL. Aliquots were collected after 10 and 30 min of reaction and passed through membrane filters before HPLC–MS analysis using a SCIEX X500R QTOF mass spectrometer coupled to an HPLC system. Chromatographic separation was performed on a Gemini C18 column (250 × 4.6 mm, 5 µm, 100 Å) maintained at 35°C. The mobile phases were water containing 0.1% formic acid (A) and acetonitrile containing 0.1% formic acid (B). The flow rate was 0.30 mL/min, and the injection volume was 5 µL. The gradient elution program was as follows: 5% B for 0–1 min; a linear increase from 5% to 50% B over 1–8 min; a linear increase from 50% to 95% B over 8–12 min; 95% B for 12–18 min; a return to 5% B over 18–18.2 min; and re‐equilibration at 5% B until 20 min.

### 4.7 Ion chromatography

To monitor BrO_3_^−^ formation, the reaction was performed under the same conditions used for RhB degradation, consisting of 0.2 g/L Air‐450, 1 mM H_2_O_2_, and 0.84 mM KBr. After 30 min of reaction, the catalyst was removed by filtration. The resulting supernatant was analyzed using a Dionex Aquion IC system equipped with an AS19 anion analytical column. The mobile phase consisted of 7.19 mM Na_2_CO_3_ and 3.21 mM NaHCO_3_. The chromatographic separation was conducted at a column temperature of 30°C with a constant flow rate of 0.9 mL/min. The injection volume was set to 250 µL.

#### 4.7.1 pH and Temperature Stability

Air‐450 and bromoperoxidase were incubated for 3 h under varying pH (3–14) and temperature (0°C–65°C) conditions. Following incubation, their RhB degradation activities were determined. The reaction solution is 1.5 mL containing 0.2 g/L Air‐450 (or 0.05 U bromoperoxidases), 1 mM H_2_O_2_, 0.84 mM KBr, and 20 ppm RhB.

#### 4.7.2 Degradation of Ampicillin (AMP)

The reaction solution contained 0.2 g/L samples, 1 mM H_2_O_2_, 30 mM KBr, and 20 ppm AMP, and the total volume was 15 mL. The suspension was continuously stirred to inhibit sedimentation of the catalyst. At specific time intervals, 1 mL of the reaction solution was collected and filtered through a 0.22 µm glass fiber membrane to remove the catalyst; the supernatant was then analyzed for AMP concentration by High Resolution Mass Spectrometer (HPLC, SCIEX ExionLC AD System, and a SCIEX X500R Q‐TOF mass spectrometer). The mobile phase was a mixture of 20 mM formic acid aqueous solution (solvent A) and acetonitrile containing 20 mM formic acid (solvent B), at a flow rate of 0.5 mL/min. Separation was performed using a linear gradient from 5:95 (solvent B/solvent A) to 95:5 for 10 min, 95:5 for the following 3 min, and a linear gradient from 95:5 to 5:95 within the following 2 min, and then 5:95 for 2 min of equilibrium.

### 4.8 Antibiotic‐Resistant Bacteria (ARB) Degradation

The strain *E. coli* DH5α was purchased from Biobw Biotechnology Co., Ltd. (Beijing, China). *E. coli* DH5α harbors the self‐transferable plasmid RP4 carrying the ampicillin (amp) resistance gene. The antibacterial activity of Air‐450 and N_2_‐450 was tested against *E. coli* DH5α, which was cultured in Luria–Bertani (LB) broth at 37°C for 18 h. The concentration of *E. coli* DH5α was set to an OD600 of 1.0 by dilution in PBS. The bacterial suspension was subsequently supplemented with 500 mM KBr and 1 mM H_2_O_2_, followed by treatment with 6 mM catalysts. The treated bacterial suspension was subjected to 10‐fold serial dilutions in PBS after 15 min of incubation. 0.1 mL of each dilution was plated in triplicate on agar and incubated at 37°C for 18 h, after which colonies were counted and the numbers multiplied by the dilution factor to obtain CFU/ mL.

### 4.9 Deoxyribonucleic Acid (DNA)/Antibiotic‐Resistant Gene (ARG) Degradation

To further evaluate the DNA degradation after cell disruption caused by Air‐450 and N_2_‐450 particles, *E. coli* DH5α carrying plasmid RP4 was used as a model strain. In this study, degradation of both extracellular DNA (eDNA) or ARG (eARG) and intracellular DNA (iDNA) or ARG (iARG) was assessed. The initial concentration of *E. coli* DH5α was adjusted to OD600 = 1, together with certain concentrations of catalysts (6 mM), KBr (500 mM), and H_2_O_2_ (1 mM), as in the antibacterial activity assay. Then the mixture of cells and catalysts was cultivated at ambient temperature 25°C with constant shaking. The samples were collected at different time intervals (0, 5, 10, 20, 60, and 120 min), and the reaction was stopped by adding Na_2_SO_3_. Intracellular and extracellular DNAweres extractedfromy 1 mL of samples using the TIANamp Bacteria DNA Kit (TIANGEN, Beijing, China) following the instructions. The concentrations of collected DNA were determined with a Qubit 3.0 Nanodrop One UV–vis Spectrophotometer (Thermo Fisher Scientific China Co., Ltd). The intracellular and extracellular ARG (*amp^R^*) were analyzed by real‐time quantitative PCR, using TB Green Premix Ex Taq II (Tli RNaseH Plus) (Takara, Kyoto, Japan) in (QuantStudio3, Thermo Fisher Scientific China Co., Ltd, Shanghai, China).

### 4.10 Assessment Using a Continuous Flow System

The deionized water with 20 ppm RhB (or 1 × 10^6^ CFU/mL of *E. coli* DH5α) was initially mixed with 30 mM KBr and 1 mM H_2_O_2_. This resulting mixture was subsequently introduced into the continuous flow reactor via a peristaltic pump (2 mL/min). The reaction happened once the mixture solution met the Air‐450 coated on a polyvinylidene difluoride (PVDF) membrane. After the reaction, the treated water was pumped out by another peristaltic pump, and approximately 6 mL samples were collected at specified time points for analysis. To exclude the influence of adsorption on PVDF, samples were also collected at identical time points using PVDF membranes alone without any catalyst for comparison.

### 4.11 Density Functional Theory (DFT) Calculation

Spin‐polarized first‐principles calculations were performed within the framework of density functional theory (DFT) using the Vienna *Ab‐Initio* Simulation Package (VASP) [72]. The Perdew–Burke–Ernzerhof (PBE) [73] functional under the generalized gradient approximation (GGA) was adopted to treat exchange‐correlation effects. Electron‐ion interactions were described by projector augmented wave (PAW) [74, 75] pseudopotentials with a cut‐off energy of 460 eV. Brillouin zone sampling for bulk CeO_2_ was conducted on a 4 × 4 × 4 *k*‐mesh. Given the highly localized nature of Ce 4f electrons, the DFT + U correction scheme proposed by Dudarev [76] was employed with the value of 3.5 eV to properly capture the strong on‐site Coulomb interactions.

Five‐layer CeO_2_(111) slab models were constructed using $4\times 2\sqrt{3}R30^{\circ }$ supercells, respectively, yielding the surface areas of 2.07 nm^2^ [77]. These supercell sizes were chosen to maintain comparable adsorbate coverages across different facets. In all slab models, the bottom three layers were fixed to mimic the bulk‐like region, while the top two layers were fully relaxed during structural optimization. A vacuum layer of 20Å was introduced to eliminate the spurious interactions between the periodic images in the *z*‐direction. The adsorption energy (*E*_ads_) was calculated by the equation: *E_ads_* = *E_total_* – *E_surface_* – *E_adsorbate_*, where *E_ads_* is the total energy of the combined adsorbate–surface system, and *E_surface_* and *E_adsorbate_* are the energies of the bare slab and the gas‐phase adsorbate molecules, respectively. The relative thermodynamic stability of distinct N‐doped CeO_2_(111) configurations was evaluated through the formation energy *E_f_*, defined as: *E_f_* = *E_N‐doped_* – *E_surface_* + *xµ_O_* – *yµN*. Here, *E_N‐doped_* denotes the total energy of the nitrogen‐incorporated slab, while the chemical potentials *µ_O_* and *µN* were extracted from the total energies of isolated O_2_ and N_2_. The integers *x* and *y* represent the numbers of oxygen vacancies created and nitrogen atoms substituted, respectively. A more negative *E_f_* indicates a thermodynamically favorable configuration. Charge transfer at the interface was quantitatively analyzed using the Bader analysis partition scheme [78].

## Author Contributions

**Bo Yuan**: writing – review and editing, writing – original draft, formal analysis, investigation, validation, data curation. **Xinyu Wu**: writing – original draft, investigation, validation, formal analysis, data curation. **Yin‐Song Liao**: writing – original draft, investigation, validation, formal analysis, data curation. **Chao Zhao**: data curation, investigation. **Zhanping Xiao**: data curation, investigation. **Wanqing Dai**: investigation, data curation. **Jyh‐Pin Chou**: supervision, writing – original draft, investigation, validation, formal analysis. **Wenchao Peng**: investigation, conceptualization. **Jian Lin Chen**: writing – original draft, investigation, validation, formal analysis, supervision. **Pi‐Tai Chou**: conceptualization, resources. **Yung‐Kang Peng**: writing – original draft, writing – review and editing, funding acquisition, investigation, conceptualization, validation, formal analysis, supervision.

## Conflicts of Interest

The authors declare no conflicts of interest.

## Supporting information




**Supporting File**: advs78122‐sup‐0001‐SuppMat.pdf.

## Data Availability

The data that support the findings of this study are available from the corresponding author upon reasonable request.
