# Supplementary material for: Electronic‐State Programming of Ultrasmall CeO2 Switches H2O2 Activation From Radical to Bio‐Inspired Nonradical 1O2 Generation for Combating Antibiotic Resistance
Source: Adv Sci (Weinh). 2026 Sep 29:e78122. Online ahead of print. doi: 10.1002/advs.78122 (PMC13624328; doi:10.1002/advs.78122)
Supplement: Supplementary file 1 [file ADVS-9999-e78122-s001.pdf]

Supplementary Information for

Electronic-State Programming of Ultrasmall CeO_2 Switches H_2O_2 Activation from Radical to Bio-Inspired Nonradical $^1O_2$ Generation for Combating Antibiotic Resistance

Bo Yuan,[1#] Xinyu Wu,[1,2#] Yin-Song Liao,[3#] Chao Zhao,[1] Zhanping Xiao,[1,4] Wanqing Dai,[2] Jyh-Pin Chou,[3*] Wenchao Peng,[5] Jian Lin Chen,[2*] Pi-Tai Chou[4] and Yung-Kang Peng[1*]

[1]Department of Chemistry, City University of Hong Kong, Hong Kong 999077, Hong Kong SAR.

[2]Department of Applied Science, School of Science and Technology, Hong Kong Metropolitan University, Hong Kong 999077, Hong Kong SAR.

[3]Graduate School of Advanced Technology, National Taiwan University, Taipei 106319, Taiwan.

[4]Department of Chemistry, National Taiwan University, Taipei 106319, Taiwan.

[5]School of Chemical Engineering and Technology, Tianjin University, Tianjin 300072, China

[#]These authors contribute equally to this work

*Correspondence: ykpeng@cityu.edu.hk; jchen@hkmu.edu.hk; jpchou@ntu.edu.tw

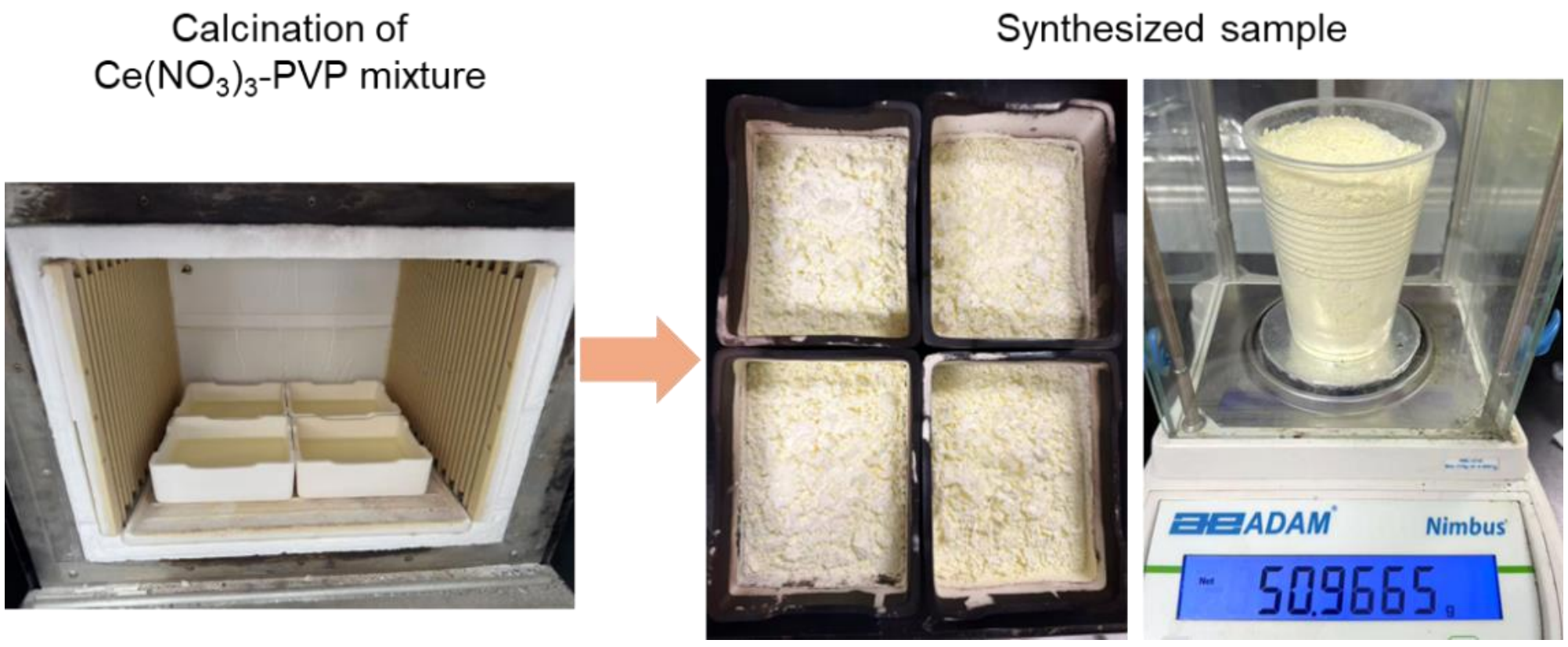

Figure S1. Gram-scale production for the sample (calcined at 450°C in air) and their quantities produced in each run. Note that the sample can be used directly without the need for purification.

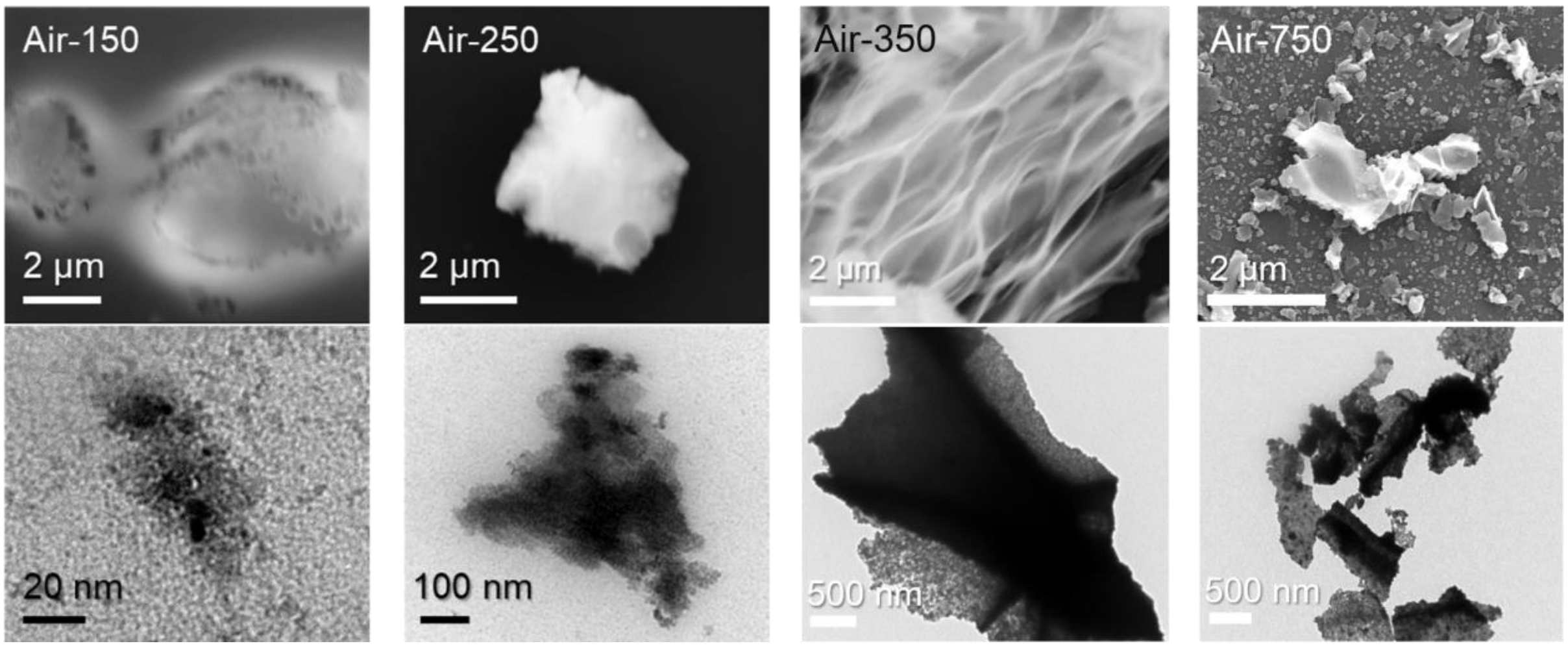

Figure S2. SEM (top) and TEM (bottom) images of Air-150, Air-250, Air-350, and Air-750.

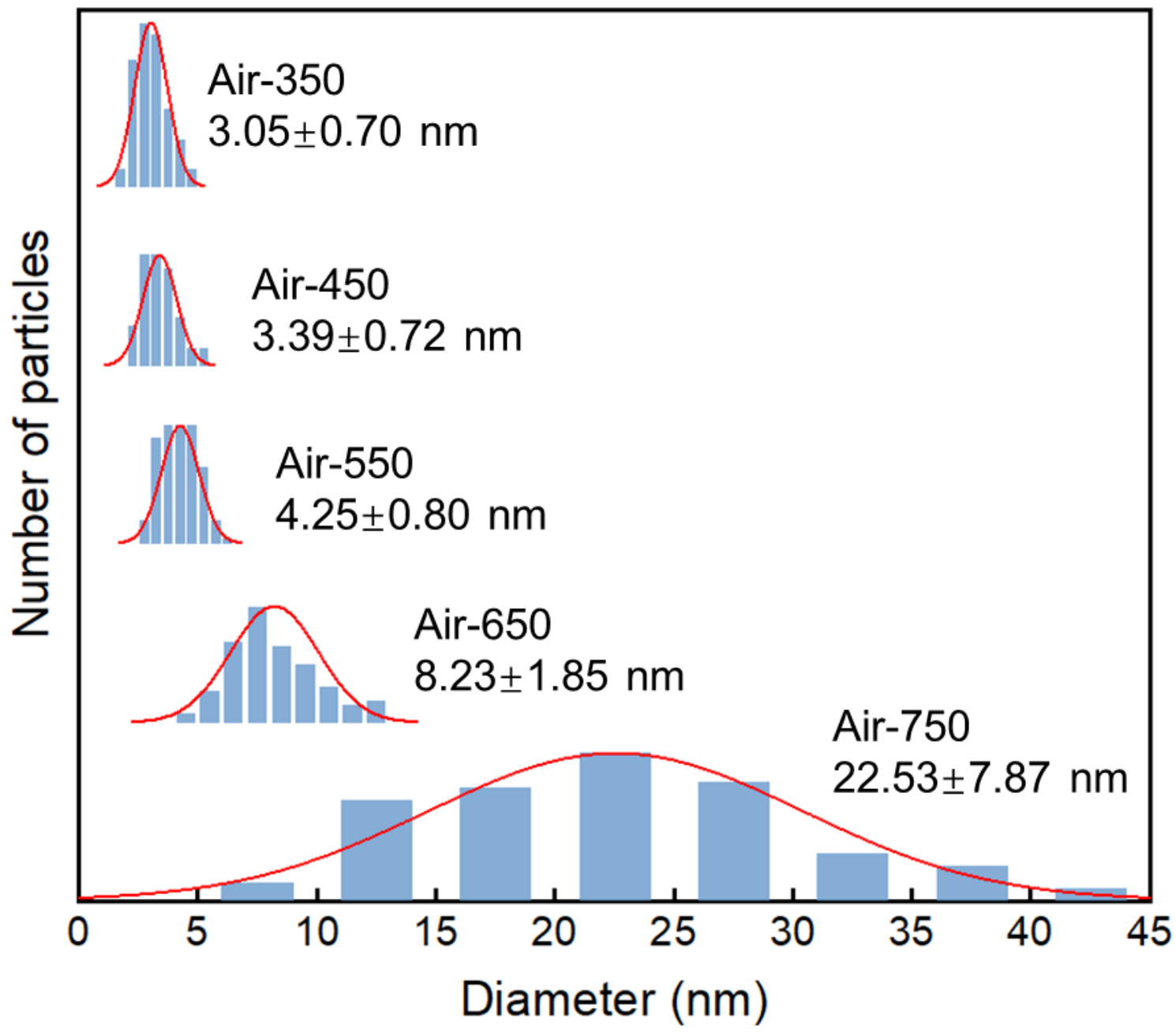

Figure S3. Particle size distributions of samples (100 particles were counted for each sample).

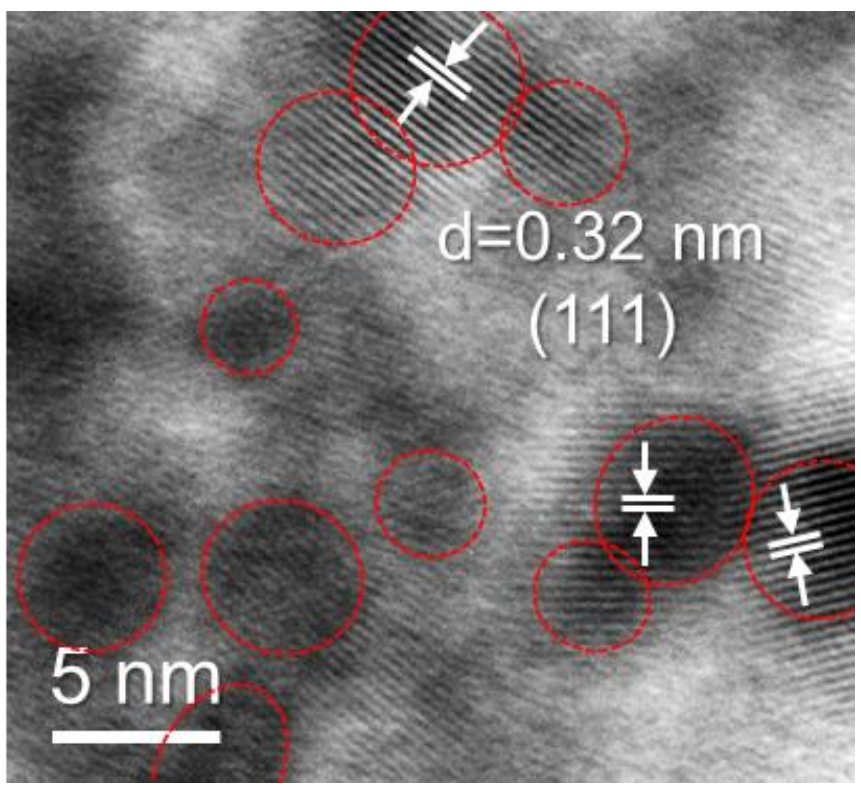

Figure S4. High-resolution TEM images of Air-450.

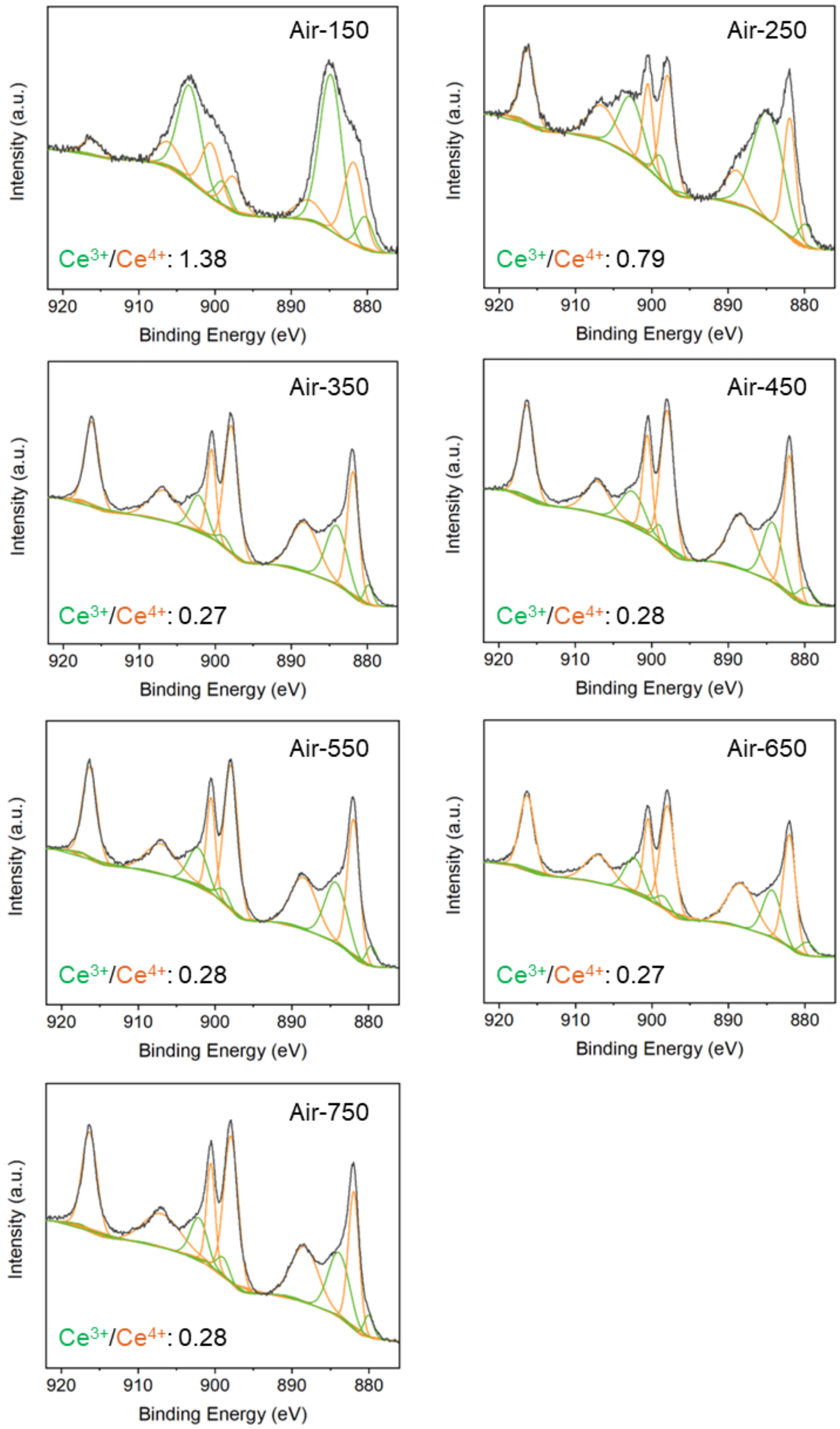

Figure S5. The deconvoluted XPS Ce_{3d} spectra of samples.

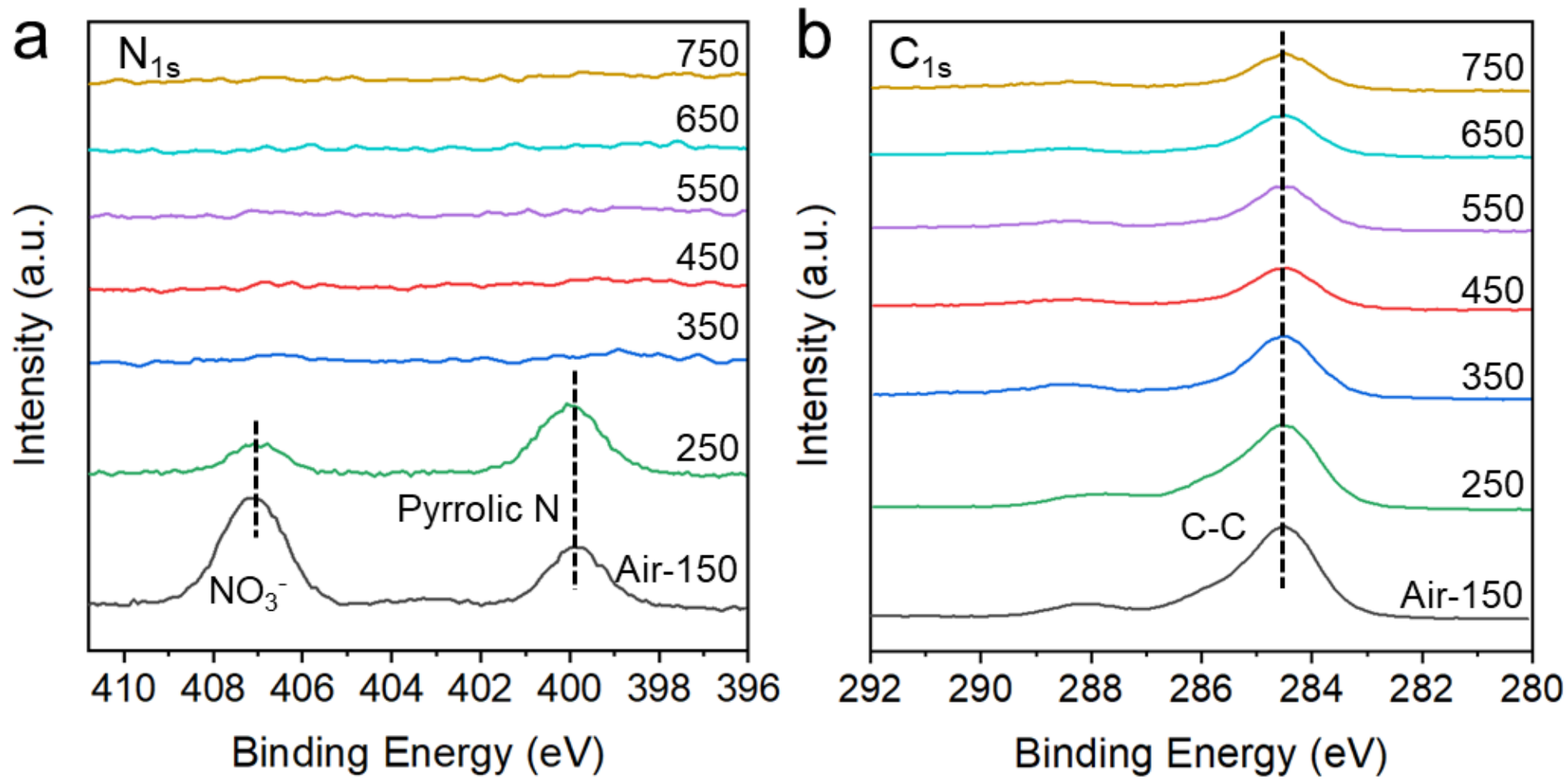

Figure S6. XPS analysis of samples in the regions of (a) N_{1s} and (b) C_{1s}.

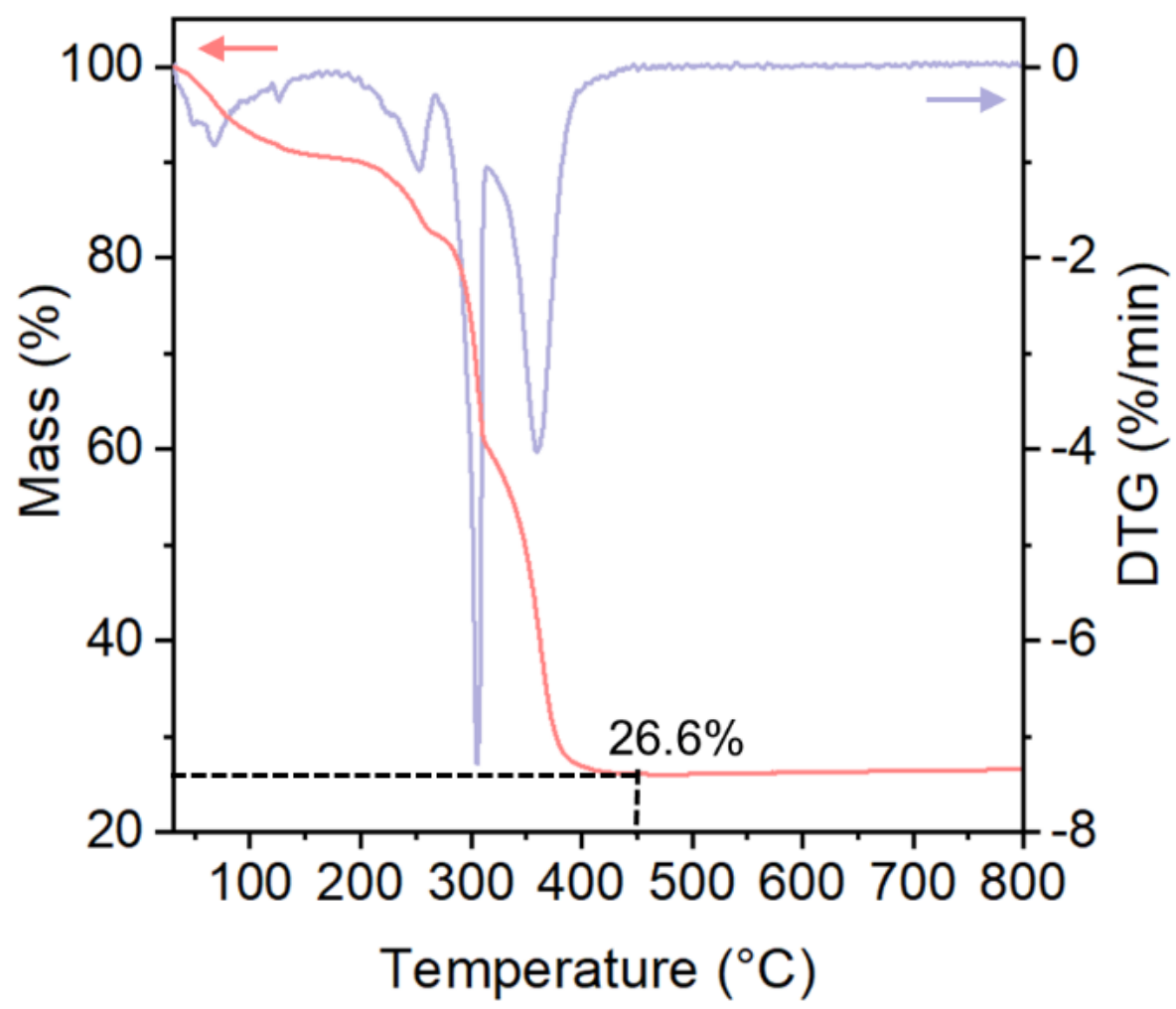

Figure S7. Thermogravimetric analysis of $Ce(NO_3)_3$ and PVP mixture calcined in air under elevated temperature.

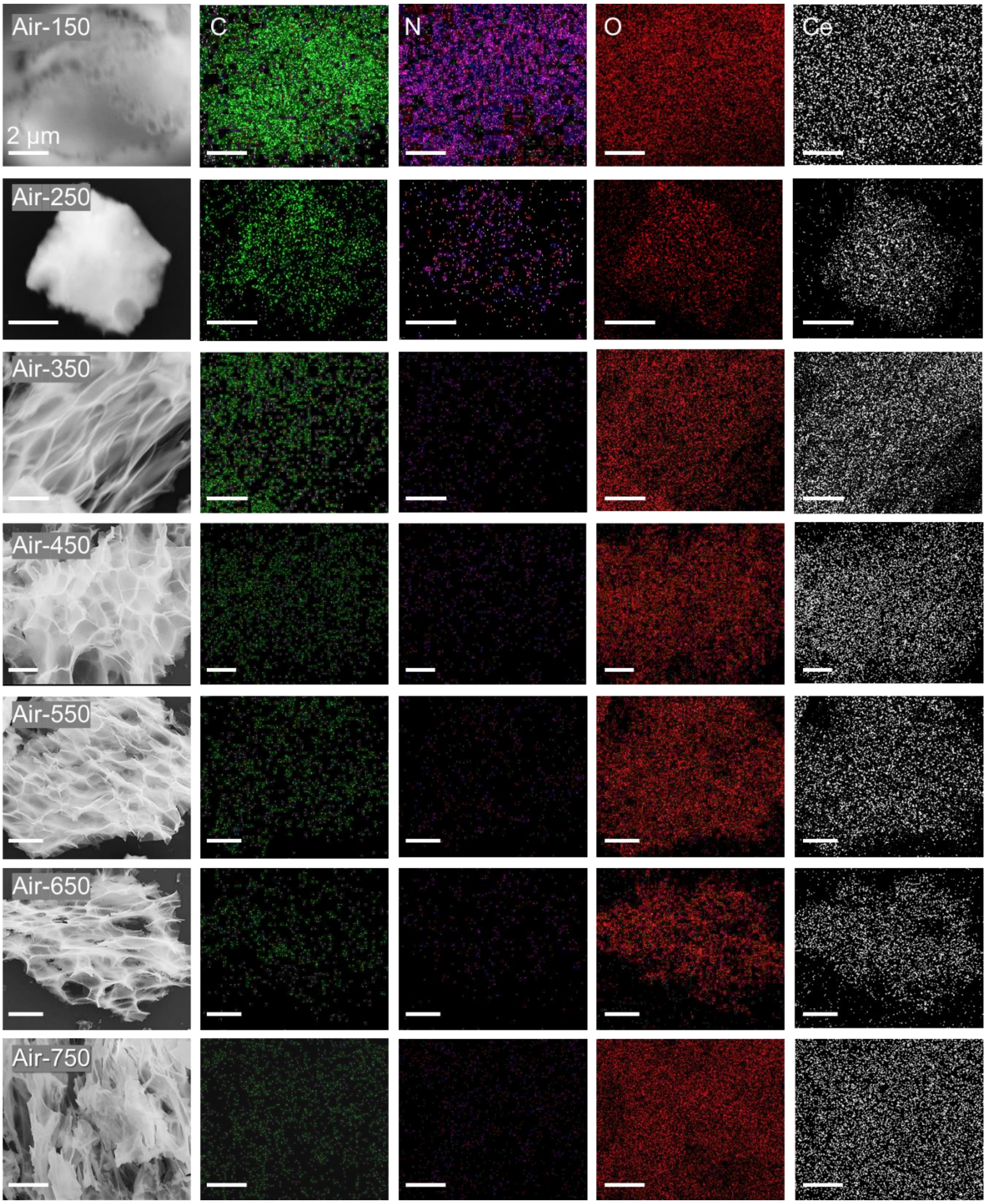

Figure S8. SEM elemental mapping of samples. All the scale bar is 2 µm.

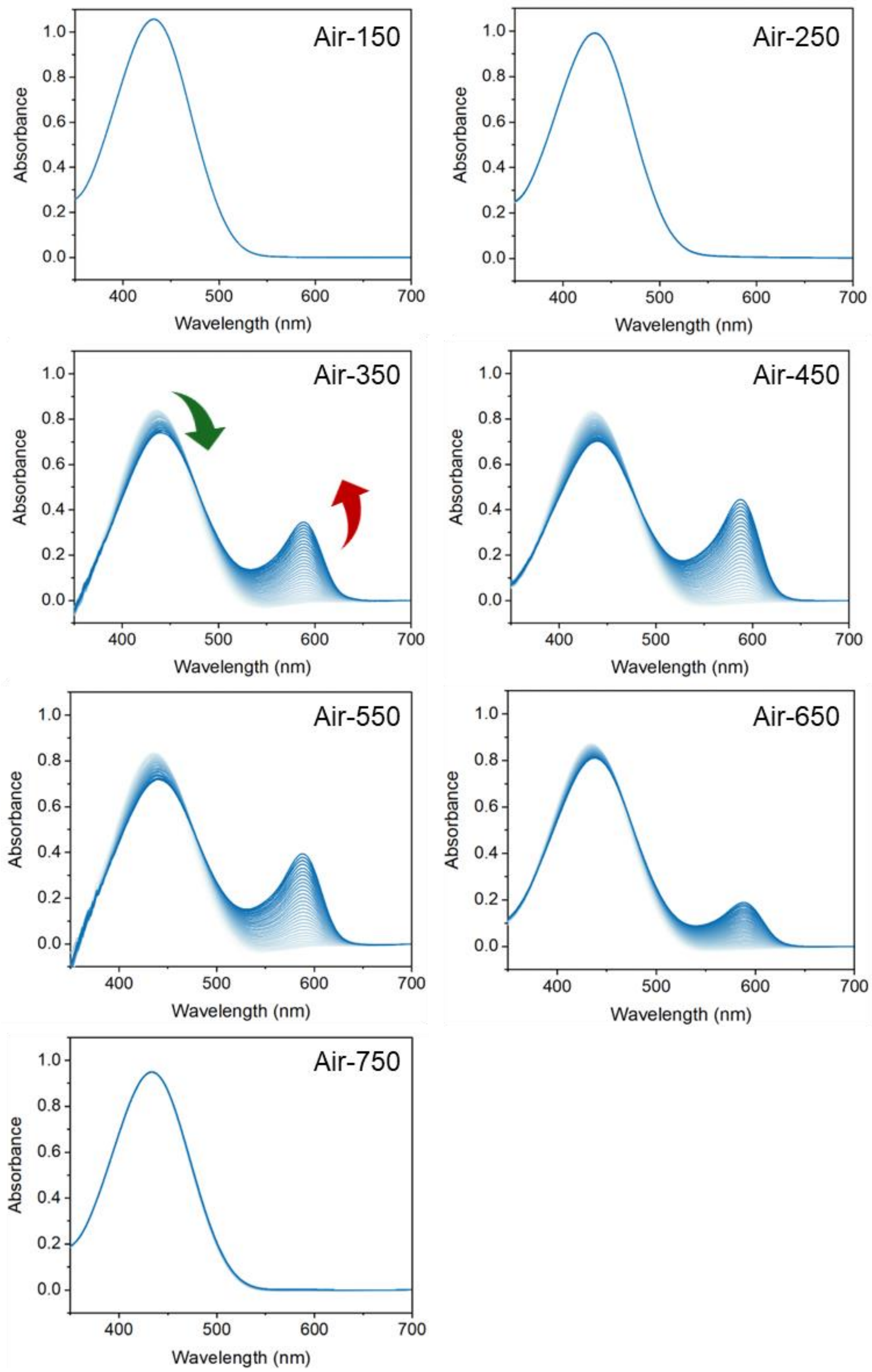

Figure S9. Time-dependent UV-vis spectra showing the generation of Br_4PR at 590 nm for the detection of HOBr over samples for 30 minutes.

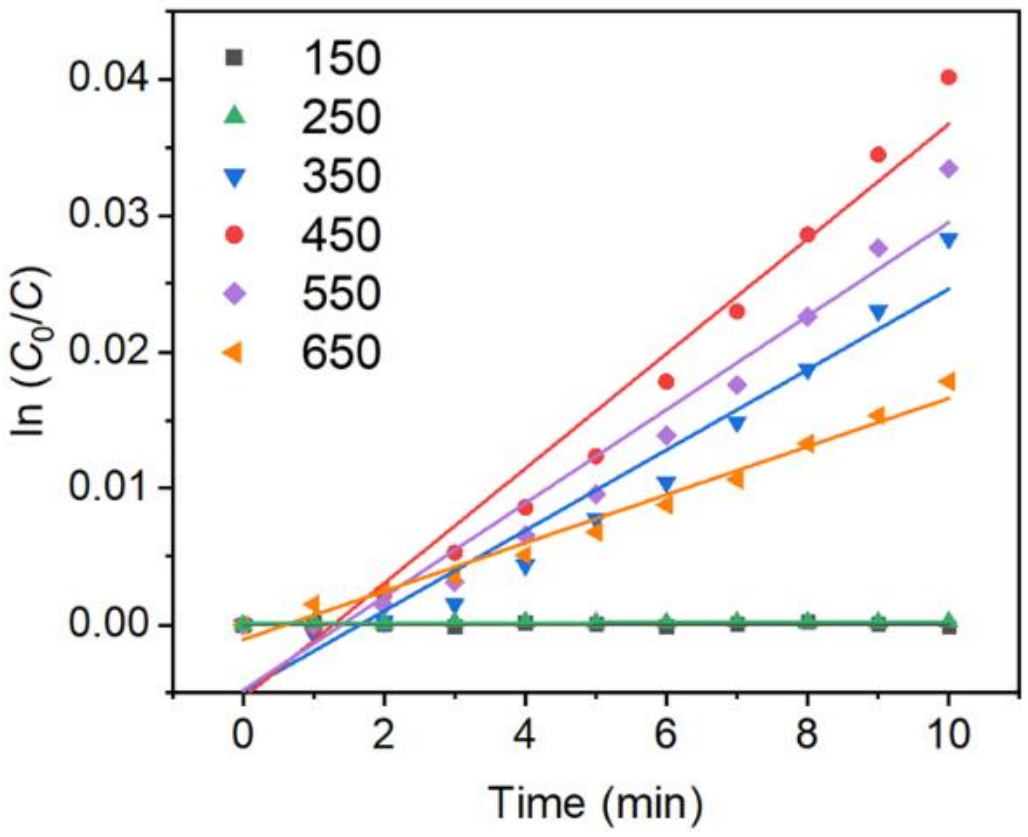

Figure S10. First-order kinetic fitting of Br_4PR production over samples.

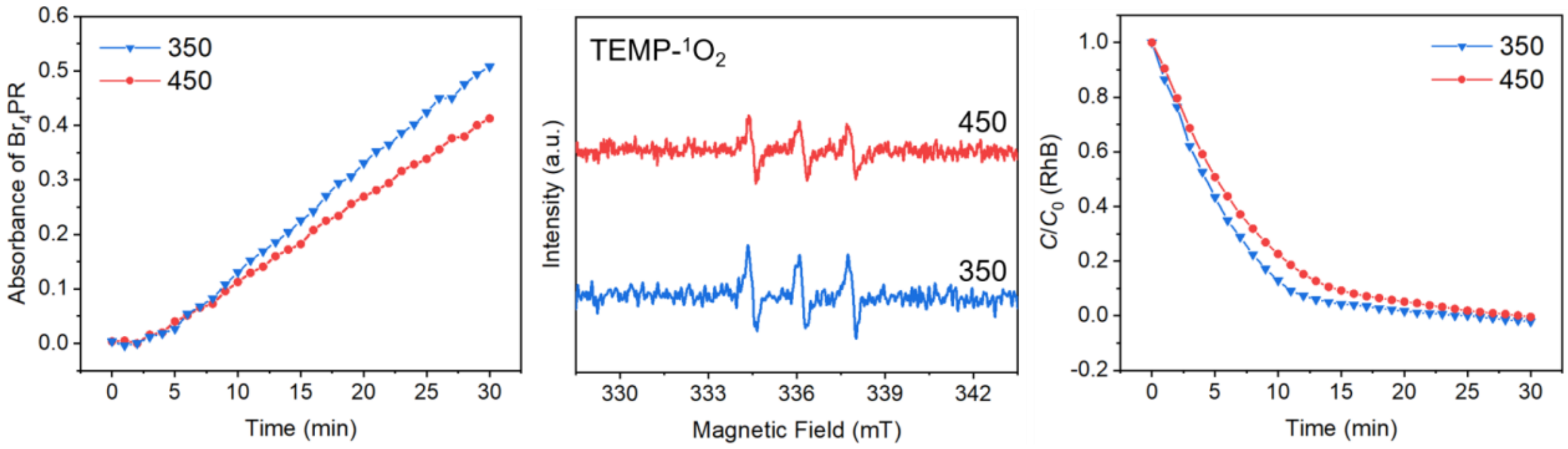

Figure S11. Catalytic performance of Air-350 (0.3 g/L) and Air-450 (0.2 g/L) at equivalent Ce loadings for HOBr production (left), $^1O_2$ generation (middle), and RhB degradation (right).

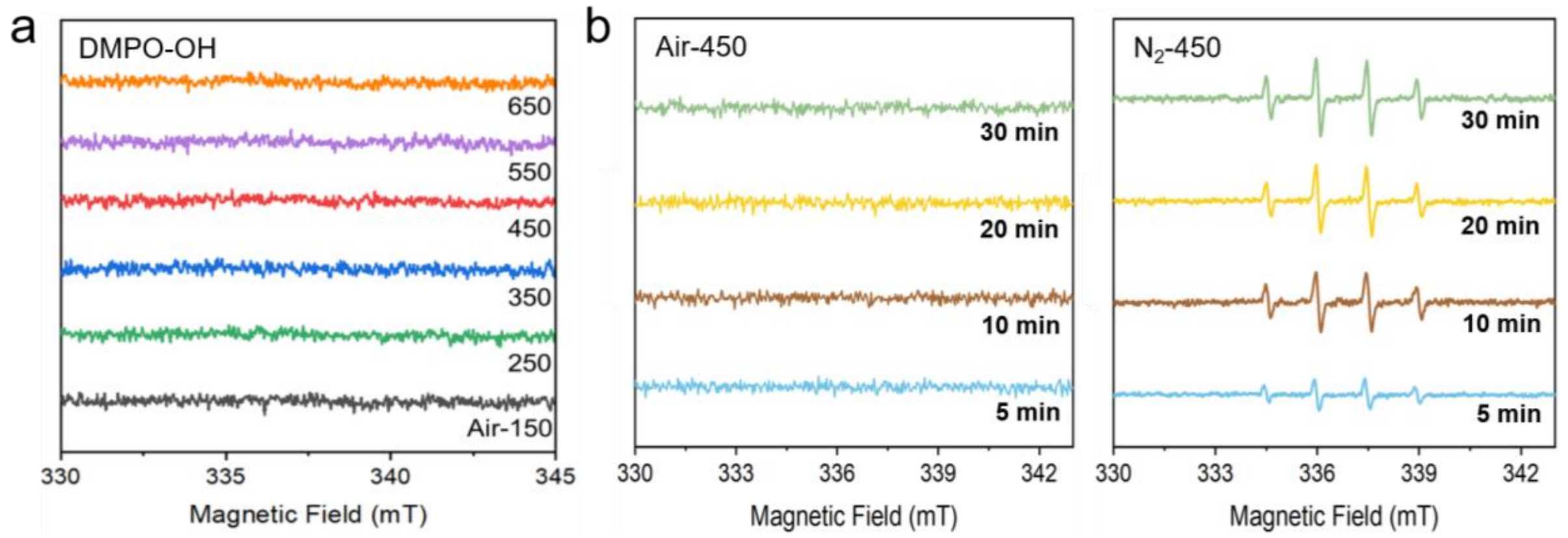

Figure S12. (a) DMPO-assisted EPR monitoring of OH radicals generation over air-calcined CeO_2 samples in 30 min, and (b) corresponding time-dependent results for Air-450 and N_2-450.

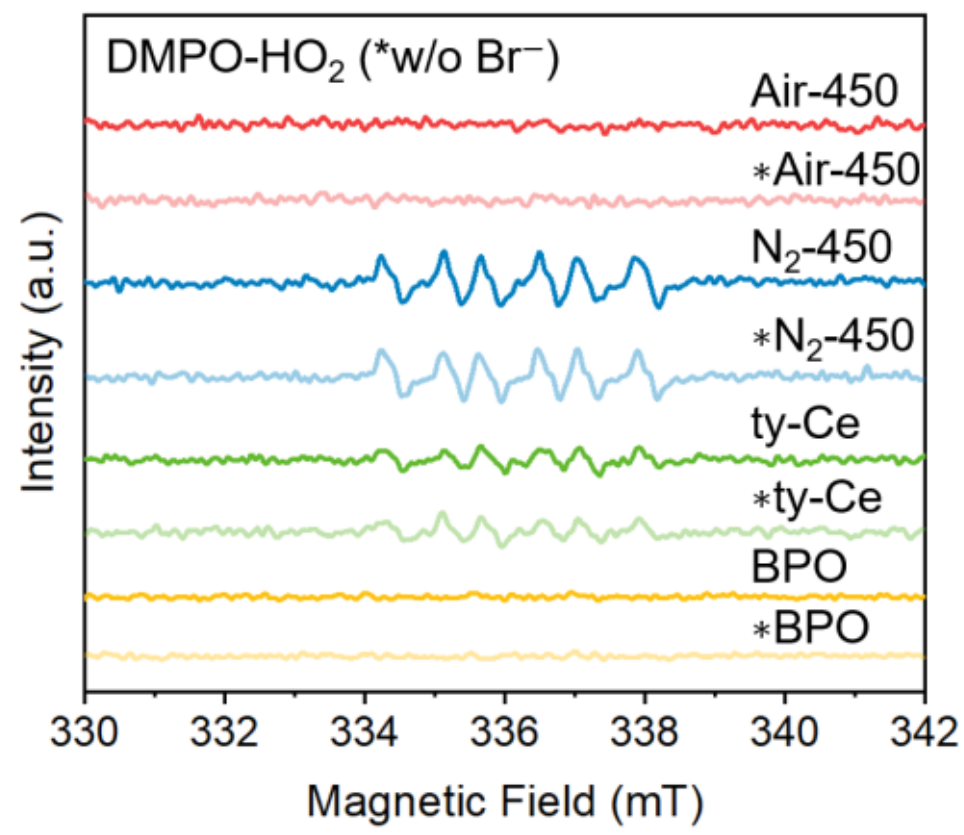

Figure S13. DMPO-assisted EPR monitoring of HO_2 radical generation (in methanol) over various CeO_2 samples and BPOs in 30 min.

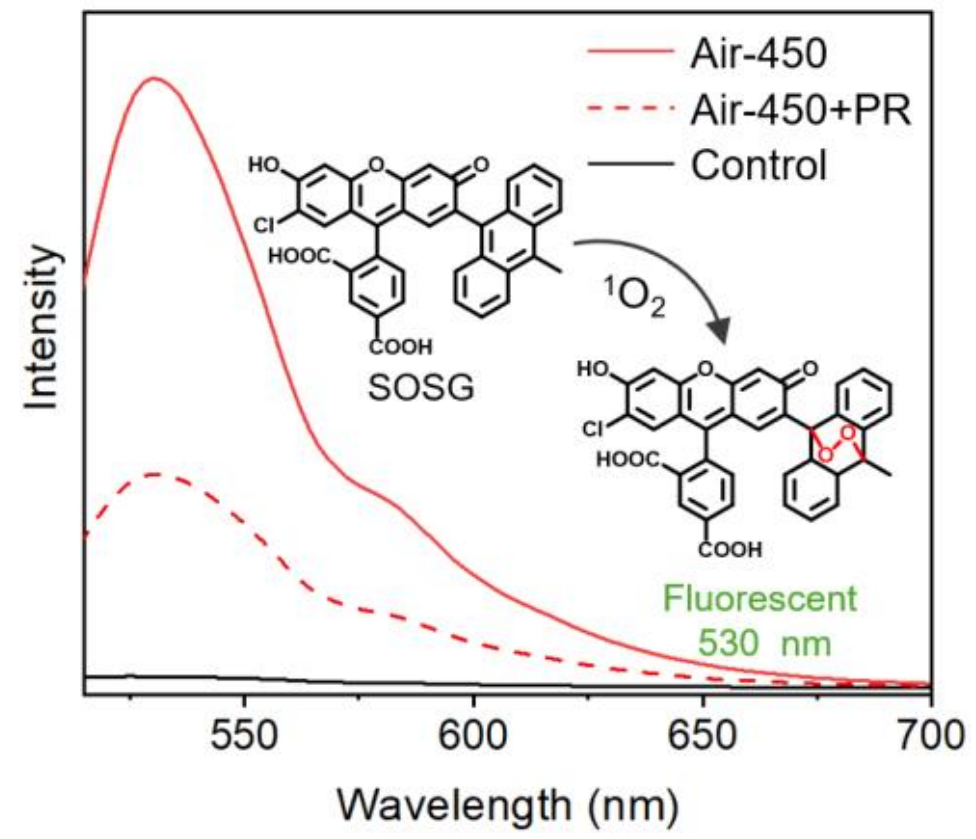

Figure S14. SOSG detection of $^1O_2$ production for the Air-450 + H_2O_2 + Br^- system in the absence/presence of PR.

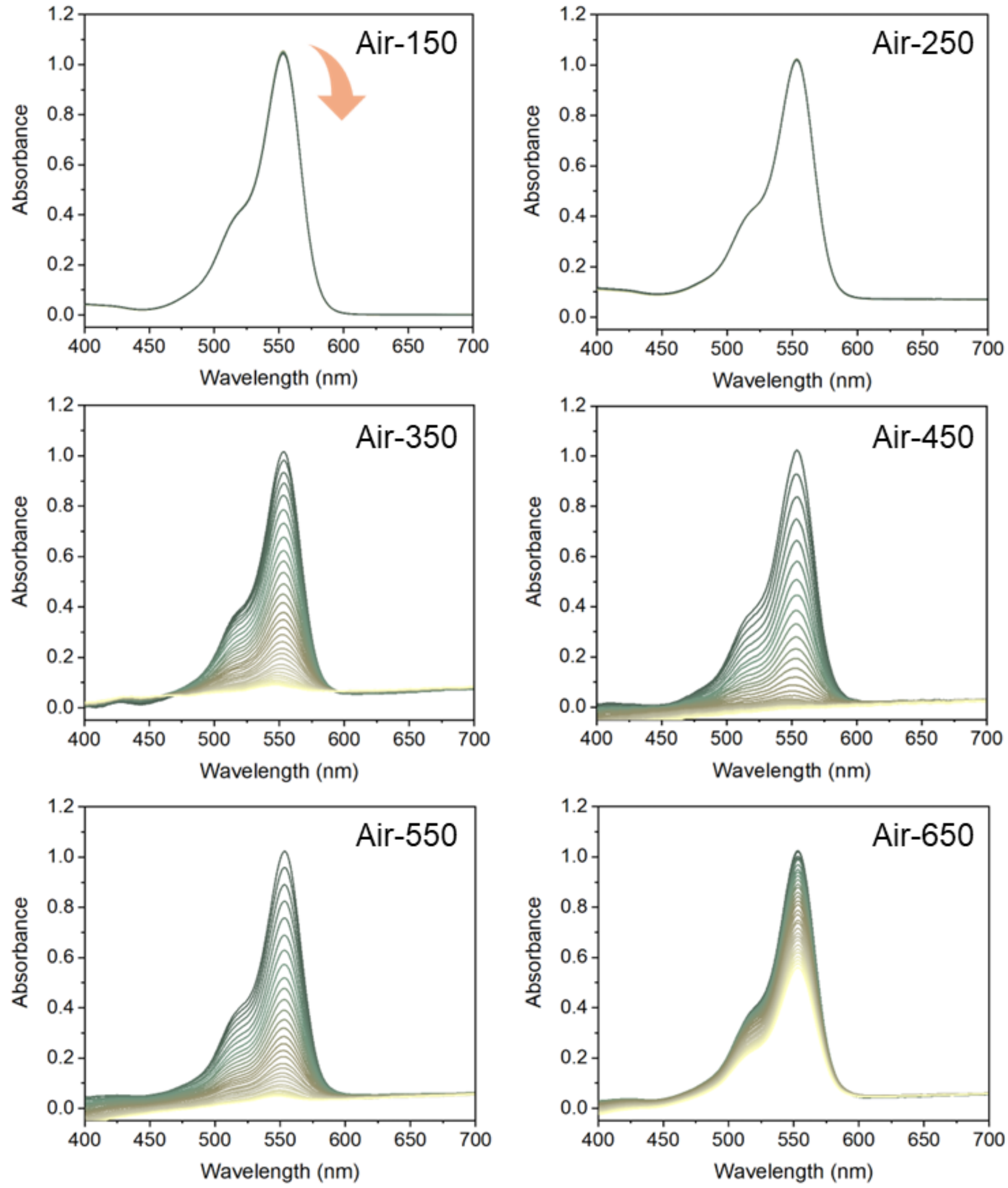

Figure S15. Time-dependent UV-vis spectra monitoring the degradation of RhB over samples for 30 minutes.

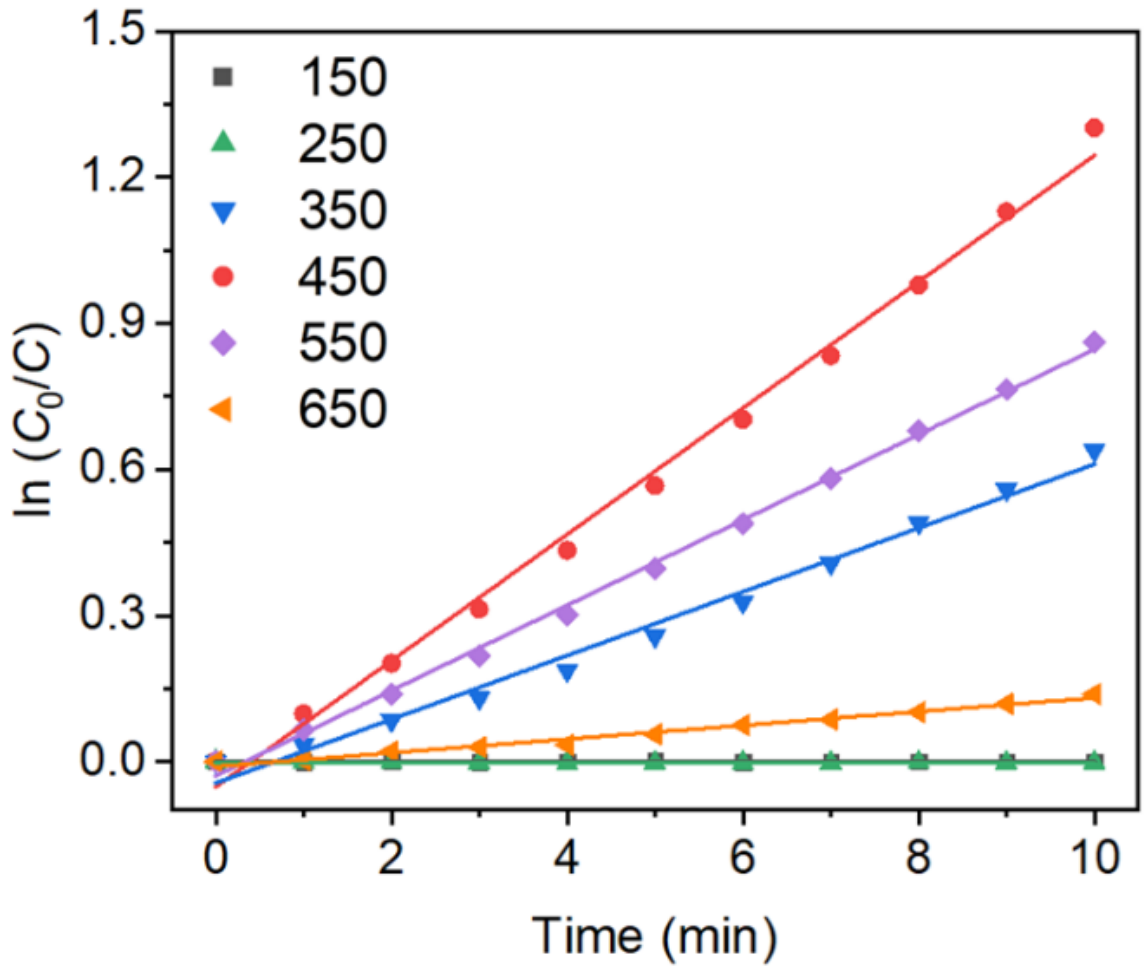

Figure S16. First-order kinetic fitting of RhB degradation over samples.

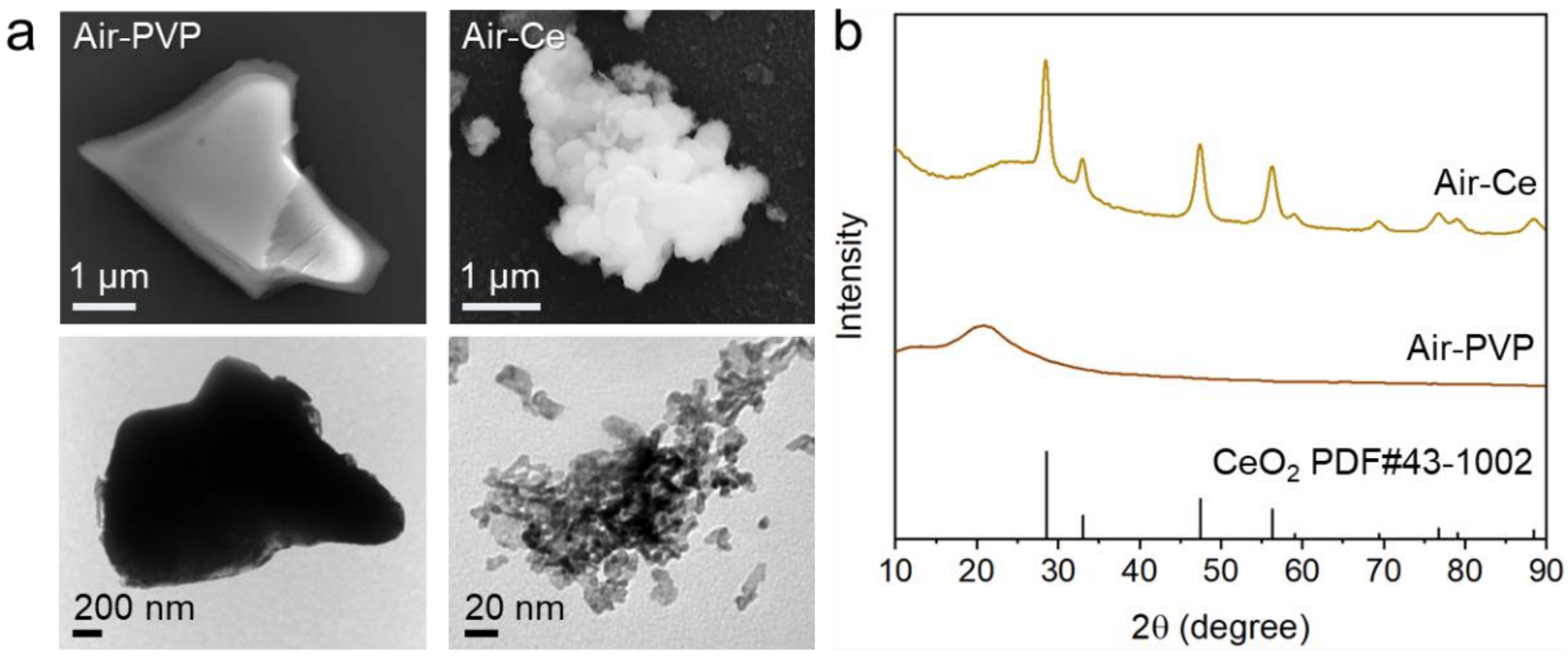

Figure S17. (a) SEM (top) and TEM (bottom) images of Air-PVP and Air-Ce, along with (b) their corresponding XRD patterns.

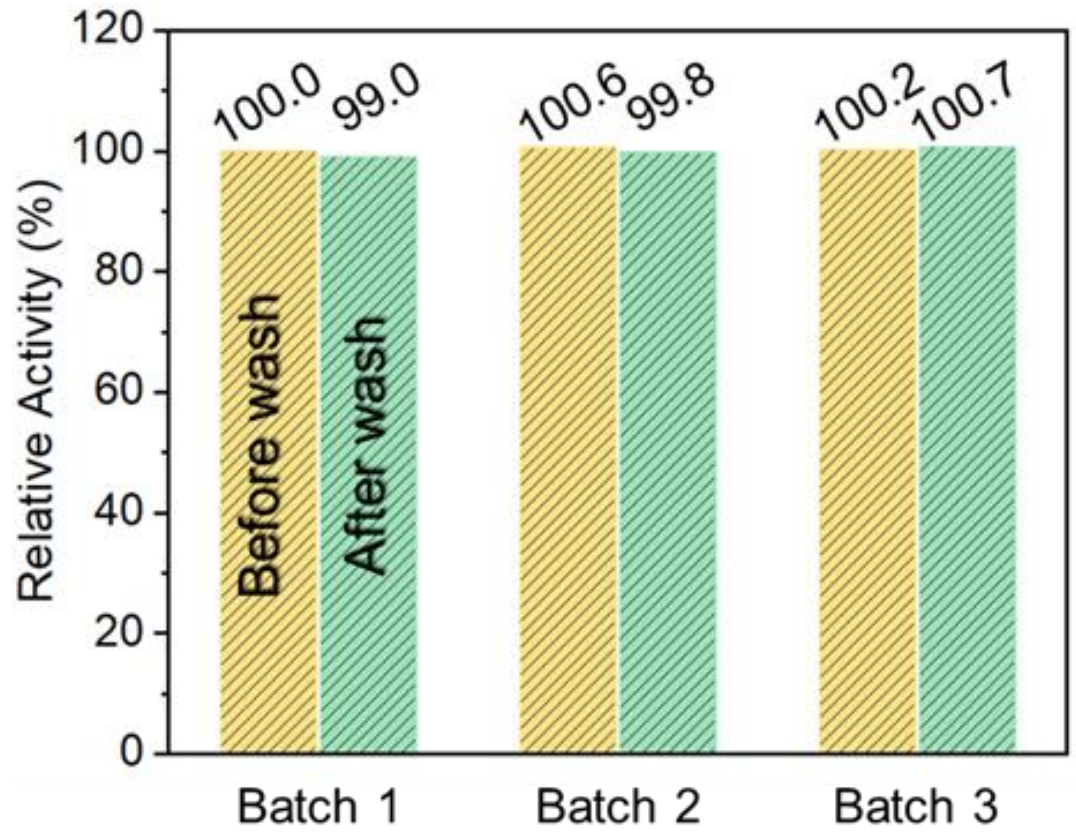

Figure S18. RhB degradation activity of Air-450 across three different batches before and after washing. The activity of the first batch before washing was set as 100%. The catalyst was purified by dispersing it in deionized water, sonicating for 5 min, and centrifuging at 12,000 rpm for 5 min. After removing the supernatant, the washed sample was dried at 60 °C overnight.

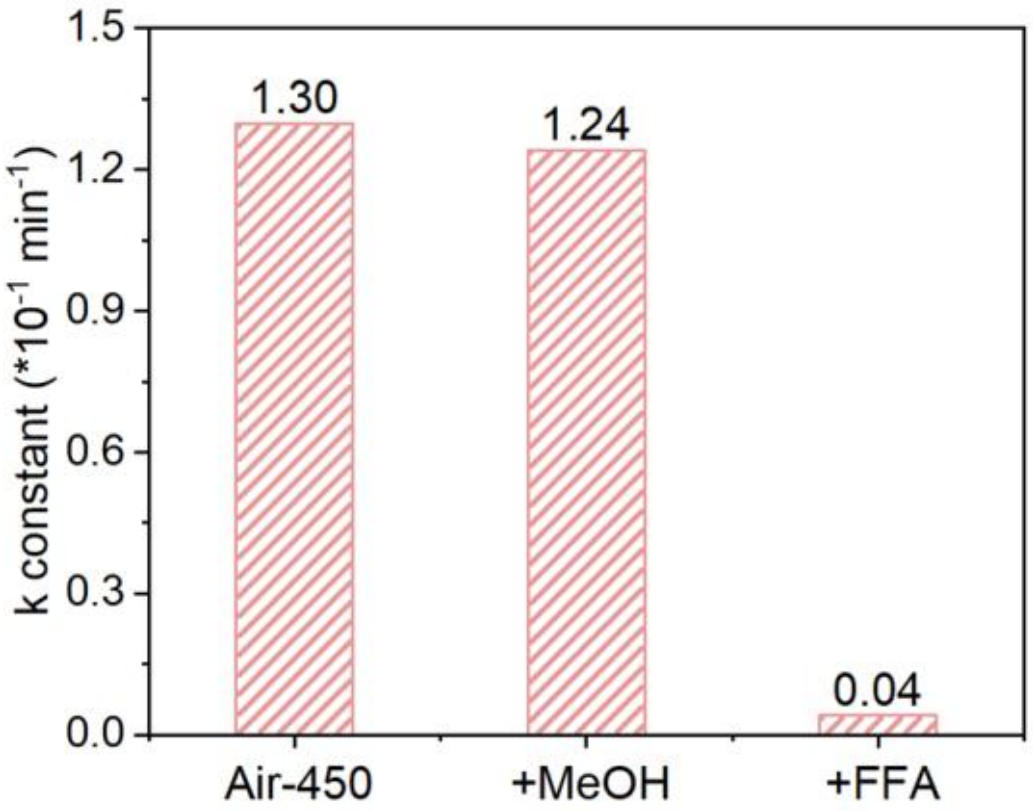

Figure S19. Comparison of RhB degradation rate constant over Air-450 in the presence of MeOH (OH radical quenchers) and FFA ($^1O_2$ quenchers).

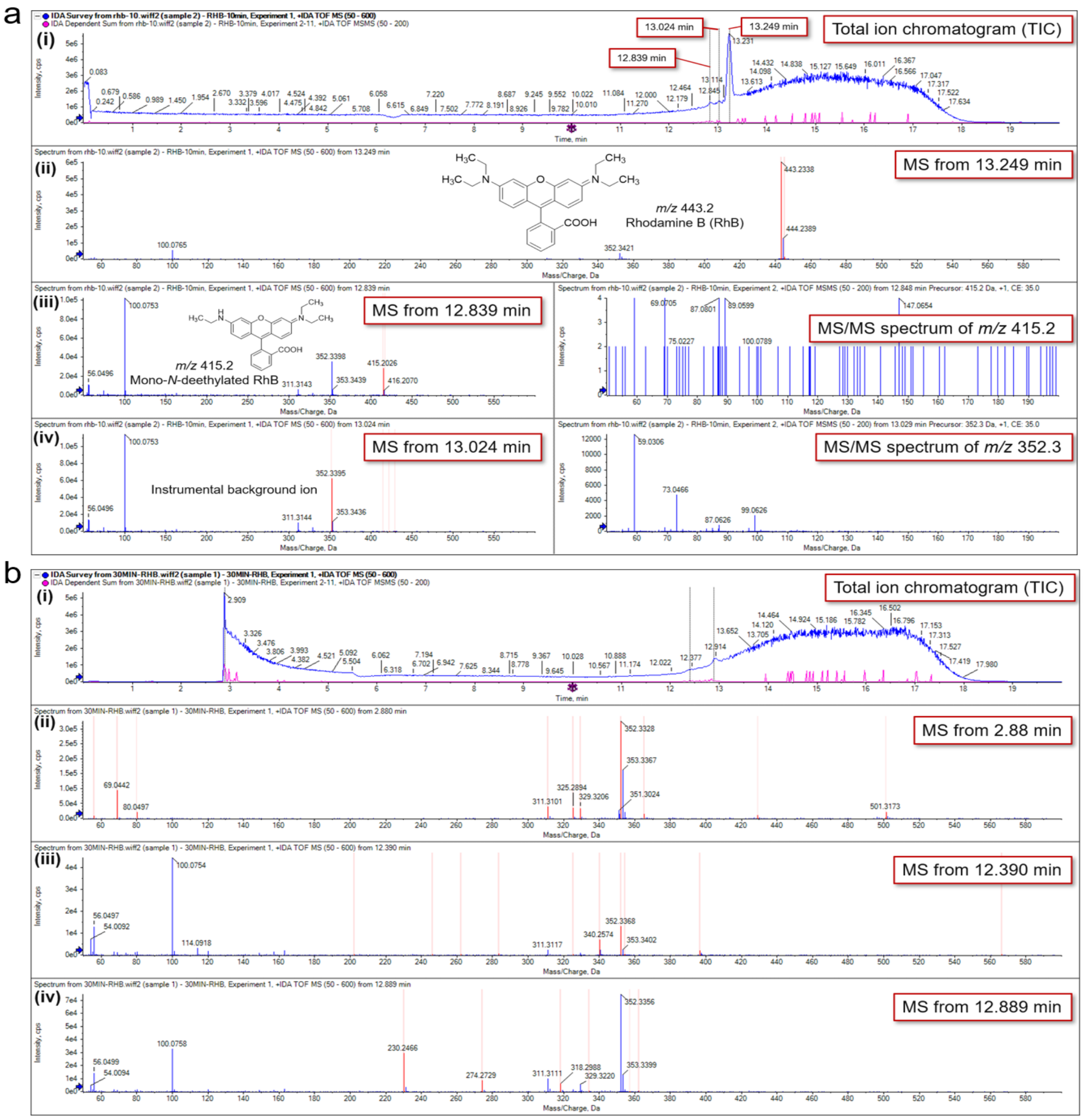

Figure S20. HPLC-MS analysis of RhB degradation products for Air-450 after (a) 10 min and (b) 30 min of reaction.

The RhB degradation products for Air-450 were analyzed by HPLC-MS. The supernatant was collected after 10 min and 30 min of reaction. At 10 min **(Figure S20a(i))**, a major peak with a retention time of 13.249 min and m/z = 443.2 was assigned to RhB[1] **(Figure S20a(ii))**, while minor peaks at retention time of 12.893 min and 13.024 min correspond to the mono-N-deethylated intermediate[2] (m/z = 415.2026, **Figure S20a(iii)**) and instrument-related contaminants[3] (m/z = 352.3395, **Figure S20a(iv)**), respectively. After 30 min **(Figure S20b(i))**, no RhB or its related mono-N-deethylated intermediate were detected, with instrument-related contaminants remained **(Figure S20b(ii)-(iv))**. All detected HPLC-MS peaks were carefully examined, and none could be assigned to brominated RhB-derived products. Furthermore, no characteristic $^{79}Br/^{81}Br$ isotope patterns[4] (e.g., M:M+2 ≈ 1:1 for monobrominated species) were observed in the full-scan mass spectra. These findings suggest that RhB degradation proceeds via $^1O_2$-mediated oxidation rather than oxidative bromination by HOBr.

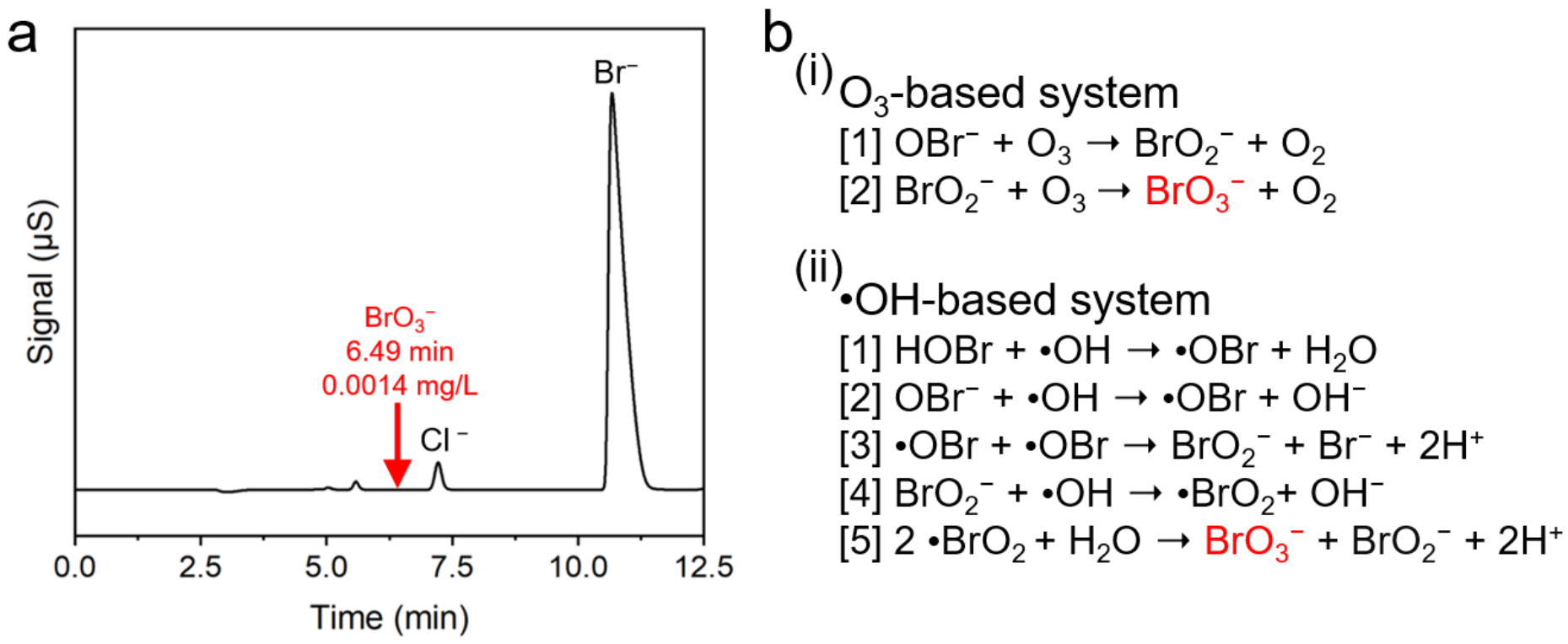

Figure S21. (a) Ion chromatogram of the supernatant after reaction for the Air-450 + H_2O_2 + KBr system. Proposed BrO_3^- formation pathways in (b) (i) O_3-based[5] and (ii) OH radicals-based[5] systems via sequential oxidation of $HOBr/OBr^-$ to BrO_3^-.

To assess potential BrO_3^- formation in our system, the catalyst was removed by centrifugation after 30 min of RhB degradation, and the BrO_3^- concentration in the supernatant was analyzed by ion chromatography (IC). As shown in **Figure S21a**, the BrO_3^- concentration was below the IC detection limit (0.002 mg/L), which is well within the World Health Organization (WHO) guideline for drinking water quality (0.01 mg/L). This negligible BrO_3^- formation, despite the presence of $HOBr/OBr^-$, is rationalized by the distinct reaction pathways in our system, in contrast to conventional ozone (O_3)-based or OH radicals-based oxidation processes. In O_3-based system **(Figure S21b(i))**[5], BrO_3^- formation is a well-documented concern, where $HOBr/OBr^-$ serves as a requisite intermediate that can be sequentially oxidized to BrO_2^- and then BrO_3^- by O_3. In OH radicals -based system **(Figure S21b(ii))**[5], BrO_3^- formation proceeds through a radical-mediated pathway. $HOBr/OBr^-$ reacts with OH radicals to form OBr radicals. These OBr radicals undergo self-disproportionation to yield BrO_2^- and Br^-. In the absence of O_3, BrO_2^- can be further oxidized by OH radicals via a single-electron transfer to generate BrO_2 radicals. Subsequently, two BrO_2 radicals undergo disproportionation to produce BrO_3^-. The absence of O_3 and the negligible production of OH radicals in our system may thus effectively eliminate the formation of BrO_3^-. Collectively, these factors should account for the undetectable BrO_3^- levels observed under our reaction conditions.

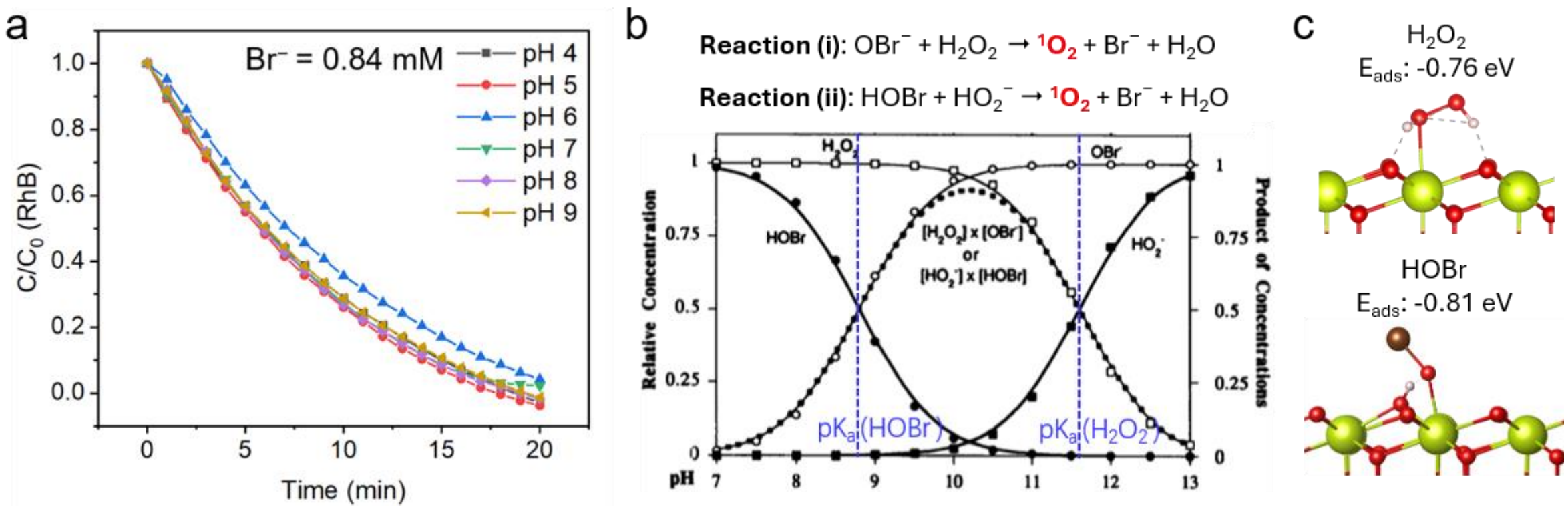

Figure S22. (a) Effect of pH on RhB degradation over Air-450 (Conditions: [RhB] = 20 ppm, [Air-450] = 0.2 g/L, [H_2O_2] = 1 mM, [Br^-] = 0.84 mM, [ion] = 1 mM). (b) The reaction between HOBr/OBr^- and H_2O_2/HO_2^- and their relative proportion as a function of pH. Reproduced with permission from ref [6]. Copyright 1997 Elsevier. (c) Adsorption energies and configurations of H_2O_2 and HOBr on the CeO_2 (111) surface.

According to the literature[6], the oxidation of Br^- to HOBr is influenced by pH, as the equilibrium between HOBr and its deprotonated form, OBr^-, is pH-dependent **(Figure S22b)**. Consequently, with a pK_a of 8.63 (HOBr/OBr^-), the formation of HOBr is favored under acidic to neutral conditions (peaked at pH = 7), while higher pH conditions shift the balance toward the OBr^- species. To generate $^1O_2$, these HOBr/OBr^- species must further react with H_2O_2/HO_2^- (pK_a: 11.65) via the reactions either between HOBr and HO_2^- (reaction (i)) or between OBr^- and H_2O_2 (reaction (ii)). The optimal production of $^1O_2$ via this uncatalyzed approach is thus expected to peak at approximately pH = 10, with reduced $^1O_2$ generation at lower pH values **(Figure S22b)**. However, a significant amount of $^1O_2$ production was observed in the Air-450 sample, which was found to be the key species degrading RhB at neutral. This was confirmed by the use of $^1O_2$ scavenger, FFA **(Figure 2e)**, as well as by the absence of brominated by-products from oxidative bromination by HOBr **(Figure S20)**. The degradation of RhB was even found to be comparable across a wider pH range from 4 to 9 **(Figure S22a)**. Although the underlying mechanism is still under investigation, we believe that the deviation of $^1O_2$ production from the diagram is closely associated with the introduction of the CeO_2 (111) surface, which promotes the dissociative adsorption of HOBr, in contrast to the associative adsorption of H_2O_2 **(Figure S22c)**. Accordingly, the generated HOBr may thus re-adsorbed onto the (111) surface and subsequently deprotonate to OBr^-, which then undergoes reaction (ii) to yield $^1O_2$.

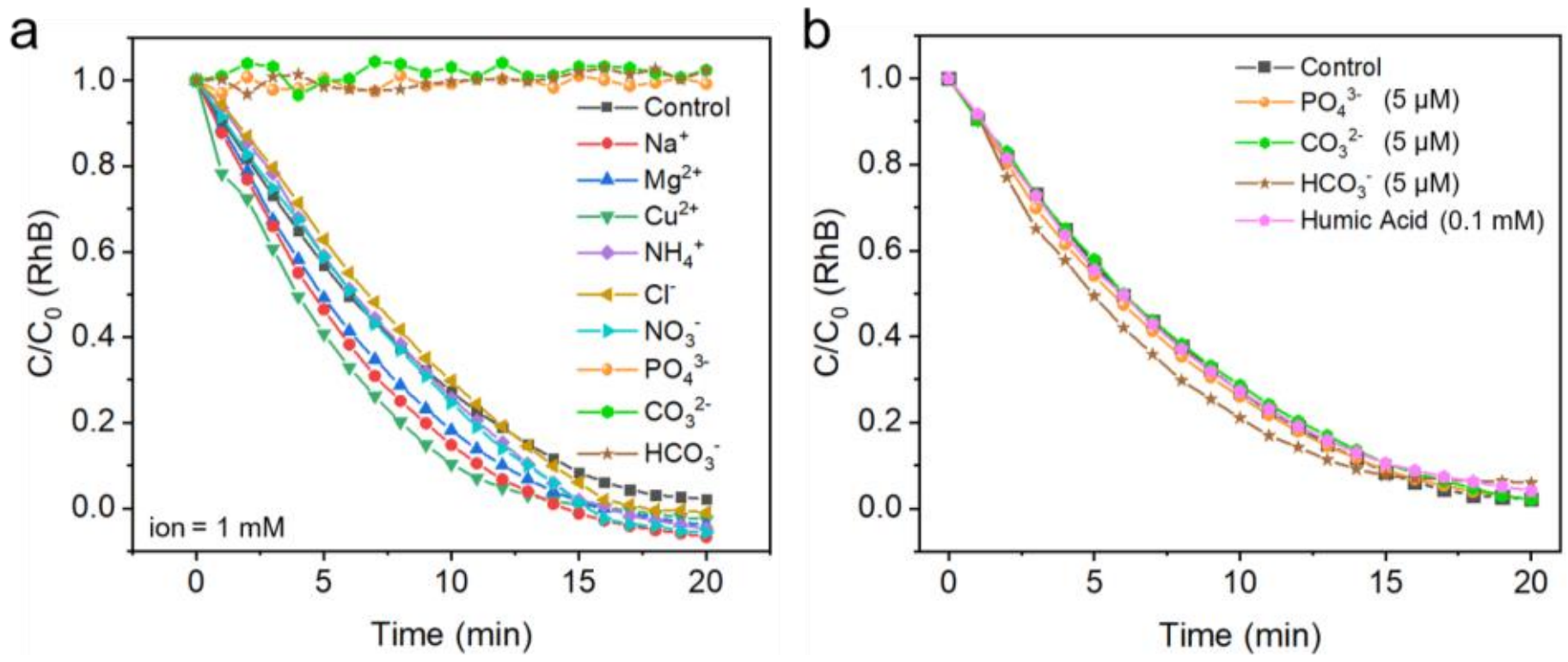

Figure S23. Effect of (a) common ions (each at 1 mM) and (b) low-concentration ions (5 μM) and humic acid (0.1 mM) on RhB degradation over Air-450 (Conditions: [RhB] = 20 ppm, [Air-450] = 0.2 g/L, [H_2O_2] = 1 mM, [Br^-] = 0.84 mM).

The effect of common ions (1mM) on RhB removal was evaluated. As shown in **Figure 23a**, bicarbonate, carbonate, and phosphate anions exhibited a strong suppression of RhB degradation compared to other common ions. This strong interference is attributed to their high affinity for surface cations, which leads to the formation of surface complexes on metal oxides and subsequently blocks active sites and hinders the reaction. For instance, surfactants commonly used for particle surface coordination often rely on terminal carbonate (or carboxylate) groups for binding [7]. Similarly, the strong interaction between phosphate and Ce cations is well documented in the literature [8]. Despite this interference, our catalytic system is designed for the tertiary treatment of recalcitrant organics, ARGs, and ARBs, where interference from these specific inorganic buffers can be effectively managed using standard wastewater treatment plant (WWTP) pre-treatment protocols. In municipal wastewater treatment, it is standard practice to employ chemical coagulation or precipitation (e.g., using trace Al^{3+} or Fe^{3+} salts) immediately prior to advanced oxidation units. This standard pre-treatment step effectively precipitates phosphates and allows for mild alkalinity adjustment or softening (reducing carbonate/bicarbonate loads), thereby minimizing interference and preserving the activity of our CeO_2 catalyst, with negligible inhibition at 5 μM **(Figure S23b)**. As for the potential interference of natural organic matter (NOM), they are known to be majority removed after the influent undergoes rigorous primary and secondary (biological) treatments. Due to their macromolecular nature, hydrophobicity, and surface charge characteristics, humic acid and other NOM are highly susceptible to biomass adsorption, flocculation, and trapping within the activated sludge. Consequently, by the time the effluent exits the secondary clarifier, the concentration of NOM and other background matrices has been drastically reduced to a very low level, typically around 4 mg/L as dissolved organic carbon (DOC)[9]. Since our CeO_2 catalyst is specifically designed for deployment as a polishing AOP stage during tertiary treatment, we examined RhB degradation over Air-450 in the presence of 0.1 mM humic acid (~6 mg/L dissolved organic carbon (DOC)). As shown in **Figure S23b**, the presence of humic acid had a negligible effect on RhB removal.

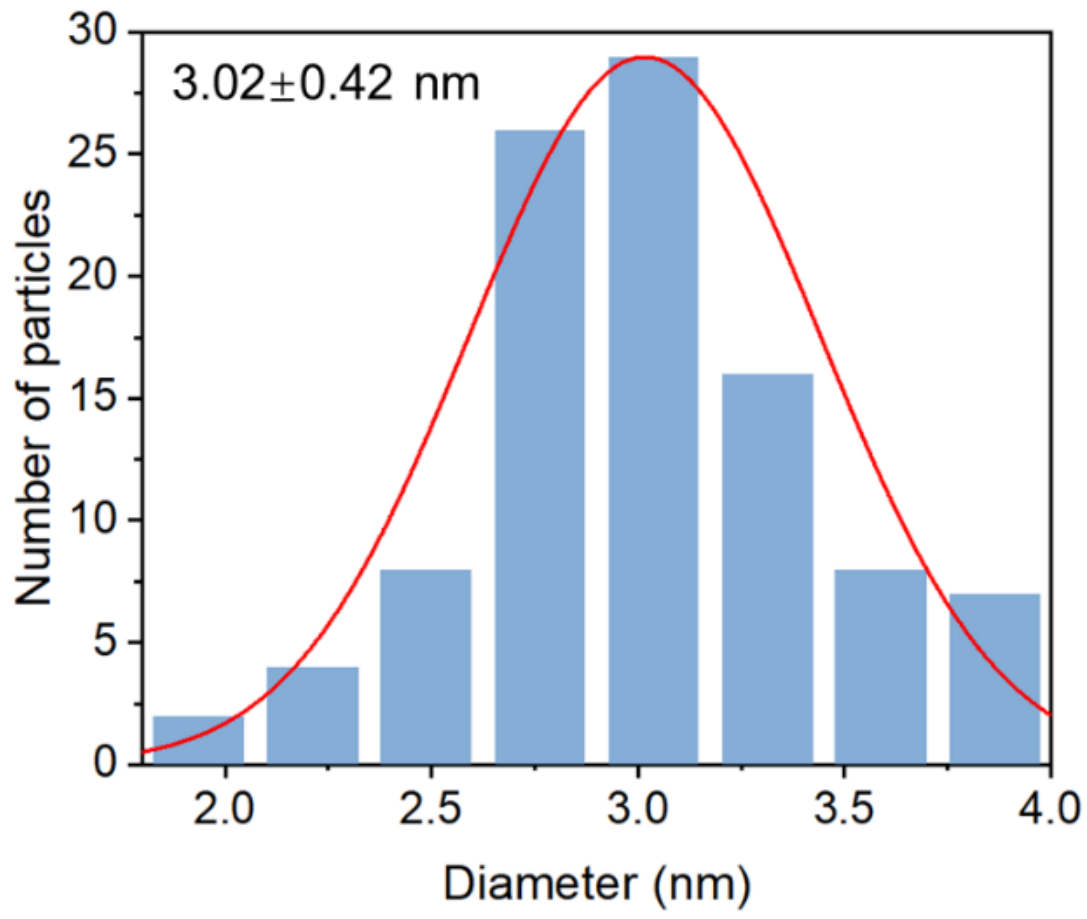

Figure S24. Particle size distribution of N_2-450 (100 particles were counted for the sample).

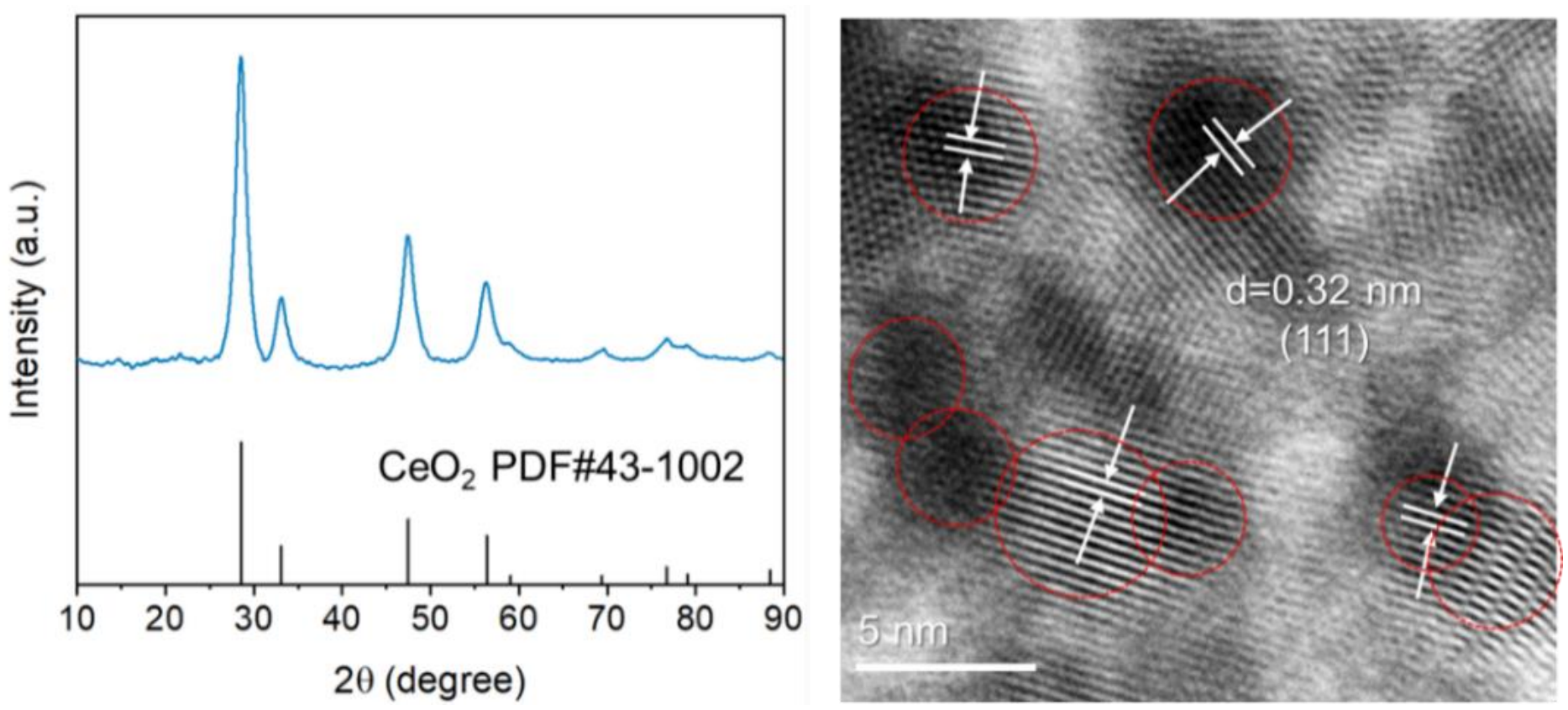

Figure S25. XRD pattern (left) and HRTEM image (right) of N_2-450.

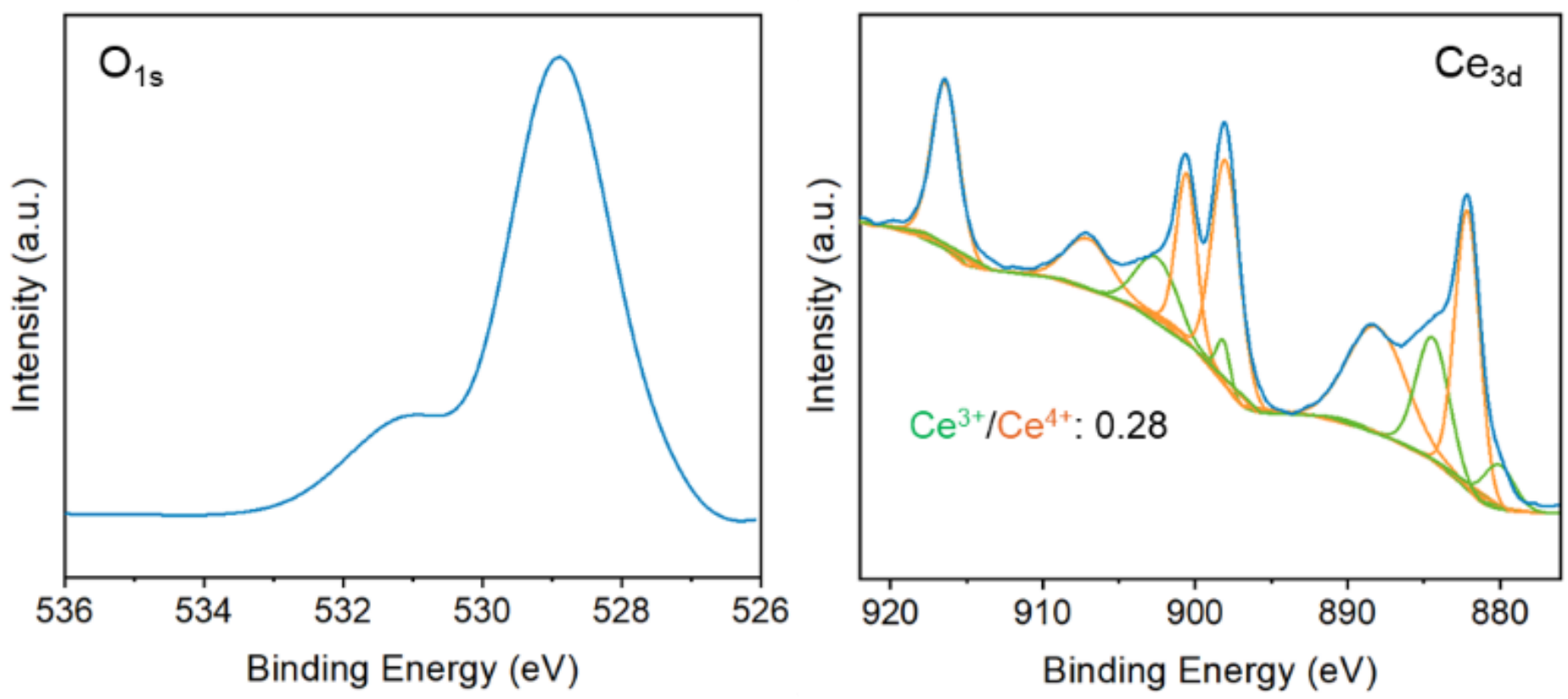

Figure S26. XPS O_{1s} and Ce_{3d} spectra and the calculated Ce^{3+}/Ce^{4+} ratio for N_2-450.

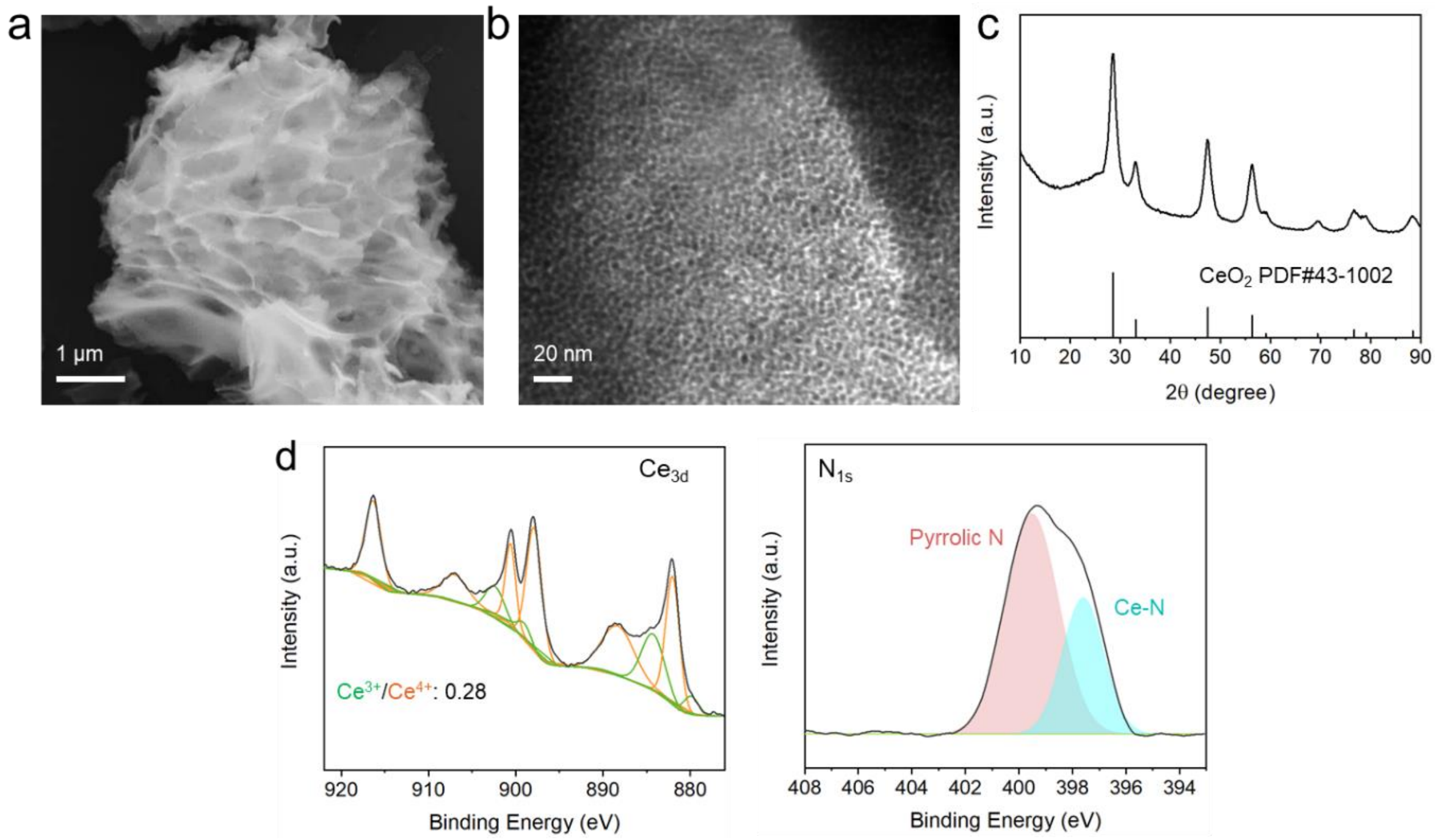

Figure S27. (a) SEM, (b) TEM, (c) XRD, and (d) XPS results for the sample prepared by calcining the pre-mixed $Ce(NO_3)_3$ and PVP at 450°C under argon.

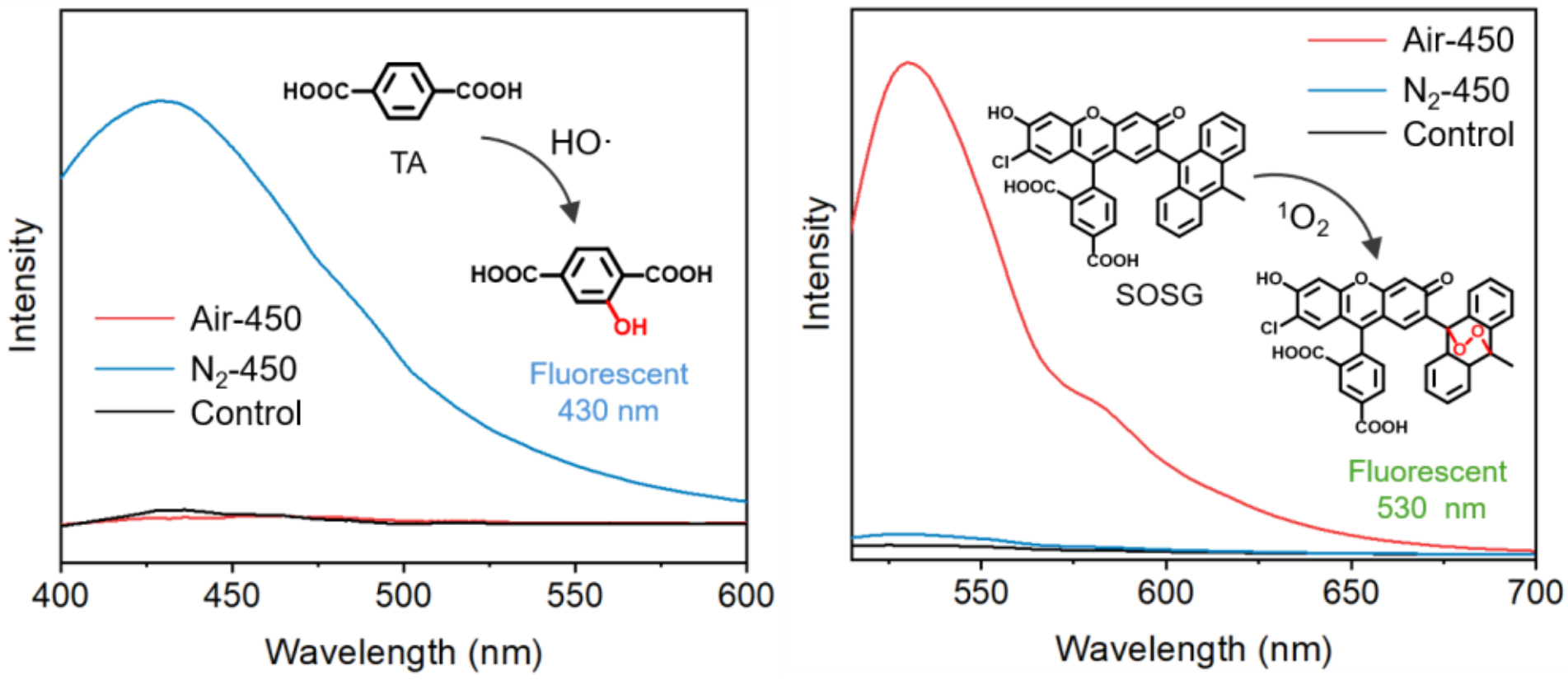

Figure S28. Fluorescence detection of ROS using TA for OH radicals (left) and SOSG for ${}^1O_2$ (right) over Air-450 and N_2-450.

For OH radical detection (left), terephthalic acid (TA) was used, as its reaction with OH radicals forms the fluorescent product 2-hydroxyterephthalic acid (TAOH, emission at 430 nm). Indeed, N_2-450 produced a strong fluorescence signal, while Air-450 showed only a negligible signal, same as the control. This agrees with the EPR results that OH radicals are exclusively generated by N_2-450. For ${}^1O_2$ detection (right), singlet oxygen sensor green (SOSG) was employed, as its reaction with ${}^1O_2$ yields a characteristic fluorescence at 530 nm. As expected, Air-450 displayed a strong fluorescence signal, whereas N_2-450 showed negligible fluorescence, again matching the EPR results and confirming that ${}^1O_2$ is exclusively produced by Air-450. Collectively, these complementary fluorescence assays corroborate the EPR findings that Air-450 and N_2-450 exclusively follow nonradical and radical pathways, respectively.

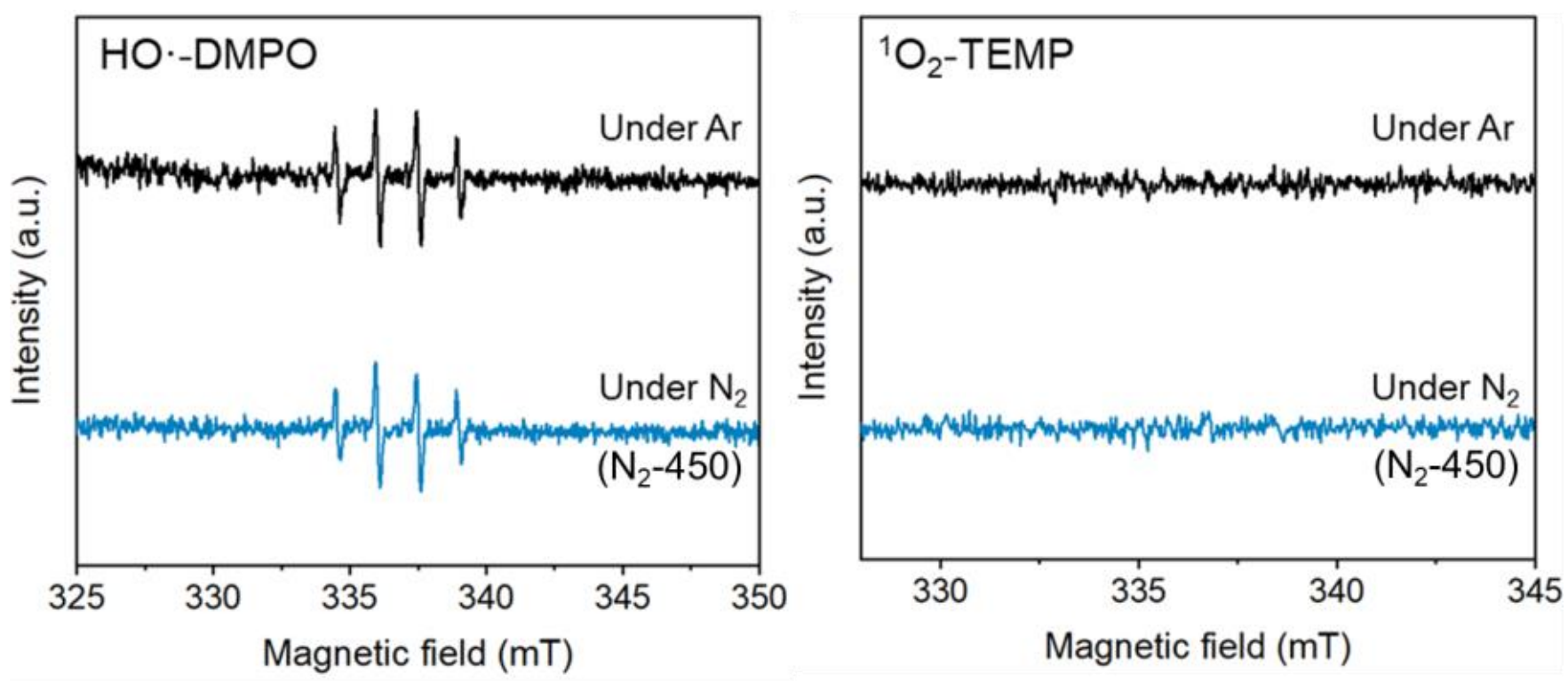

Figure S29. Monitoring the production of OH radicals (left) and ${}^1O_2$ (right) at reaction time of 30 minutes over the samples prepared at 450°C under N_2 (i.e., N_2-450) and argon.

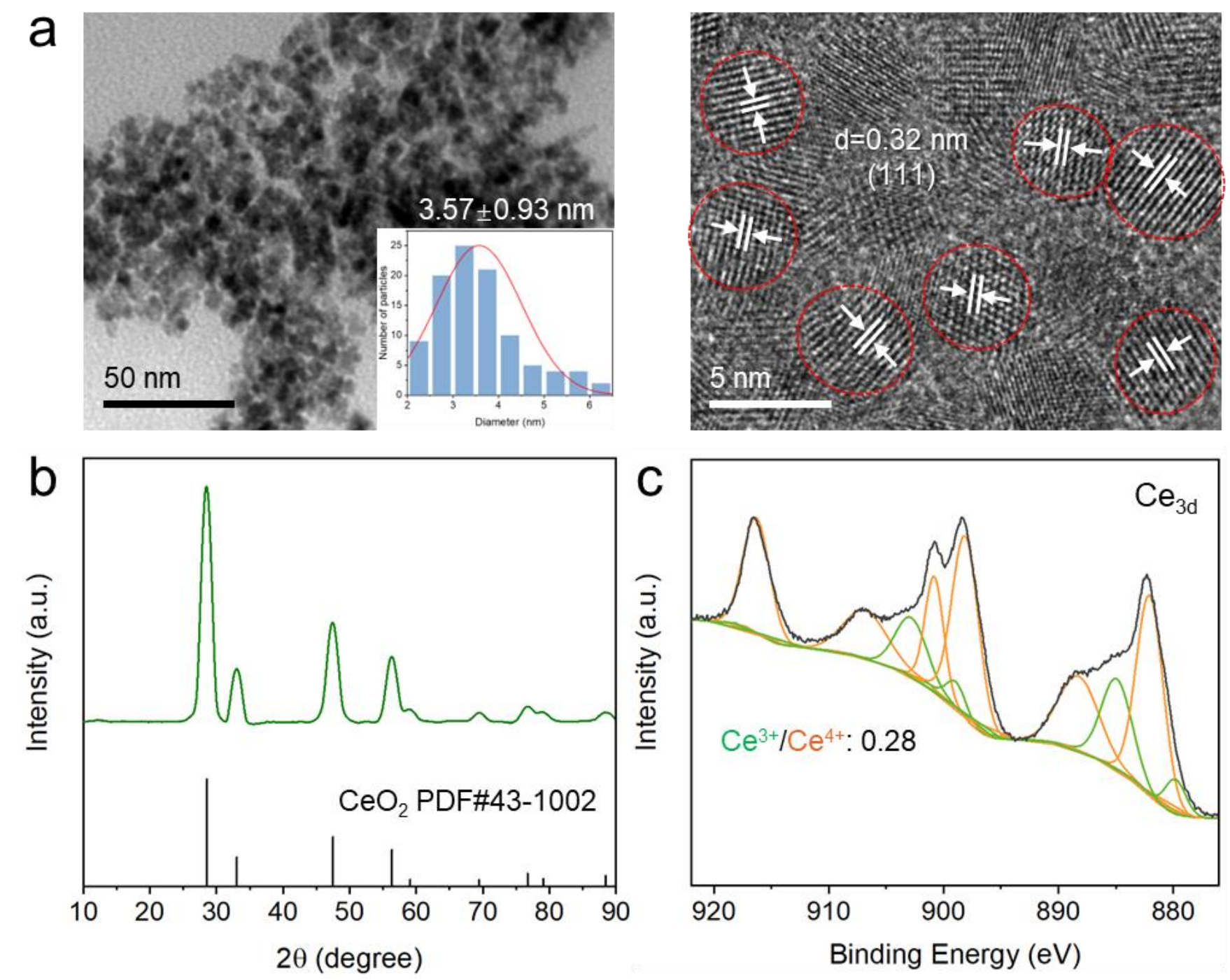

Figure S30. (a) TEM/HRTEM images, (b) XRD pattern, and (c) XPS spectrum for ty-Ce.

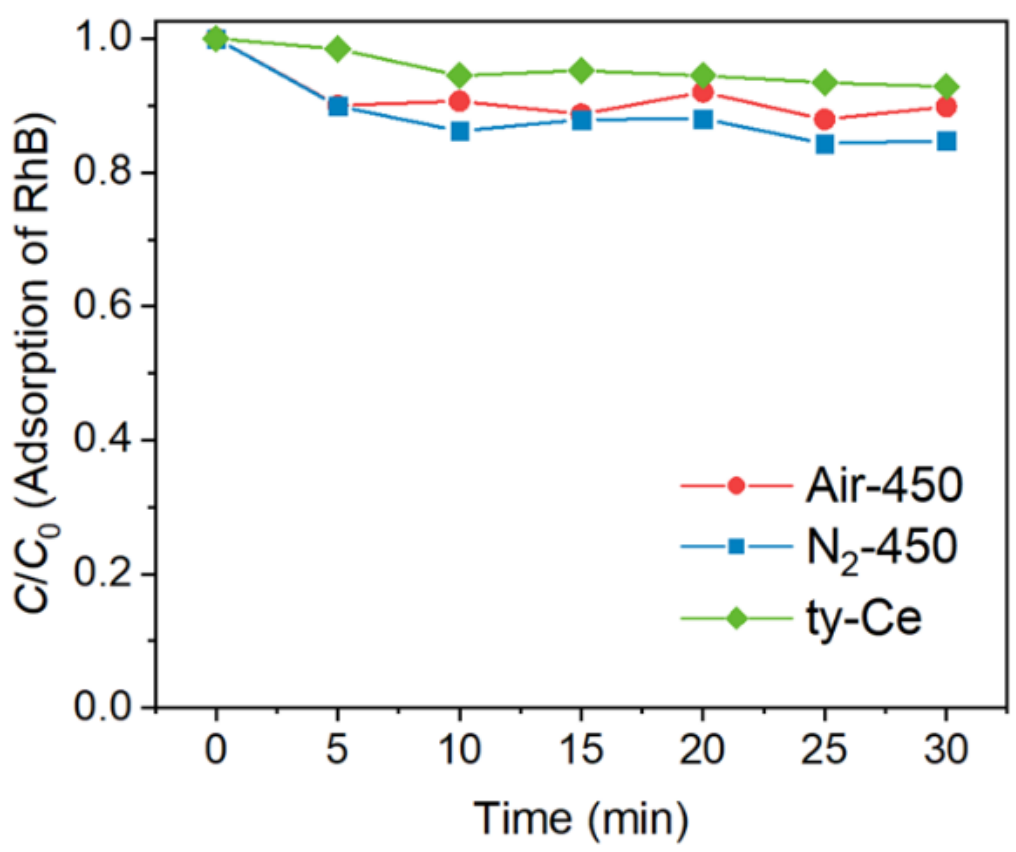

Figure S31. Adsorption of RhB on samples for 30 mins.

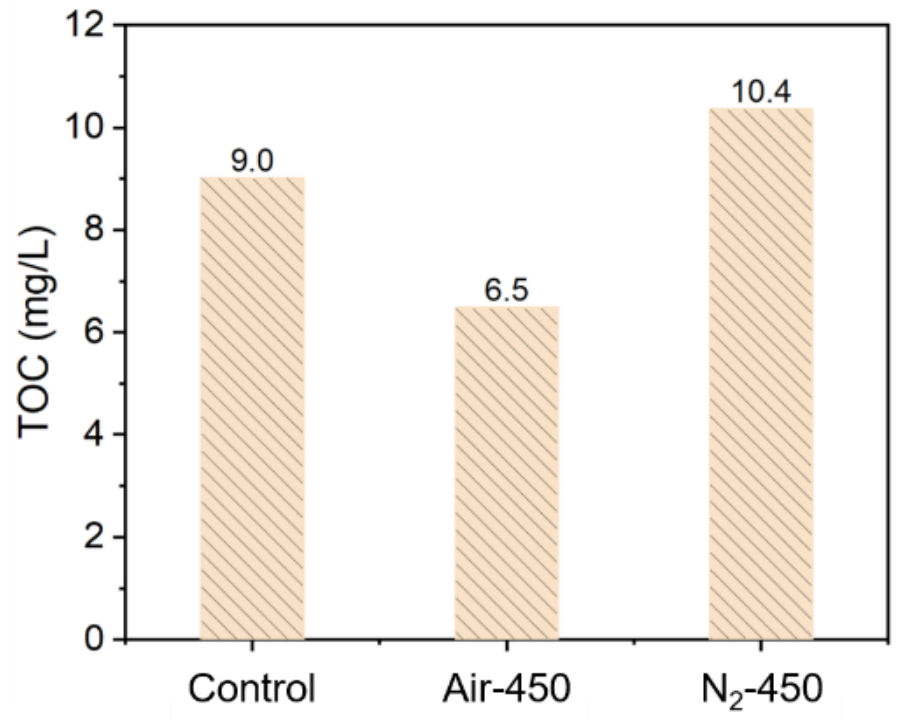

Figure S32. TOC analysis for the supernatant of the sample after 30 min of RhB degradation.

Regarding mineralization, we measured the total organic carbon (TOC) removal after 30 min of reaction under the same conditions used for RhB degradation. The TOC concentration decreased from 9.0 mg/L in the control (without catalyst) to 6.5 mg/L in the supernatant after treatment with Air-450 + H_2O_2 + KBr, corresponding to a TOC removal of approximately 28%. In contrast, treatment with N_2-450 under identical conditions resulted in a slight increase in TOC to 10.4 mg/L (cf. control), likely due to its poor catalytic activity and residue catalyst particles in the supernatant. The moderate TOC removal observed for Air-450 indicates that while RhB is efficiently degraded (as evidenced by the disappearance of its characteristic UV-vis absorption, **Figure 3h(i)**), a significant portion of the organic carbon is converted to intermediates yet to be fully mineralized to CO_2.

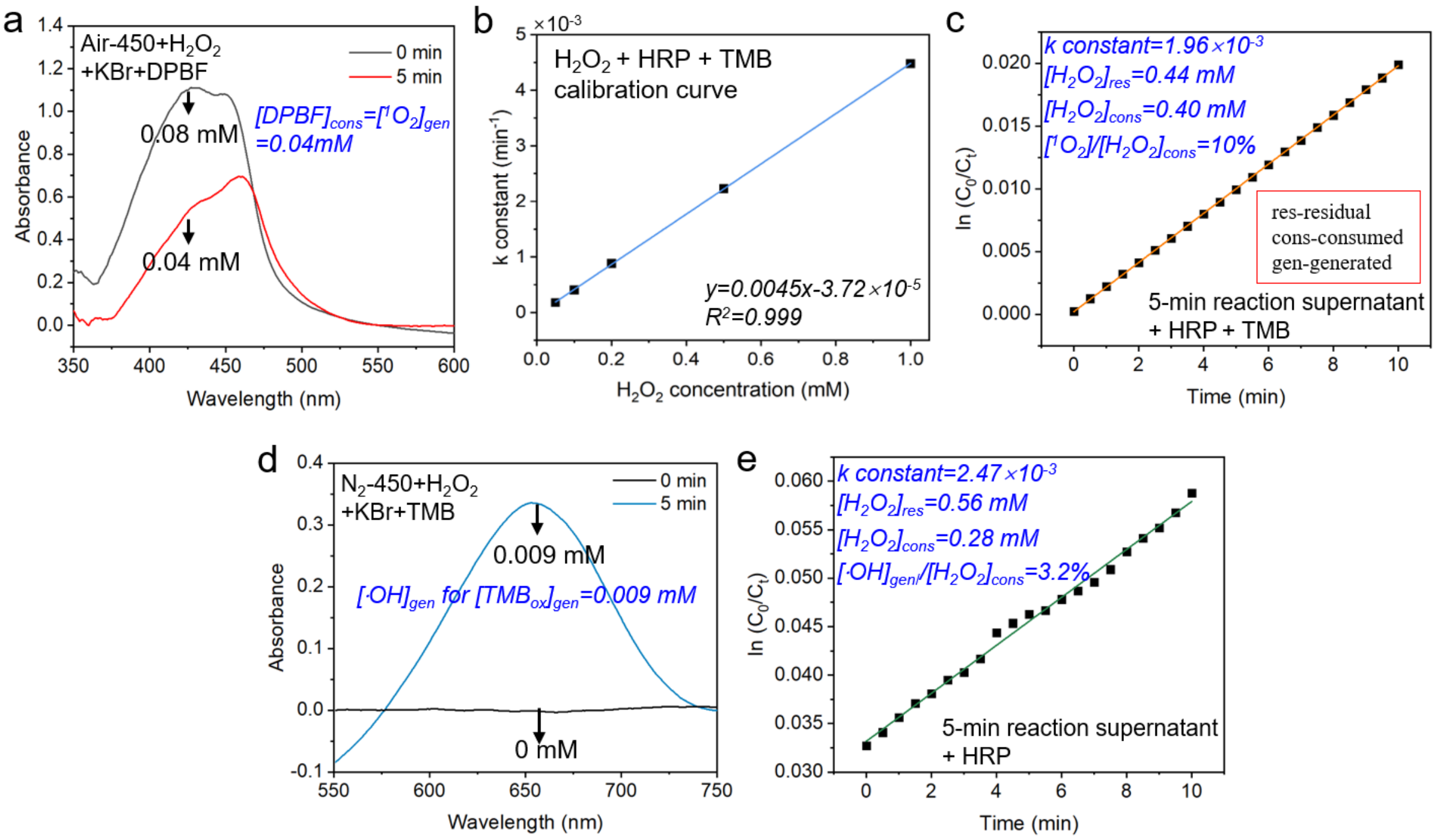

Figure S33. (a) Quantification of $^1O_2$ produced by the Air-450 + H_2O_2 + KBr system using DPBF. (b) Calibration curve for H_2O_2 concentration determined by the HRP assay. (c) Residual H_2O_2 concentration in the Air-450 system after the reaction measured by the HRP assay. (d) TMB oxidation by OH radicals in the N_2-450 + H_2O_2 + KBr system. (e) Residual H_2O_2 concentration in the N_2-450 system after the reaction measured by the HRP assay.

To quantify the H_2O_2-to-$^1O_2$ conversion efficiency, $^1O_2$ production and H_2O_2 consumption were probed by DPBF (1,3-diphenylisobenzofuran) and HRP (horseradish peroxidase) assay, respectively. DPBF reacts stoichiometrically (1:1) with $^1O_2$ via irreversible oxidation, resulting in a decrease in its characteristic absorption at 425 nm[10]. Thus, DPBF bleaching directly reports $^1O_2$ yield. For Air-450, which exclusively follows the nonradical pathway, the decay of DPBF absorbance at 425 nm over 5 minutes indicated the production of approximately 0.04 mM of $^1O_2$ **(Figure S33a)**. On the other hand, the residual H_2O_2 concentration was determined using an HRP-mediated TMB (3,3',5,5'-tetramethylbenzidine) oxidation assay, in which the TMB oxidation rate by HRP is proportional to the H_2O_2 concentration **(Figure S33b)**. After 5 minutes of reaction, the catalyst was rapidly removed by membrane filtration. Subsequent addition of HRP and TMB yielded a rate constant of 1.96×10^{-3}, corresponding to a residual H_2O_2 concentration of 0.44 mM **(Figure S33c)**. This indicates that 0.40 mM H_2O_2 was consumed in the presence of Air-450 (from an initial concentration of 0.84 mM), with corresponding $^1O_2$ production of 0.04 mM, giving a Br^--mediated H_2O_2-to-$^1O_2$ conversion efficiency of 10%. The deviation of this value from the theoretical maximum of 50% can be attributed to several factors. First, not all of the HOBr produced can be converted to $^1O_2$. Second, DPBF cannot quantitatively trap all of the $^1O_2$ generated, as it reacts with a finite rate. It should be noted that direct measurement of $^1O_2$ phosphorescence at 1270 nm was unsuccessful, likely due to the weak intensity of the forbidden transition and potential interference from the catalytic

system. Third, H_2O_2 can adsorb strongly onto the catalyst surface, resulting in a lower residual concentration in the solution.

Similarly, for N_2-450, which exclusively follows the radical pathway, TMB oxidation was utilized to assess the amount of effective OH radicals. As shown in **Figure S33d,** the effective OH radicals in our catalytic system were calculated to be 0.009 mM over 5 minutes. After the reaction, the catalyst was rapidly removed by membrane filtration, followed by the addition of HRP, which oxidizes TMB, yielding a rate constant of 2.47×10^{-3} **(Figure S33e)**, corresponds to a residue H_2O_2 concentration of 0.56 mM. This indicates that 0.28 mM H_2O_2 was consumed in the presence of N_2-450 (from an initial concentration of 0.84 mM), with only 0.009 mM OH radicals for TMB oxidation, resulting in a much lower H_2O_2 utilization of 3.2% (cf. 10% obtained for nonradical pathway for Air-450). We attribute this difference to the short-lived nature of OH radicals, which are rapidly consumed by unproductive pathways, including H_2O_2 decomposition **(Figure 3g)** and self-scavenging via chain-termination reactions **(Scheme 1b)**.

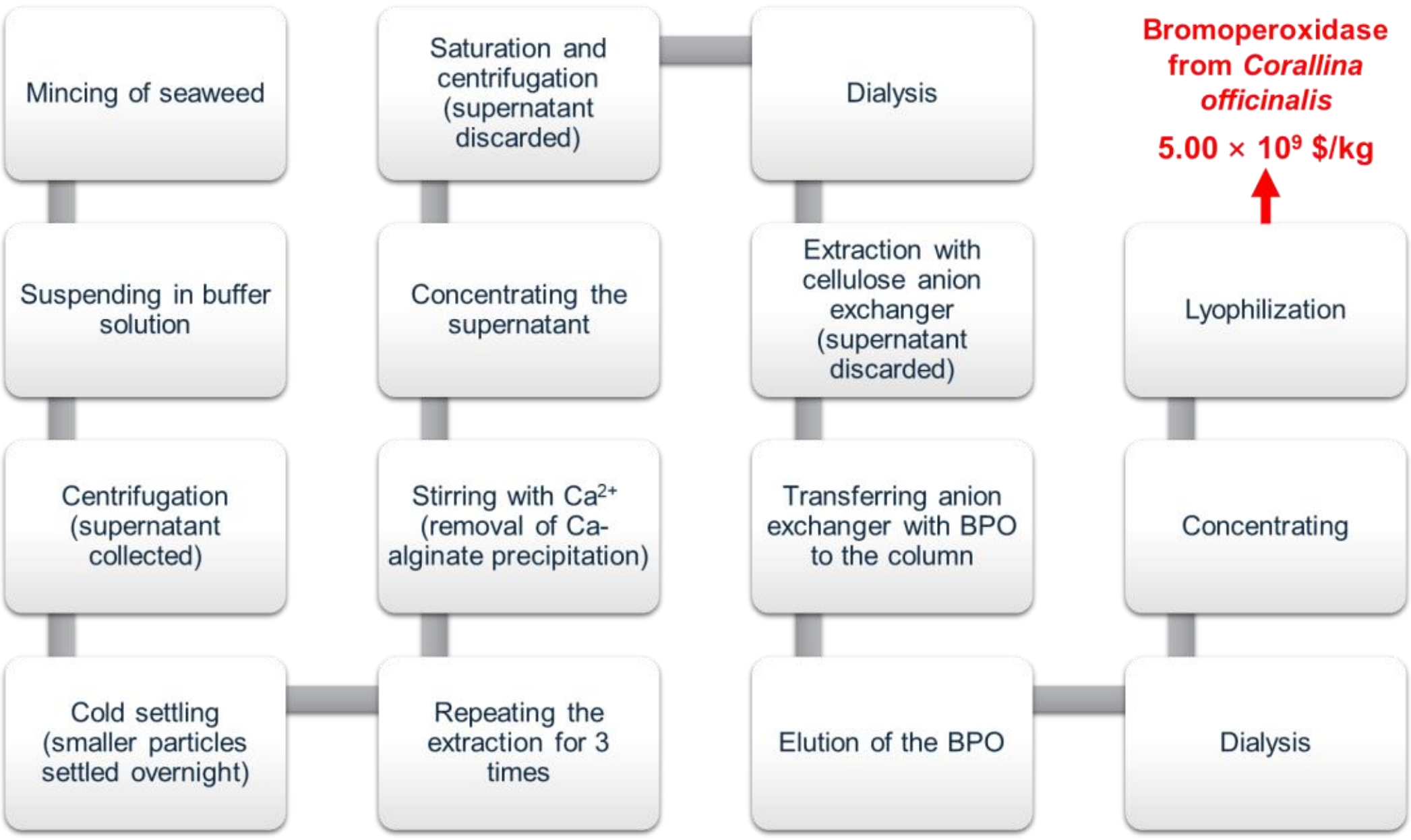

Figure S34. Schematic illustration of the production processes for BPO derived from seaweed (Corallina officinalis)[11,12].

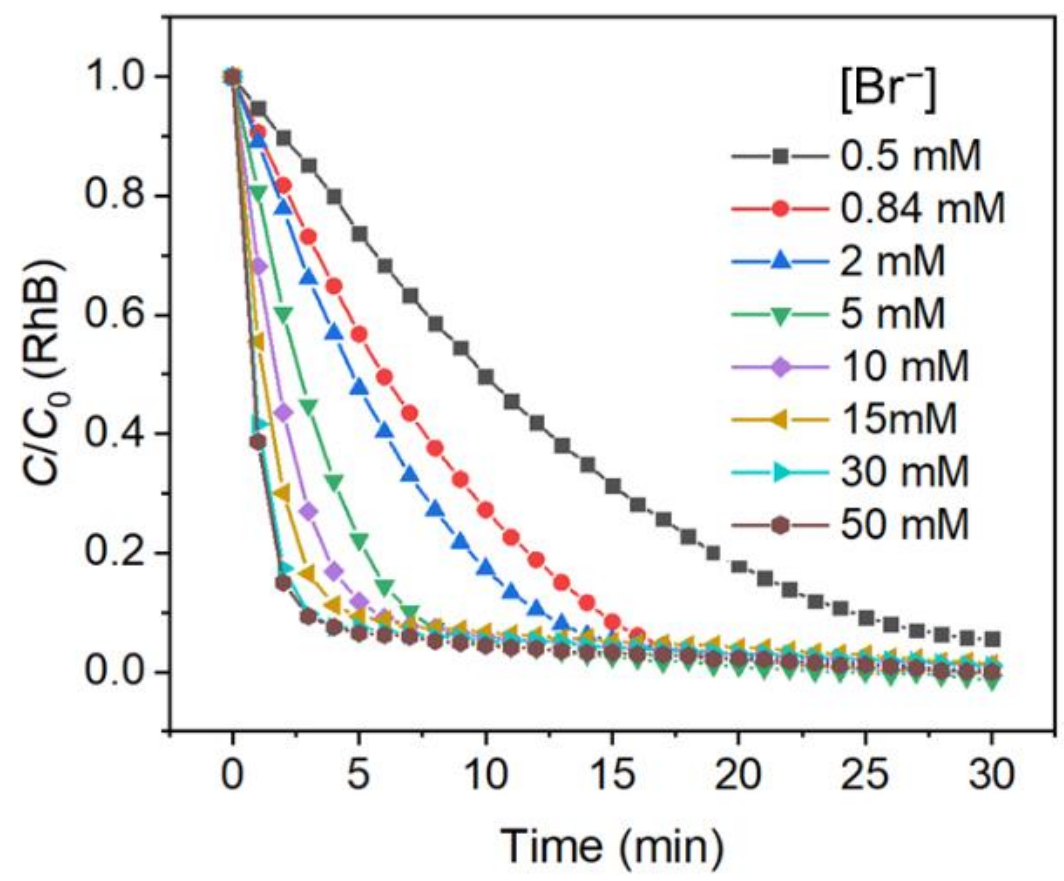

Figure S35. Effects of [Br⁻] on RhB degradation over Air-450 with [H_2O_2] fixed at 1 mM.

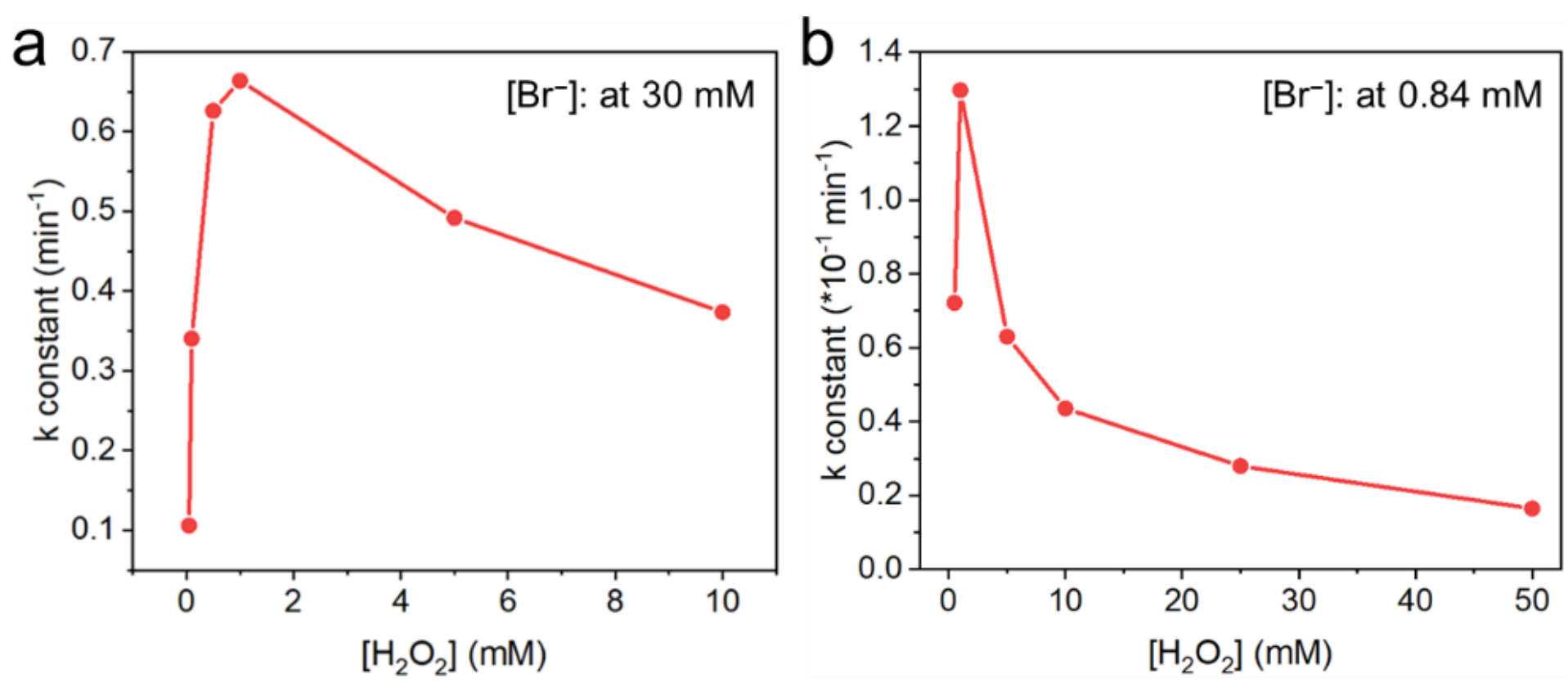

Figure S36. Effects of [H_2O_2] on RhB degradation over Air-450 with [Br⁻] fixed at (a) 30 mM and (b) 0.84 mM.

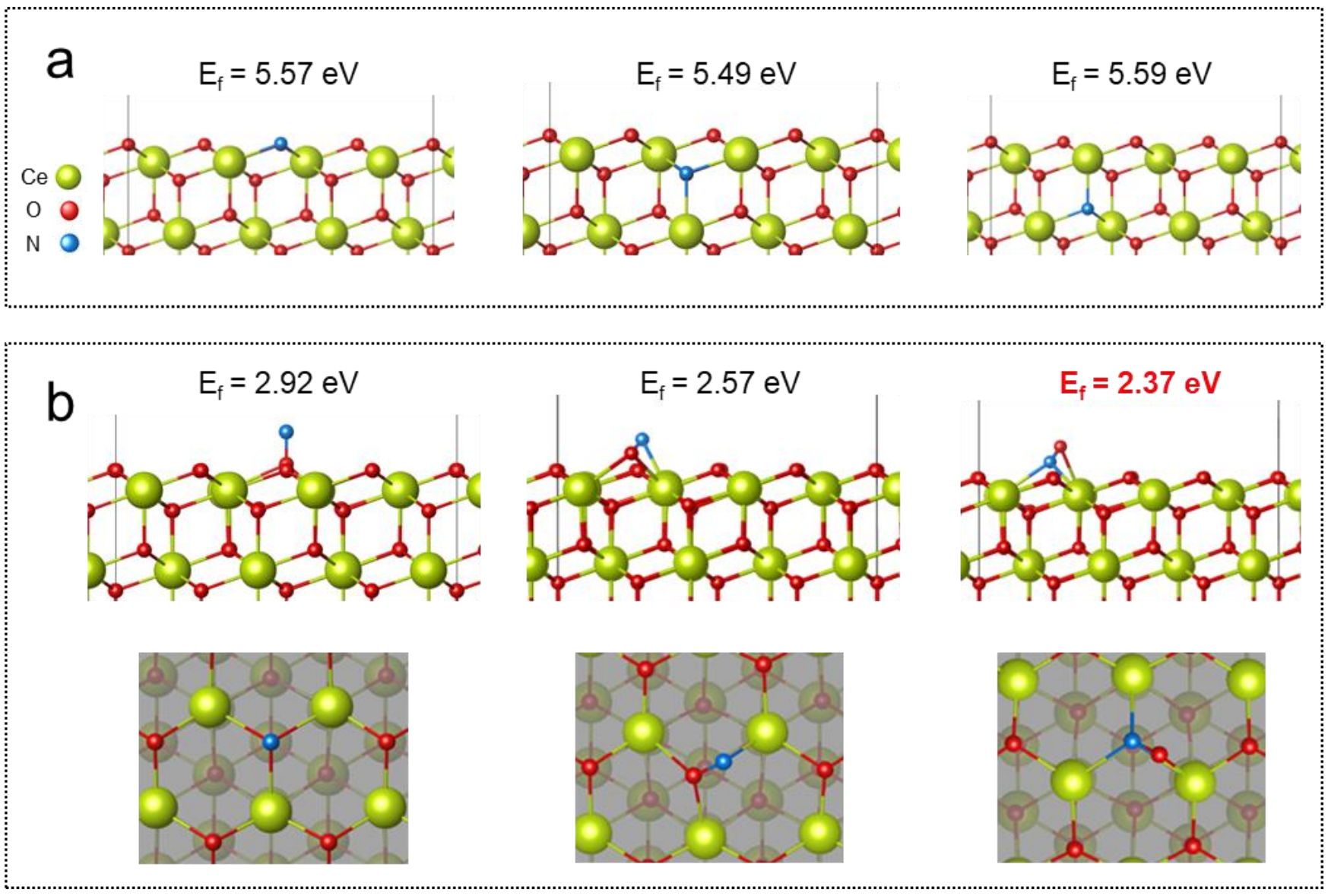

Figure S37. Calculated formation energies for (a) substitution of lattice oxygen (surface and subsurface) by nitrogen on the (111) surface and (b) introduction of nitrogen adatoms on the (111) surface.

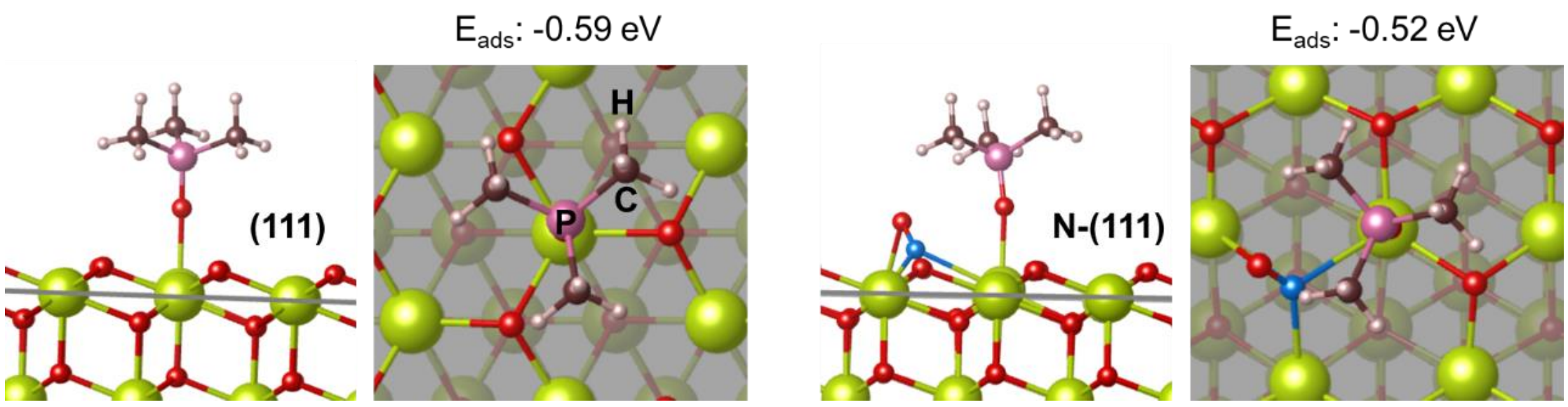

Figure S38. TMPO adsorption energies on the pristine (111) surface (left) and the N-(111) surface (right).

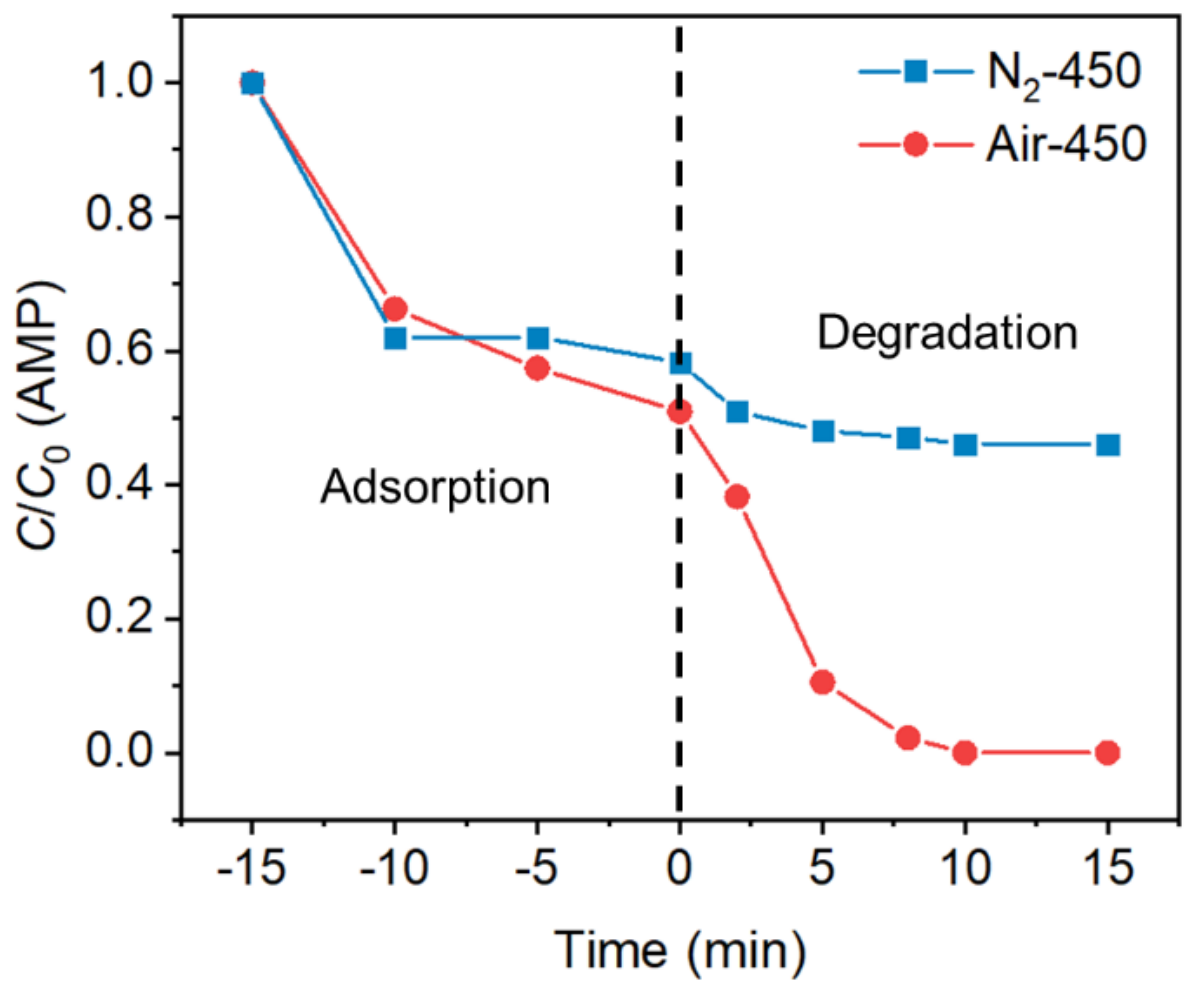

Figure S39. Adsorption of ampicillin over N_2-450 and Air-450 followed by its degradation upon H_2O_2/Br^- addition.

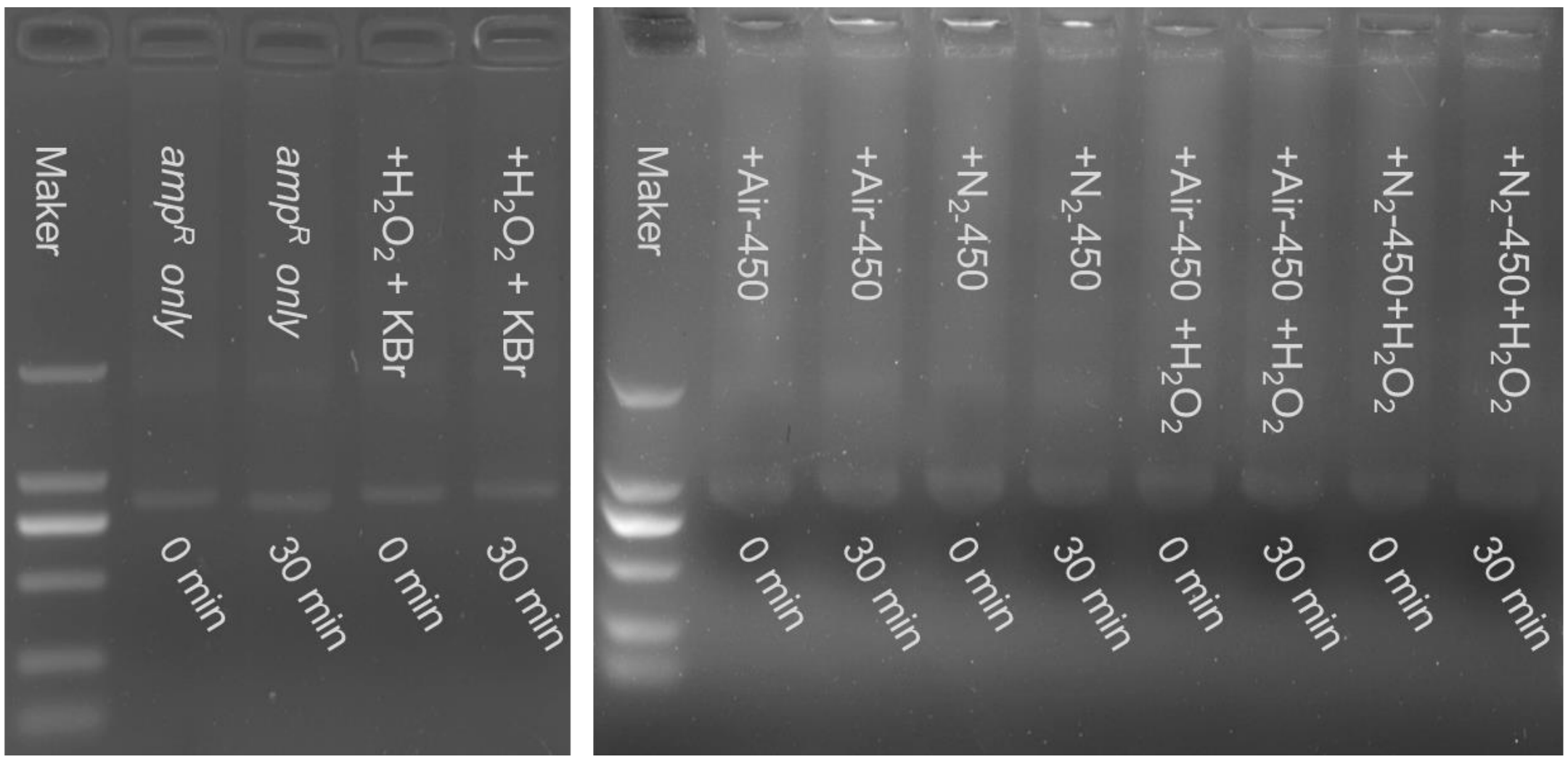

Figure S40. Agarose gel electrophoresis of amp^R gene under different conditions.

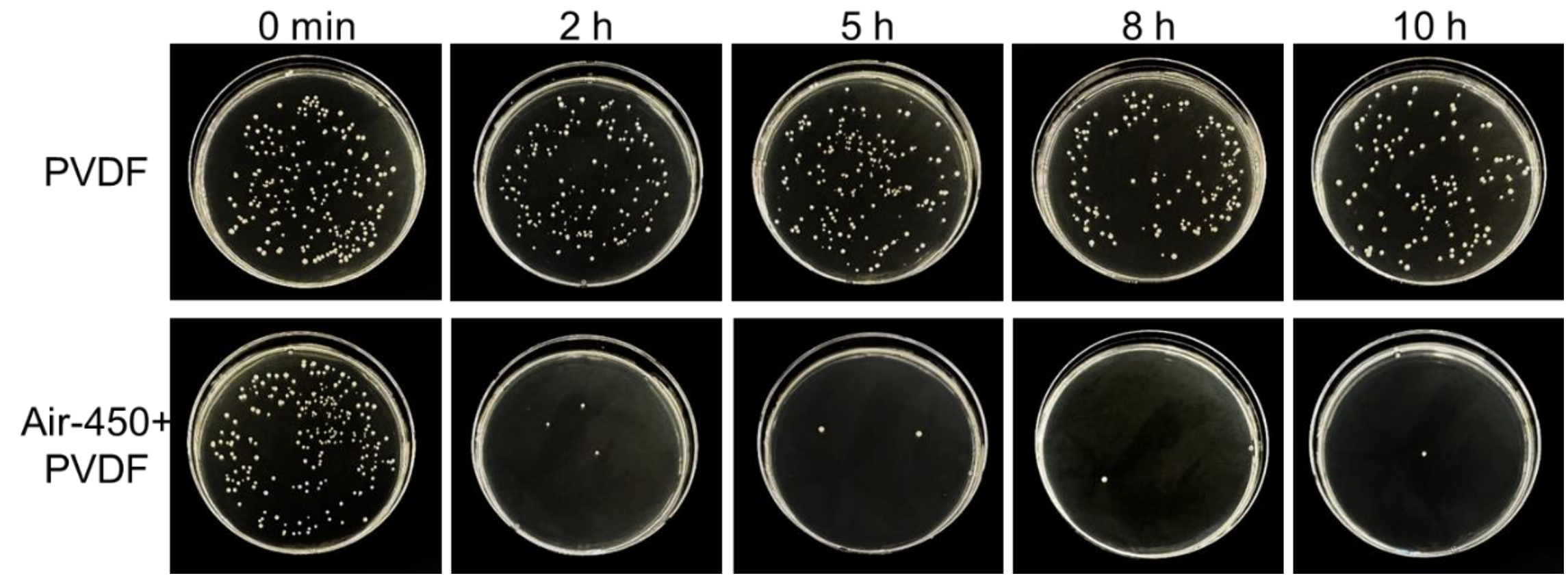

Figure S41. Digital photographs of *E. coli* DH5α from the effluent samples collected at specified time points for PVDF and Air-450+PVDF membranes.

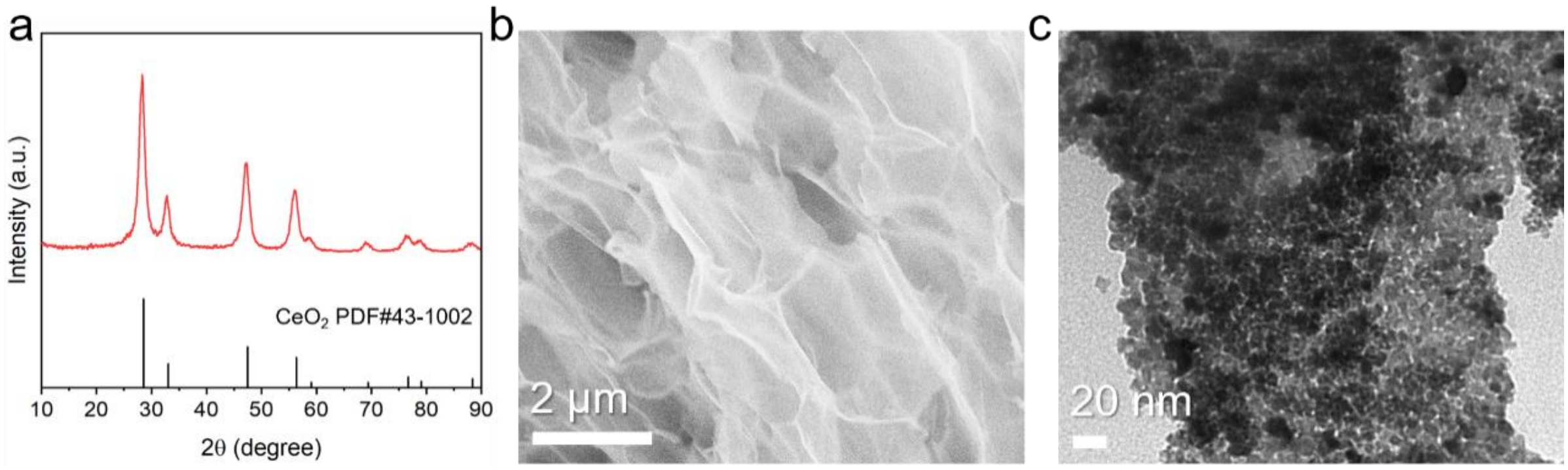

Figure S42. (a) XRD, (b) SEM, and (c) TEM results for Air-450 after 10 h of continuous-flow reaction.

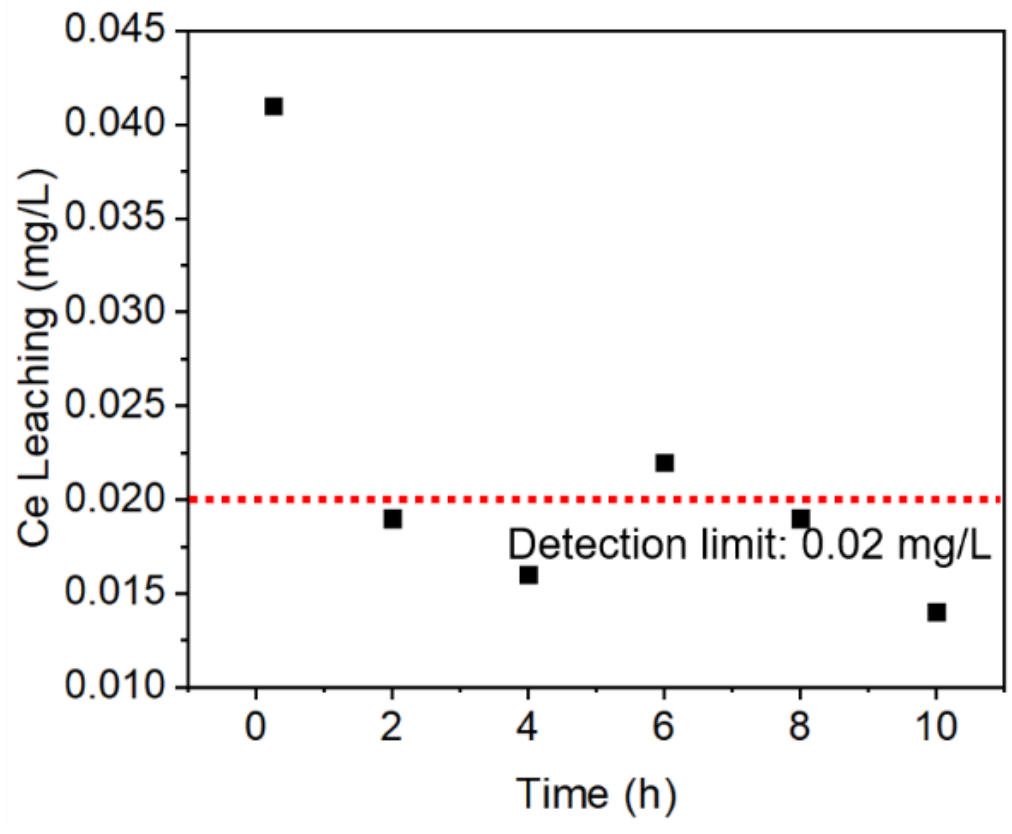

Figure S43. Ce leaching analysis (by ICP-OES) of effluent solutions collected during 10 h of continuous-flow reaction.

Table S1. Ce concentration in samples determined by inductively coupled plasma optical emission spectrometry (ICP-OES). The higher Ce concentrations obtained at higher calcination temperatures are attributed to the decomposition of the $Ce(NO_3)_3$ and PVP mixture, as supported by the TGA results in **Figure S7**.

Calcination Temperature (°C)	Ce Concentration (mg/mL)	Standard Deviation
150	0.149	0.012
250	0.172	0.005
350	0.389	0.005
450	0.581	0.004
550	0.594	0.011
650	0.600	0.006
750	0.603	0.006

Table S2. Production cost and cost-effectiveness analysis of the samples and BPO. The conversion efficiencies from raw materials to products were determined based on the mass of residue samples after calcination in air, as determined by TGA analysis **(Figure S7)**. Raw materials expenses include $Ce(NO_3)_3$ (67.30 $/kg) and PVP (91.33 $/kg) for producing 1 kg of samples. Electricity expenses for calcination are based on a 16 kW furnace and the electricity rates from Hong Kong China Light & Power (CIP). The production cost is the sum of raw material and electricity expenses. The cost-effectiveness of each sample and BPO was calculated by normalizing their *k* constant for RhB degradation against the production cost.

Sample	Quantities of raw materials (g)	Conversion efficiencies (%)	Synthesis of 1 kg sample			Production cost ($/kg)	k constant ($*10^{-1}$ min^{-1})	Cost-effectiveness ($*10^{-4}$ min^{-1}*kg/$)
			Raw materials expenses ($)		Electricity expenses ($)			
			$Ce(NO_3)_3$	PVP				
Air-350	120 g $Ce(NO_3)_3$ and 80 g PVP	49.05	82.32	74.48	3.33	160.14	0.66	4.12
Air-450		26.35	153.24	138.64	3.87	295.75	1.30	4.40
Air-550		26.15	154.42	139.70	4.40	298.52	0.88	2.95
Air-650		26.07	154.89	140.13	4.93	299.95	0.14	0.47
BPO	/	/	/		/	5.00×10^{9} (Sigma Aldrich)	0.85	1.70×10^{-7}

References

1. Y. Zheng, Z. Yu, H. Ou, et al., "Black Phosphorus and Polymeric Carbon Nitride Heterostructure for Photoinduced Molecular Oxygen Activation," *Advanced Functional Materials* 28 (2018): 1705407. https://doi.org/10.1002/adfm.201705407.

2. J. Ning, B. Zhang, L. Siqin, et al., "Designing Advanced S-Scheme CdS QDs/La-Bi_2WO_6 Photocatalysts for Efficient Degradation of RhB," *Exploration* 3 (2023): 20230050. https://doi.org/10.1002/exp.20230050.

3. B. J. Dunn, K. R. Watts, T. Robbins, D. E. Cane, and C. Khosla, "Comparative Analysis of the Substrate Specificity of *trans*- versus *cis*-Acyltransferases of Assembly Line Polyketide Synthases," *Biochemistry* 53 (2014): 3796-3806. https://doi.org/10.1021/bi5004316.

4. S. Cheng, X. Zhang, X. Yang, et al., "The Multiple Role of Bromide Ion in PPCPs Degradation under UV/Chlorine Treatment," *Environmental Science & Technology* 52 (2018): 1806-1816. https://doi.org/10.1021/acs.est.7b03268.

5. D. B. Miklos, C. Remy, M. Jekel, et al., "Evaluation of Advanced Oxidation Processes for Water and Wastewater Treatment - A Critical Review," *Water Research* 139 (2018): 118-131. https://doi.org/10.1016/j.watres.2018.03.042.

6. U. von Gunten and Y. Oliveras, "Kinetics of the Reaction between Hydrogen Peroxide and Hypobromous Acid: Implication on Water Treatment and Natural Systems," *Water Research* 31 (1997): 900-906. https://doi.org/10.1016/s0043-1354(96)00368-5.

7. M. A. Boles, D. Ling, T. Hyeon, and D. V. Talapin, "The Surface Science of Nanocrystals," *Nature Materials* 15 (2016): 141-153. https://doi.org/10.1038/nmat4526.

8. C. Zhao and Y. Xu, "Theoretical Investigation of Dephosphorylation of Phosphate Monoesters on CeO_2(111)," *Catalysis Today* 312 (2018): 141-148. https://doi.org/10.1016/j.cattod.2018.02.033.

9. K. Tang, M. Escola Casas, G. T. H. Ooi, et al., "Influence of Humic Acid Addition on the Degradation of Pharmaceuticals by Biofilms in Effluent Wastewater," *International Journal of Hygiene and Environmental Health* 220 (2017): 604-610. https://doi.org/10.1016/j.ijheh.2017.01.003.

10. T. Entradas, S. Waldron, and M. Volk, "The Detection Sensitivity of Commonly Used Singlet Oxygen Probes in Aqueous Environments," *Journal of Photochemistry and Photobiology B: Biology* 204 (2020): 111787. https://doi.org/10.1016/j.jphotobiol.2020.111787.

11. L. M. Shannon, E. Kay, and J. Y. Lew, "Peroxidase Isozymes from Horseradish Roots: I. Isolation and Physical Properties," *Journal of biological chemistry* 241 (1966): 2166–2172. https://doi.org/10.1016/S0021-9258(18)96680-9.

12. R. Wever, B. E. Krenn, and R. Renirie, "Marine Vanadium-Dependent Haloperoxidases, Their Isolation, Characterization, and Application," *Methods in Enzymology* 605 (2018): 141–201. https://doi.org/10.1016/bs.mie.2018.02.026.
